# Regulation of epithelial transitional states in murine and human pulmonary fibrosis

**DOI:** 10.1172/JCI165612

**Published:** 2023-11-15

**Authors:** Fa Wang, Christopher Ting, Kent A. Riemondy, Michael Douglas, Kendall Foster, Nisha Patel, Norihito Kaku, Alexander Linsalata, Jean Nemzek, Brian M. Varisco, Erez Cohen, Jasmine A. Wilson, David W.H. Riches, Elizabeth F. Redente, Diana M. Toivola, Xiaofeng Zhou, Bethany B. Moore, Pierre A. Coulombe, M. Bishr Omary, Rachel L. Zemans

**Affiliations:** 1 Division of Pulmonary and Critical Care Medicine, Department of Internal Medicine, University of Michigan, Ann Arbor, Michigan, USA.; 2 RNA Bioscience Initiative, University of Colorado Anschutz Medical Campus, Aurora, Colorado, USA.; 3 College of Literature, Science, and the Arts,; 4 Program in Cellular and Molecular Biology, School of Medicine, and; 5 Unit for Laboratory Animal Medicine, School of Medicine, University of Michigan, Ann Arbor, Michigan, USA.; 6 Division of Critical Care Medicine, Cincinnati Children’s Hospital Medical Center, Cincinnati, Ohio, USA.; 7 Department of Pediatrics, University of Cincinnati College of Medicine, Cincinnati, Ohio, USA.; 8 Department of Cell and Developmental Biology, University of Michigan, Ann Arbor, Michigan, USA.; 9 Program in Cell Biology, Department of Pediatrics, National Jewish Health, Denver, Colorado, USA.; 10 Division of Pulmonary Sciences and Critical Care Medicine, Department of Medicine, University of Colorado School of Medicine, Aurora, Colorado, USA.; 11 Department of Research, Veterans Affairs Eastern Colorado Health Care System, Denver Colorado, USA.; 12 Cell Biology, Biosciences, Faculty of Science and Engineering, and InFLAMES Research Flagship Center, Åbo Akademi University, Turku, Finland.; 13 Department of Microbiology and Immunology, University of Michigan, Ann Arbor, Michigan, USA.; 14 Department of Medicine, Robert Wood Johnson Medical School, New Brunswick, New Jersey, USA.

**Keywords:** Pulmonology, Adult stem cells, Fibrosis

## Abstract

Idiopathic pulmonary fibrosis (IPF) is a progressive scarring disease arising from impaired regeneration of the alveolar epithelium after injury. During regeneration, type 2 alveolar epithelial cells (AEC2s) assume a transitional state that upregulates multiple keratins and ultimately differentiate into AEC1s. In IPF, transitional AECs accumulate with ineffectual AEC1 differentiation. However, whether and how transitional cells cause fibrosis, whether keratins regulate transitional cell accumulation and fibrosis, and why transitional AECs and fibrosis resolve in mouse models but accumulate in IPF are unclear. Here, we show that human keratin 8 (*KRT8*) genetic variants were associated with IPF. *Krt8^–/–^* mice were protected from fibrosis and accumulation of the transitional state. Keratin 8 (K8) regulated the expression of macrophage chemokines and macrophage recruitment. Profibrotic macrophages and myofibroblasts promoted the accumulation of transitional AECs, establishing a K8-dependent positive feedback loop driving fibrogenesis. Finally, rare murine transitional AECs were highly senescent and basaloid and may not differentiate into AEC1s, recapitulating the aberrant basaloid state in human IPF. We conclude that transitional AECs induced and were maintained by fibrosis in a K8-dependent manner; in mice, most transitional cells and fibrosis resolved, whereas in human IPF, transitional AECs evolved into an aberrant basaloid state that persisted with progressive fibrosis.

## Graphical abstract

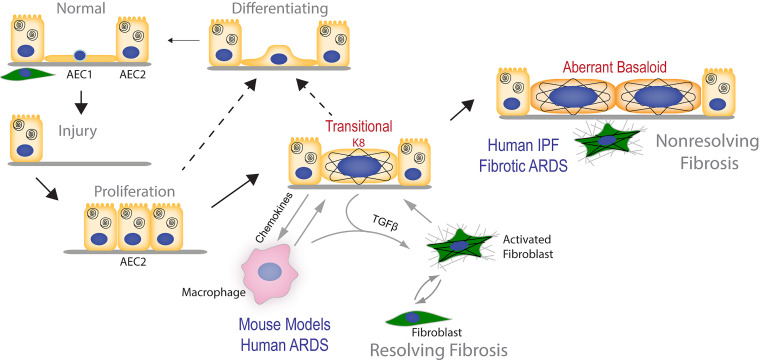

## Introduction

Idiopathic pulmonary fibrosis (IPF) is a disease of aging in which progressive scarring of the lungs leads to death from respiratory failure (1). Highly effective treatments are lacking, in part because the disease pathogenesis is incompletely understood. IPF is believed to arise from impaired regeneration of the alveolar epithelium after injury. Impaired epithelial regeneration leads to the activation of fibroblasts. Activated fibroblasts deposit matrix and contract, which increases lung stiffness and impairs gas exchange, resulting in respiratory failure (1, 2). However, the specific defect in epithelial regeneration and the mechanisms by which impaired epithelial regeneration causes fibrosis have remained elusive.

Independent, robust lines of investigation have uncovered several pathologic processes activated in epithelial cells that drive fibrosis: senescence, expression of macrophage chemokines, integrin β6–dependent TGF-β activation, impaired proteostasis (endoplasmic reticulum stress), DNA damage, and cell death (2–25). Senescence is a state of cell-cycle arrest characterized by the secretion of chemokines, cytokines, growth factors, and proteases, termed the senescence-associated secretory phenotype (SASP) (26–32). Classically, senescence is permanent and drives diseases of aging, although an emerging paradigm distinguishes a transient senescence with a role in physiologic regeneration (29, 32, 33). Macrophage chemokines recruit profibrotic macrophages, which drive fibrogenesis (2, 19–21, 23, 24, 34–42). Epithelial cell integrin β6–dependent TGF-β activation induces fibroblast activation (2, 17, 18). However, how these diverse profibrotic processes relate to impaired epithelial regeneration long remained unclear.

Alveolar epithelial type 1 cells (AEC1s) cover 98% of the alveolar surface (43) and mediate efficient gas exchange, whereas AEC2s produce surfactant and serve as progenitors. During physiologic regeneration after AEC injury, AEC2s and other progenitors proliferate and differentiate into AEC1s (44–49). Using scRNA-Seq, we identified a transitional cell state that AEC2s assume during regeneration after lung injury (50). This state is characterized by upregulation of markers of cell-cycle arrest, downregulation of AEC2 markers, modest upregulation of AEC1 markers, and high expression of unique signature genes including multiple keratin genes, *Cldn4*, *Sfn*, and TGF-β pathway genes including integrin β6 (50). We and others confirmed that this keratin^hi^ state arises from AEC2s and other progenitors and after lung injury from diverse causes (48, 50–56). Subsequent lineage-tracing studies demonstrated that during regeneration in mice, transitional cells can differentiate into AEC1s (52, 53), restoring normal alveolar architecture. However, our scRNA-Seq data suggested that some transitional cells may not have an AEC1 fate (50). Regardless, AEC2-to-AEC1 differentiation may be a nongradual process, pausing in this discrete transitional state (50, 53, 54), in contrast to the continuous differentiation observed in other organs. The mechanisms underlying this nongradual cell differentiation are unknown.

We and others subsequently discovered that the accumulation of keratin^hi^ transitional cells with ineffectual AEC1 differentiation may be the specific regenerative defect underlying the pathogenesis of human IPF (48, 50–59). The transitional AECs in human IPF have been described as basaloid due to their transcriptomic resemblance to airway basal cells (57, 58, 60). TGF-β (50, 51), IL-1β (52), and the integrated stress response (61–63) have been implicated in promoting the transitional state in fibrosis. However, the mechanisms by which transitional cells accumulate and promote fibrosis remain incompletely understood. Moreover, why transitional cells ultimately differentiate into AEC1s with resolution of fibrosis in mouse models (and presumably in patients who recover normal lung function after acute lung injury; ref. 64) but accumulate with progressive fibrosis in human IPF remains a fundamental unanswered question with important clinical implications.

Keratins are intermediate filaments expressed in epithelia. Since the specific keratins expressed depend on the cell type and state of differentiation, keratins are commonly used as “markers” of cell differentiation (65, 66). However, their functions are often overlooked. Keratins are known for conferring mechanical stability via organization into a stiff, filamentous network (66, 67). Yet, keratins also have nonmechanical functions, including regulating cell differentiation, cell survival, and inflammation, which are less well understood (66, 68–70). Specific keratins have been used as markers of the AEC transitional state (51, 54, 58, 62). However, whether keratins play a functional role in accumulation of transitional cells and fibrogenesis is unknown.

Here, we tested the hypothesis that keratins regulate fibrosis and the accumulation of transitional cells. We further hypothesized that murine and human transitional cells consist of 2 subsets, one that is transient and capable of AEC1 differentiation, and another that is highly senescent and may not differentiate; whereas in mice, many transitional AECs differentiate into AEC1s with resolution of fibrosis; in human IPF, transitional cells evolve into the highly senescent state, fail to differentiate into AEC1s, and drive progressive fibrosis.

## Results

### Keratin^hi^ transitional cells are conserved across mouse models and human IPF and activate profibrotic processes.

**Figure 1:**
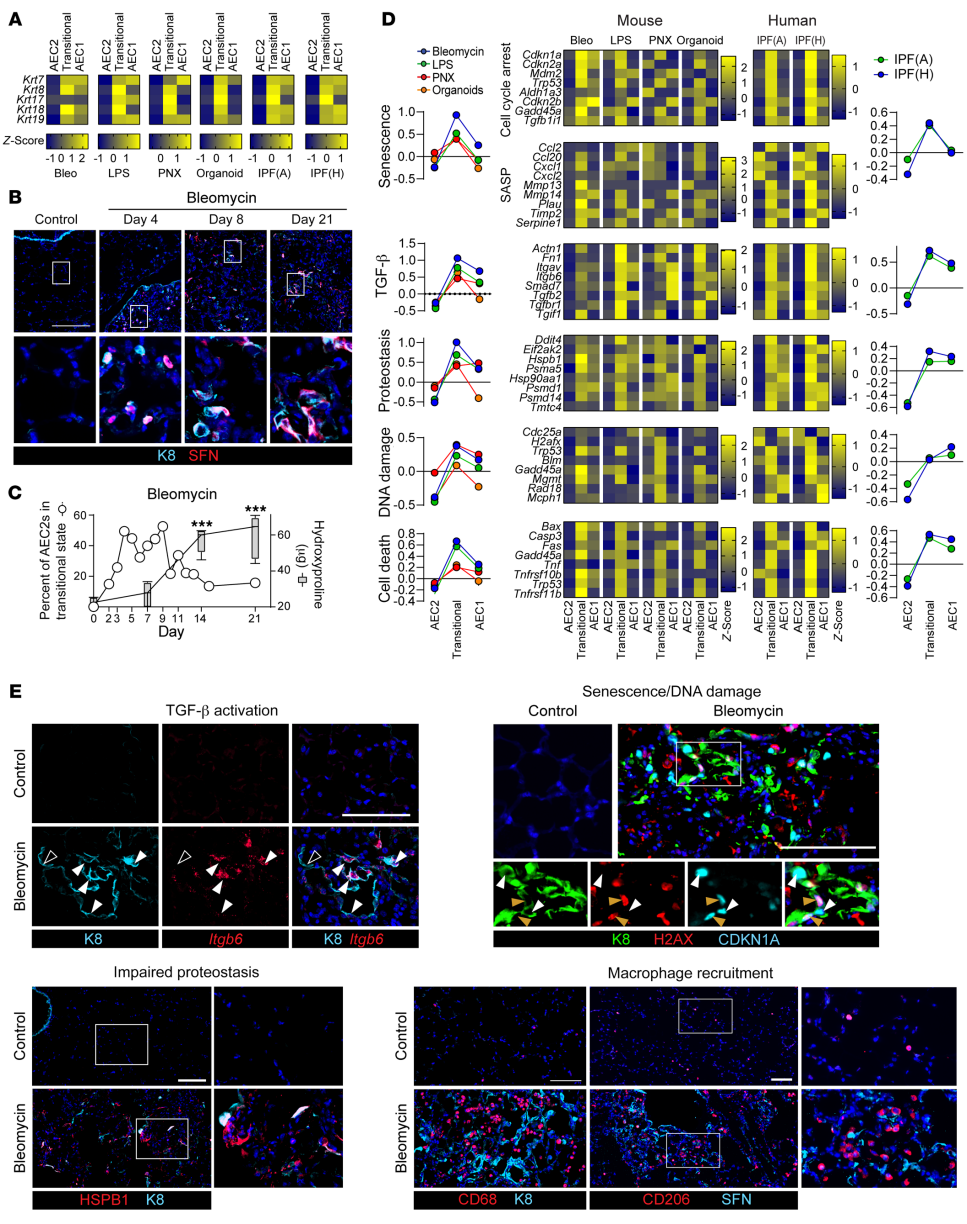
Conserved keratin^hi^ transitional AECs arise prior to fibrosis and activate profibrotic processes. (**A**, **C**, and **D**) scRNA-Seq data sets from the bleomycin (54), LPS (50), pneumonectomy (56), and organoid (53) mouse models and human IPF (57, 58) were analyzed. (**B**, **C**, and **E**) Mice were treated with bleomycin. (**A**) Several keratins were upregulated in the transitional state in multiple mouse models and in human IPF. (**B** and **C**) Transitional cells arose prior to fibrosis. (**D**) Scores indicating activation of each profibrotic pathway were calculated on the basis of canonical gene expression. The expression of genes representative of each pathway are shown by heatmaps (**D**) and immunostaining or FISH (**E**). Solid white arrowheads indicate transitional cells showing activation of a given pathway. Open arrowheads indicate rare K8^hi^ cells without *Itgb6* staining. Orange arrowheads indicate CDKN1A^+^H2AX^+^ cells. Multiple profibrotic pathways, senescence, TGF-β, impaired proteostasis, DNA damage, and cell death were uniquely and concurrently activated in the transitional cell state in multiple mouse models and human IPF. Scale bars: 100 μm. Original magnification, ×20 (enlarged insets in **B** and **E**). (**B** and **E**) *n* = 3 mice/group; (**C**) *n* = 5 mice/group (hydroxyproline). Hydroxyproline data are represented as box-and-whisker plots, with the box (25th to 75th percentiles), median (line), and whiskers (minimum to maximum). (**C**) ***P < 0.001 compared with day 0 by 1-way ANOVA with post hoc Bonferroni’s test.

The AEC transitional state arises in diverse mouse models of injury and regeneration and in human IPF (48, 50–58). Although *Krt8* has been highlighted as a transitional state marker in mice (51, 54) and *KRT17* in human IPF (58), *KRT7*, *KRT8*, *KRT17*, *KRT18*, and *KRT19* were upregulated in the transitional state in the bleomycin, LPS, pneumonectomy (PNX), and organoid mouse models of regeneration and in human IPF (Figure 1A). Moreover, the transcriptome of the transitional state was conserved across diverse etiologies of injury and species (Supplemental Figure 1, A and B; supplemental material available online with this article; https://doi.org/10.1172/JCI165612DS1) (48, 50–59, 71–73), underscoring the pivotal role of this cell state in alveolar regeneration and suggesting that mouse models are relevant to human IPF. However, there are some differences, particularly between murine and human transitional cells (Supplemental Figure 1, A and B), the implication of which is poorly understood.

Fibrosis is characterized by the accumulation of transitional AECs with ineffectual AEC1 differentiation, suggesting that the critical regenerative defect underlying the pathogenesis of IPF may be persistence of the transitional state (51–58). However, whether the accumulation of transitional cells causes fibrosis or vice versa is unknown. In the bleomycin model, transitional cells arose by day 2, whereas fibrosis was detected by day 7 after bleomycin (Figure 1, B and C, and Supplemental Figure 1C). Whereas the PNX and LPS models had minimal fibrosis and rare transitional cells, the bleomycin model was characterized by extensive fibrosis and abundant transitional cells (Supplemental Figure 1, D and E). That transitional cells arose prior to fibrosis and in proportion to the extent of fibrosis supports the notion that they are instrumental in the development of fibrosis.

An extensive body of work has established that epithelial cell senescence, impaired proteostasis, cell death, DNA damage, integrin β6–mediated TGF-β activation, and macrophage chemokine expression promote fibrosis (2–22). However, how these pathologic processes relate to impaired epithelial regeneration has remained unclear. Pathway analysis of the top differentially expressed genes (DEGs) in the transitional state, validated by the expression of individual canonical genes of each pathway, confirmed that these profibrotic processes were uniquely activated in transitional cells (Figure 1, D and E, and Supplemental Figure 1, F–H). These data unify multiple independent lines of investigation into the mechanisms by which epithelial cells are profibrotic and suggest that transitional AECs promote fibrosis.

### Keratin 8 is necessary for the accumulation of transitional AECs and fibrosis.

**Figure 2:**
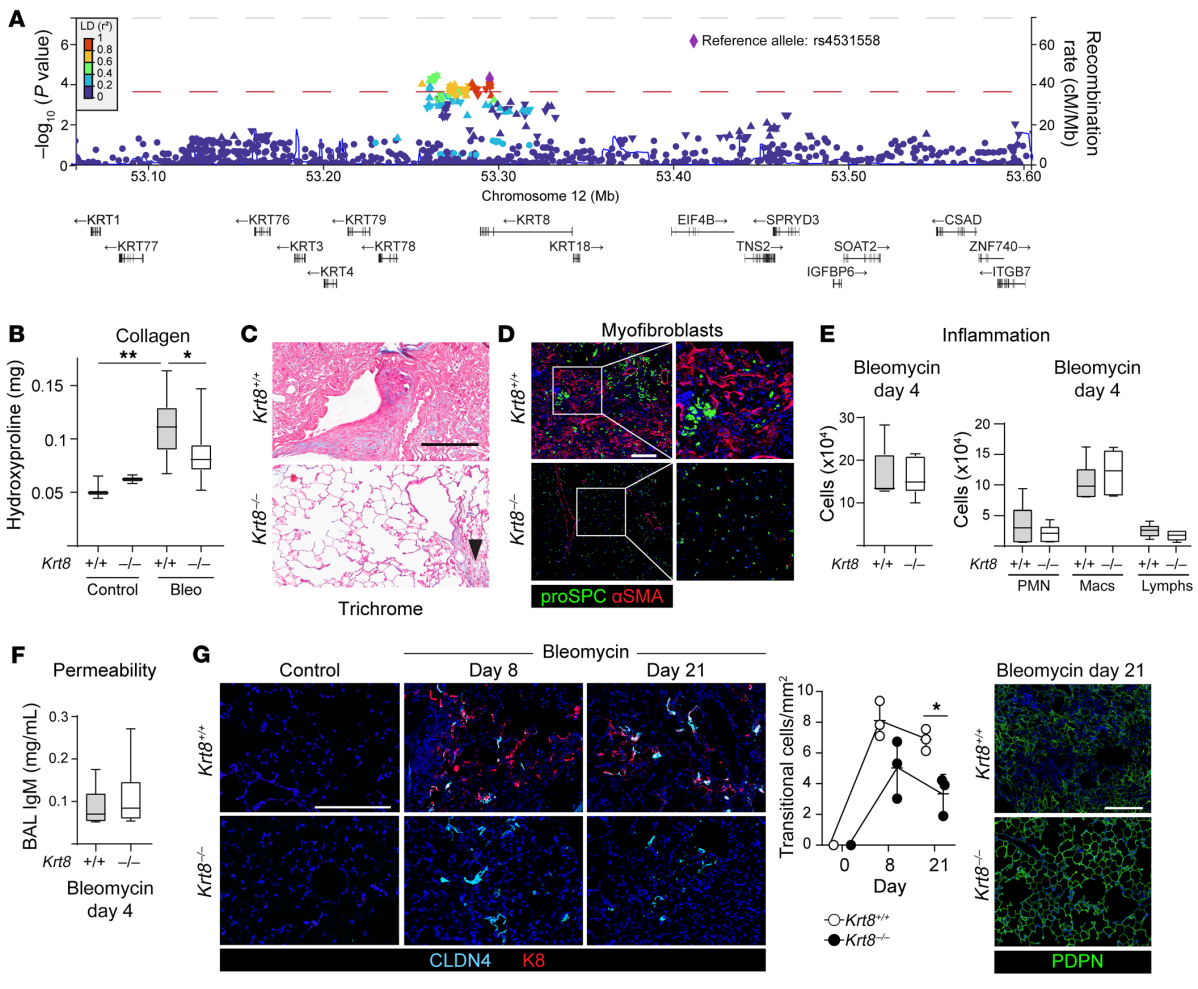
K8 promotes fibrosis and accumulation of transitional AECs. (**A**) Using a genome-wide association meta-analysis of IPF (75), a nested candidate gene study for the keratins expressed in the transitional state was performed. Regional association plot showing all SNPs that overlap with *KRT8*. Gray dotted line indicates genome-wide significance; red dotted line indicates statistical significance of the nested candidate gene study for the keratin genes; blue curve indicates the estimated recombination rate. Seven *KRT8* SNPs were associated with IPF (*P* < 1.4 × 10^–4^). The most significant variant, rs4531558 (*P* = 5.2 × 10^–5^), shown as a purple diamond, is in linkage disequilibrium (LD) (*R^2^* > 0.8) with all other statistically significant variants. (**B**–**G**) *Krt8^+/+^* and *Krt8^–/–^* mice were treated with bleomycin. *Krt8^–/–^* mice were protected from fibrosis, as determined by hydroxyproline assay (**B**), trichrome staining (**C**), and myofibroblast accumulation (**D**). Arrowhead in **C** indicates a small area of fibrosis. *Krt8^–/–^* mice were not protected from lung injury at day 4, as determined by inflammation (**E**) and permeability (**F**). (**G**) Compared with *Krt8^–/–^* mice, transitional cells accumulated with incomplete AEC1 regeneration in *Krt8^+/+^* mice. (**B**, **E**, and **F**) Data are represented as box-and-whisker plots, with box (25th to 75th percentiles), median (line), and whiskers (minimum to maximum). *P < 0.05 and **P < 0.01, by 1-way ANOVA with post hoc Bonferroni’s test. (**G**) Data indicate the mean ± SD. *P < 0.05, by 2-way ANOVA with post hoc Šidák’s multiple-comparison test. Scale bars: 200 μm. Original magnification, ×20 (enlarged insets in **D**). *n* = 3 mice/group except bleomycin-treated mice in **B**, *n* = 14 mice/group and **E**, *n* = 6 mice/group.

To further explore whether transitional cells promote fibrosis and whether keratins play a functional role in the accumulation of transitional cells and fibrogenesis, we interrogated a meta-analysis of 5 human IPF GWAS studies (74) for variants in the keratin genes highly expressed by transitional AECs: *KRT7*, *KRT8*, *KRT17*, *KRT18*, and *KRT19*. Only *KRT8* was associated with IPF (Figure 2A, Supplemental Figure 2, and Supplemental Table 1). The associated *KRT8* SNPs had high Combined Annotation-Dependent Depletion (CADD) and Regulatory Mendelian Mutation (REMM) scores, which predict disease causality of genetic variants. These results suggest that *KRT8* genetic variants may be pathogenic. Therefore, we hypothesized that keratin 8 (K8) promotes the accumulation of transitional cells and fibrosis.

Since transitional cells arise from AEC2s and other progenitors (48, 50–54, 56–58, 60), we reasoned that the best strategy to assess the role of K8 in alveolar regeneration and fibrosis was to use global *Krt8*-KO mice as long as *Krt8* deficiency did not alter lung development or homeostasis. Adult *Krt8^–/–^* mice appeared to have normal lung structure without inflammation or altered expression of other keratins (Supplemental Figure 3, A–H), suggesting that K8 is not necessary for lung development or homeostasis. By 3 months of age, male *Krt8^–/–^* mice weighed less than *Krt8^+/+^* mice (Supplemental Figure 3I), probably due to intestinal pathology (70), but otherwise appeared healthy.

To determine whether K8 is necessary for transitional cell accumulation and fibrosis, we treated *Krt8^–/–^* mice with bleomycin. We found that *Krt8^–/–^* mice were protected from fibrosis (Figure 2, B–D, and Supplemental Figure 3J), but these mice were not protected from inflammation or permeability during the injury phase of the bleomycin model (Figure 2, E and F), suggesting that protection against fibrosis was mediated by a role for K8 in the repair phase, consistent with its upregulation as progenitors assume the transitional state. Since keratins regulate cell differentiation in other organs (68) and *Krt8* is downregulated as transitional cells differentiate into AEC1s (Figure 1A), we hypothesized that K8 promotes the accumulation of transitional AECs at the expense of AEC1 differentiation. To address this question, we quantified transitional cells over time in *Krt8^+/+^* and *Krt8^–/–^* mice. Indeed, we found that transitional AECs accumulated with incomplete AEC1 regeneration in *Krt8^+/+^* mice at day 21 after bleomycin treatment, whereas transitional cells had largely resolved with restoration of AEC1s in *Krt8^–/–^* mice (Figure 2G). Hence, *Krt8^–/–^* mice were protected from fibrosis and the accumulation of profibrotic transitional cells at the expense of AEC1 differentiation.

### K8 is necessary for macrophage chemokine expression.

**Figure 3:**
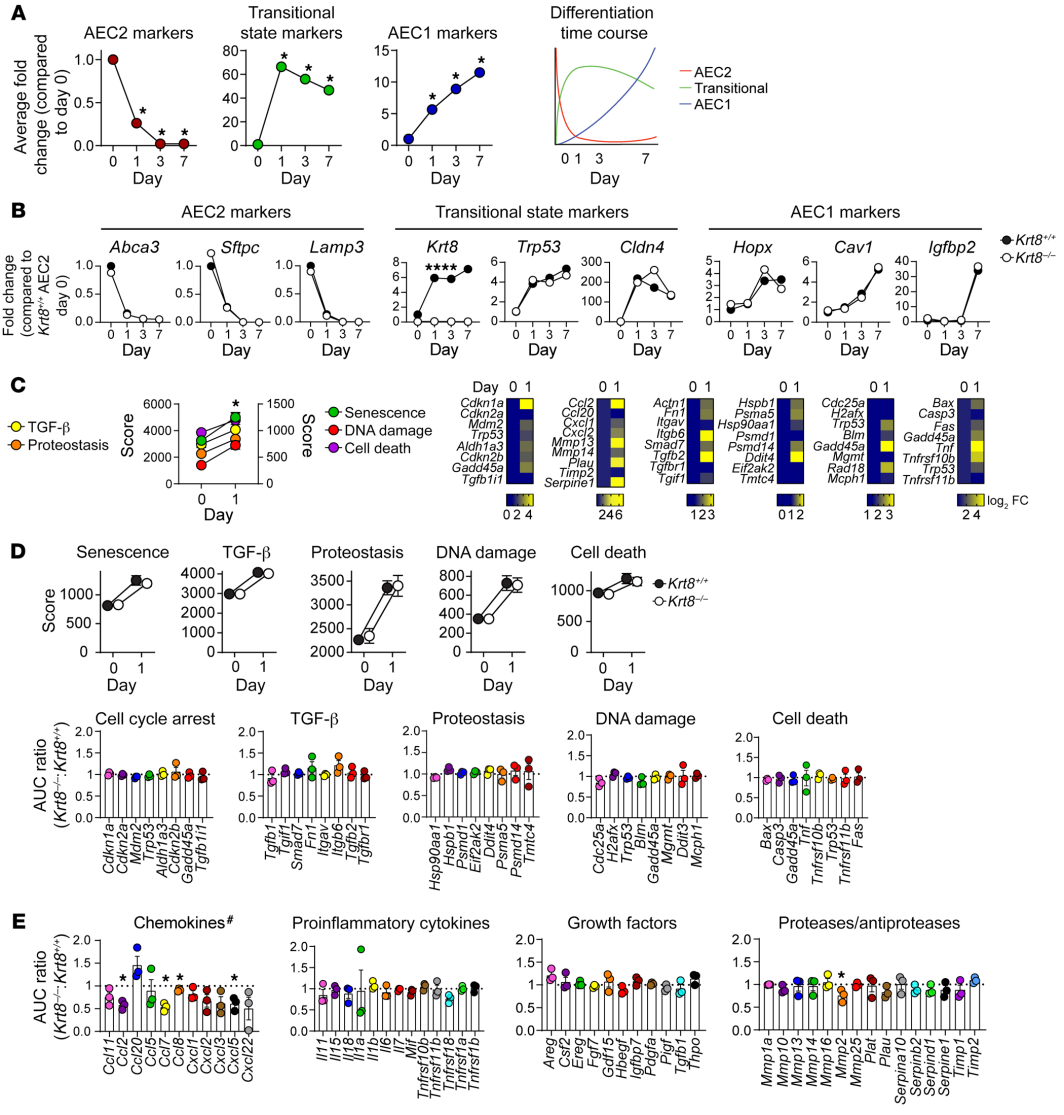
K8 promotes the expression of chemokines but not the accumulation of transitional cells at the expense of AEC1 differentiation. AEC2s were isolated from *Krt8^+/+^* and *Krt8^–/–^* mice and cultured in 2D. RNA-Seq was performed. (**A**) Average fold change (FC) of composite AEC2, transitional state, or AEC1 marker scores (see also Supplemental Table 2) compared with day 0 for WT AECs. *P < 0.05 compared with day 0. AEC culture recapitulates in vivo stages of alveolar regeneration, as shown by downregulation of AEC2 markers and upregulation of transitional state markers on day 1 of culturing and a gradual upregulation of AEC1 markers by day 7. Far-right panel is a schematic representation of the data. (**B**) *Krt8* deficiency had no effect on transitional cell or AEC1 differentiation. ****P < 0.0001, by unpaired *t* test on the AUC from days 1–7 for *Krt8^+/+^* versus *Krt8^–/–^* cells. (**C**) Markers of senescence, TGF-β activation, impaired proteostasis, DNA damage, and cell death were upregulated in the transitional state in vitro. *P ≤ 0.05 by *t* test for day 1 compared with day 0 for all pathway scores. *P* values for genes in heatmaps are listed in Supplemental Table 3. (**D**) K8 was not necessary for upregulation of markers of cell-cycle arrest, TGF-β activation, impaired proteostasis, DNA damage, and cell death. For individual genes, the ratio of AUC of expression from days 1–7 in *Krt8^–/–^* versus *Krt8^+/+^* cells is shown. (**E**) K8 was necessary for the expression of SASP chemokines but not proinflammatory cytokines, growth factors, or proteases/antiproteases. ^#^*P* < 0.05, by *t* test of the average of the ratio of AUCs of all chemokines; *P < 0.05, by *t* test for individual genes. *n* = 3. All data are presented as the mean (**A** and **B**) or the mean ± SEM (**C**–**E**).

In vivo models are complex because of crosstalk between multiple cell types that can have both direct and indirect effects. Therefore, to determine whether K8 directly regulates AEC differentiation, promoting accumulation of the transitional state and impeding AEC1 differentiation, we cultured AECs under 2D conditions in which AEC2s are known to differentiate into “AEC1-like” cells (50, 75–79). We found that, while AEC1 markers were gradually upregulated by day 7 of culture, cells assumed the transitional state at day 1 (Figure 3A and Supplemental Figure 4A). Thus, culturing AEC2s in 2D recapitulates the transitional and AEC1 differentiation stages of alveolar regeneration observed in vivo and can therefore be used as a model system with which to examine the mechanisms that regulate cell differentiation. On the basis of the in vivo phenotype (Figure 2G), we hypothesized that K8 would promote the accumulation of transitional AECs at the expense of AEC1 differentiation in vitro. Surprisingly, we found that *Krt8^–/–^* and *Krt8^+/+^* AECs exhibited no significant difference in cell differentiation (Figure 3B and Supplemental Figure 4B). There was also no significant difference in the acquisition of the mature (*Igfbp2^+^*) AEC1 state (Supplemental Figure 4B) in which 95% of adult AEC1s exist (80). Gross differences between *Krt8^–/–^* and *Krt8^+/+^* AECs in cell spreading during AEC1 differentiation were not detected (Supplemental Figure 4C). Taken together, these data suggest that the function of K8 in the accumulation of profibrotic transitional cells (Figure 2G) may depend on the in vivo milieu.

**Figure 4:**
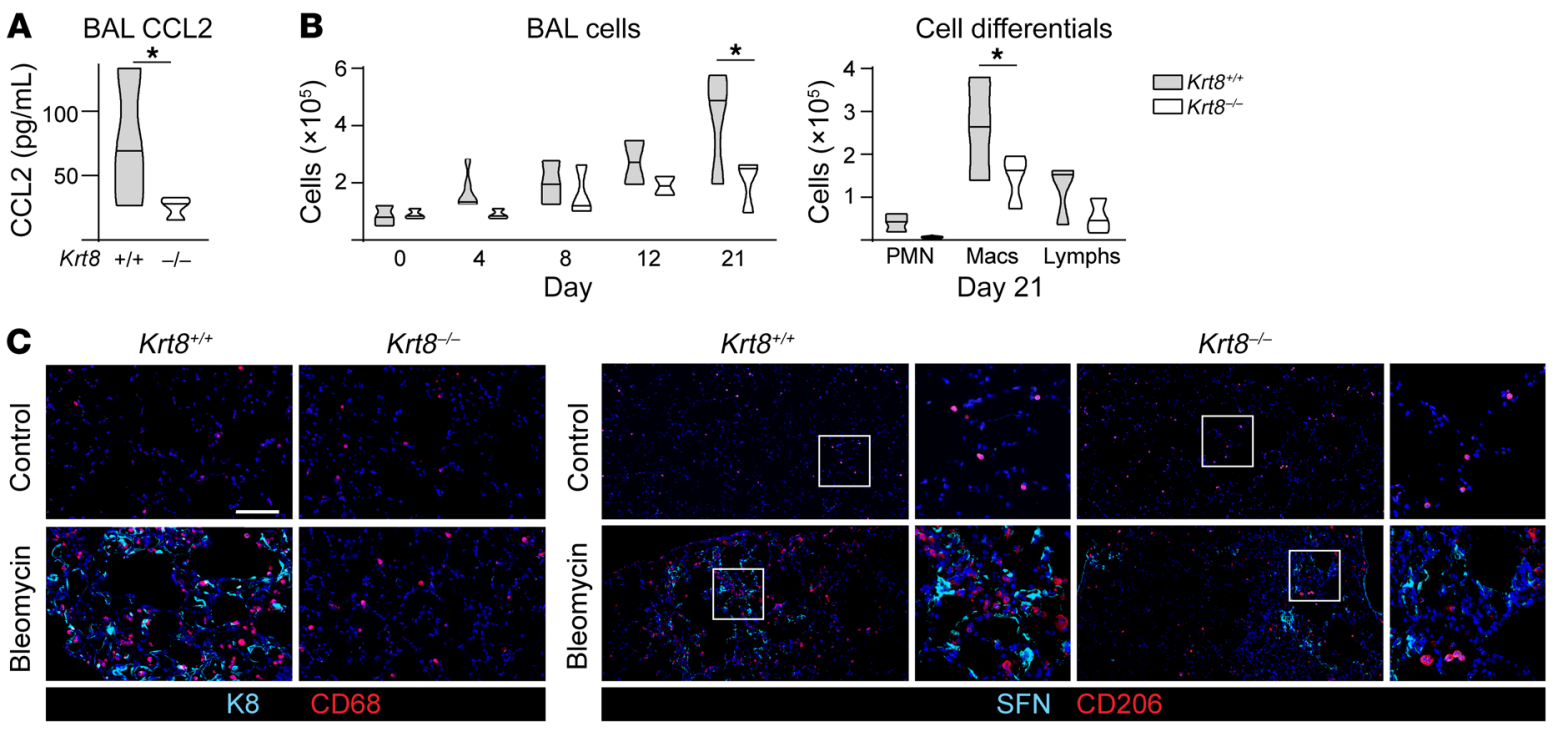
K8 is necessary for macrophage chemokine expression and macrophage recruitment during fibrosis. *Krt8^+/+^* and *Krt8^–/–^* mice were treated with bleomycin. (**A**) CCL2 ELISA on day 21 bronchoalveolar lavage (BAL). *n* = 3 mice/group. (**B**) BAL cells and differentials. *n* = 3 mice/group except for day 4 (*n* = 6 mice/group) and day 12 (*n* = 2 mice/group). Macs, macrophages; Lymphs, lymphocytes; PMN, polymorphonuclear neutrophils. (**A** and **B**) Violin plots show minimum to maximum values with the line at the median. *P < 0.05, by ratio paired *t* test. *n* = 4–5 mice/group. (**C**) Immunostaining on day-12 tissue. The top left image of the left panel and the top left image of the right panel in **C** are also shown in Figure 1E (macrophage recruitment control, left and middle images). *n* = 3 mice/group. Scale bar: 100 μm. Original magnification, ×20 (enlarged insets in **C**).

We next asked whether K8 may promote fibrosis by regulating the profibrotic processes activated in transitional cells. As AEC2s assumed the transitional state in culture, they recapitulated the senescence (cell-cycle arrest and SASP), impaired proteostasis, cell death, DNA damage, and integrin β6–mediated TGF-β activation that characterize the transitional state in vivo (Figure 3C). To determine whether K8 promotes fibrosis by driving these profibrotic processes, we assessed their activation in *Krt8^–/–^* transitional cells. Loss of *Krt8* did not affect senescence, proteostasis, cell death, DNA damage, or TGF-β activation, as determined by the composite expression scores of genes associated with each process (Figure 3D). To assess the expression of individual genes by transitional cells throughout the culturing, we calculated the AUC of expression levels from day 1 to day 7 of culturing in *Krt8^–/–^* and *Krt8^+/+^* AECs. Relative gene expression was then determined by the ratio of the AUC of gene expression in *Krt8^–/–^* AECs to that in *Krt8^+/+^* AECs (Figure 3D). No differences in the expression of markers of cell-cycle arrest, TGF-β activation, impaired proteostasis, DNA damage, or cell death were detected. In fact, although gene expression evolved over time as the cells differentiated in culture, the transcriptomes of *Krt8^–/–^* and *Krt8^+/+^* AECs remained highly similar (Supplemental Figure 4D). Since previous studies established that impaired proteostasis promotes the transitional state (61–63), we further tested the effect of K8 on proteostasis. We used shRNA to knock down *Krt8* in an AEC2 cell line, MLE-12, and stimulated the cells with tunicamycin to induce ER stress. Consistent with our transcriptomics analysis (Figure 3D), loss of *Krt8* did not attenuate ER stress (Supplemental Figure 4E). Finally, we examined whether K8 regulated the expression of SASP genes. We found that loss of *Krt8* did not affect the expression of most classes of SASP genes (proinflammatory cytokines, growth factors, and proteases/antiproteases), but did attenuate the expression of multiple chemokines (Figure 3E and Supplemental Figure 4F). These chemokines likely work in concert to recruit macrophages. We focused on CCL2 because it has been strongly implicated in promoting fibrosis through the recruitment of profibrotic macrophages (19, 20, 23–25, 38) and was significantly attenuated in *Krt8^–/–^* AECs. We confirmed that *Krt8* deficiency attenuated CCL2 expression (Figure 4A, Supplemental Figure 4G) and the recruitment of macrophages (Figure 4, B and C) during fibrogenesis in vivo. Taken together, these data are consistent with a paradigm in which K8 induces fibrosis via regulation of SASP genes, specifically chemokines, which promote the recruitment of profibrotic macrophages.

### Macrophages and fibroblasts promote the accumulation of transitional AECs.

**Figure 5:**
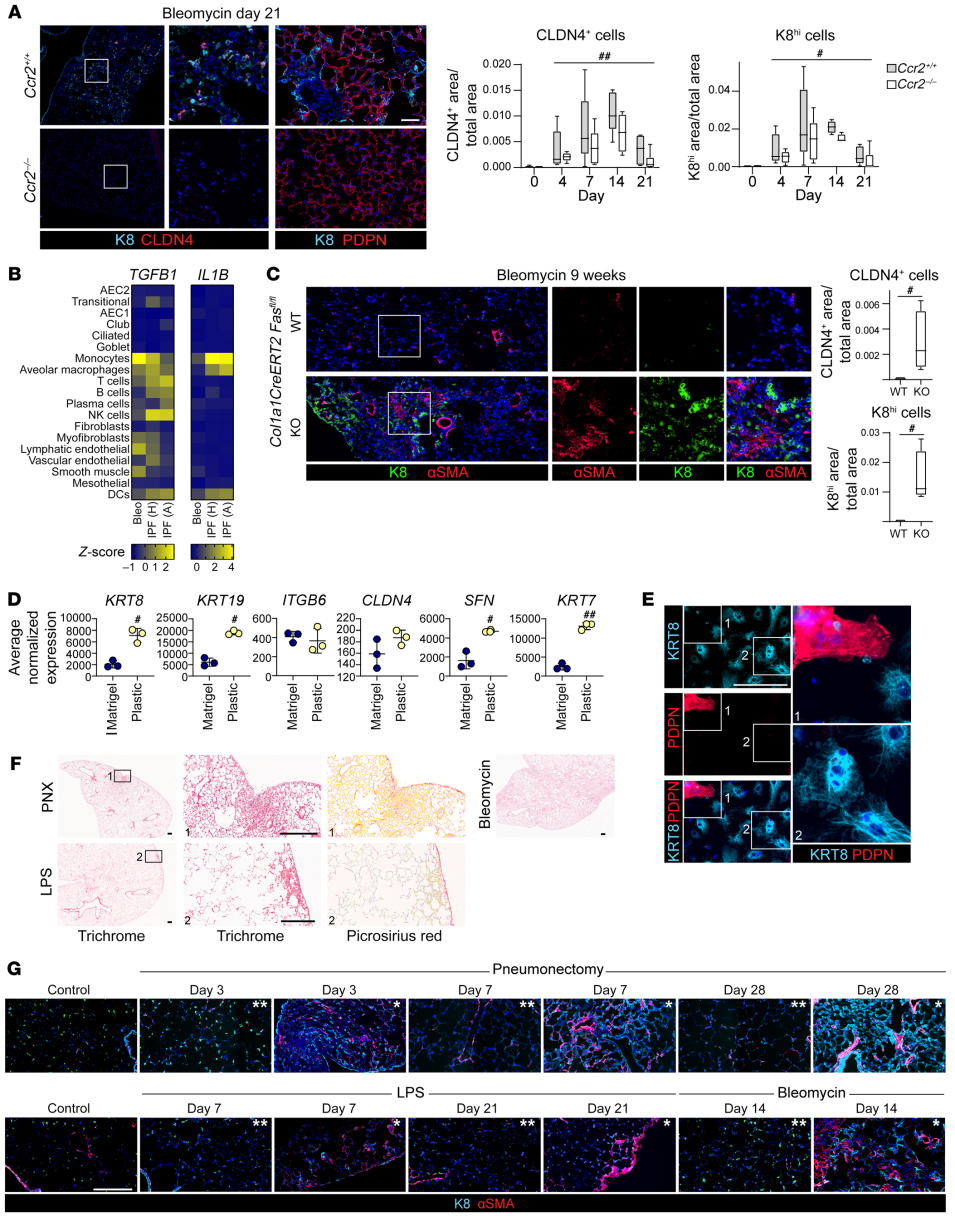
Macrophages and fibroblasts promote the accumulation of transitional AECs. (**A**) *Ccr2^+/+^* or *Ccr2^–/–^* mice were treated with bleomycin. Macrophage recruitment was necessary for transitional state accumulation. ^#^*P* < 0.05 and ^##^*P* < 0.01, by 2-way ANOVA for *Ccr2^+/+^* versus *Ccr2^–/–^* from days 4–21. *n* = 5 mice/group. (**B**) Macrophages and monocytes were a major source of TGF-β and IL-1β in murine and human fibrosis, as determined by scRNA-Seq (54, 58). Bleo, bleomycin. (**C**) *Col1a1CreERT2* *Fas^fl/fl^* mice were treated with bleomycin or administered tamoxifen (KO) or corn oil (WT), and euthanized at 9 weeks. Fibroblast-specific *Fas* knockout induced myofibroblast persistence, which was sufficient for persistence of the AEC transitional state. ^#^*P* < 0.05, by *t* test. *n* = 3 mice/group. (**D**) Gene expression by AECs cultured on Matrigel or collagen-coated plastic for 3 days. Collagen/stiff substrate promoted transitional state accumulation. Data represent the mean ± SD. ^#^*P* < 0.05 and ^##^*P* < 0.01, by paired *t* test. (**E**) Murine AEC2s were cultured in 2D and fixed and immunostained on day 7. Most cells persisted in the transitional state, with some AEC1 differentiation (*n* = 3). (**F** and **G**) Transitional cells were found in small foci of fibrosis in peripheral lung in the PNX and LPS mouse models (*), whereas most of the lung was devoid of fibrosis and transitional cells (**). In the bleomycin model, large areas of lung were characterized by fibrosis and transitional cells (*), whereas some areas were devoid of fibrosis and transitional cells (**). Scale bars: 50 μm. Original magnification, ×20 (enlarged insets in **A**, **C**, **E**, and **F**). For immunostaining, *n* = 3/group. PDPN, podoplanin; αSMA, α smooth muscle actin.

K8 was necessary for the accumulation of transitional cells at the expense of AEC1 differentiation in vivo (Figure 2G) but not in cultured AECs (Figure 3B and Supplemental Figure 4B), suggesting that cell-cell crosstalk and/or the fibrotic milieu may be necessary for the K8-dependent accumulation of transitional cells. Since K8 contributed to the recruitment of macrophages during fibrogenesis (Figure 4B), we considered whether macrophages may in turn contribute to the accumulation of transitional cells. Bleomycin was administered to mice in which macrophage recruitment to the lung was prevented by *Ccr2* KO (Supplemental Figure 5A). *Ccr2^–/–^* mice were protected from the accumulation of transitional AECs at the expense of AEC1 regeneration (Figure 5A and Supplemental Figure 5B). Since TGF-β (50, 51) and IL-1β (52) have been shown to promote accumulation of the AEC transitional state, we next hypothesized that recruited macrophages promote transitional state accumulation via TGF-β and IL-1β secretion. We confirmed that monocytes and macrophages were a major source of TGF-β and IL-1β during fibrosis (Figure 5B) (40, 52, 81). These data demonstrate a role for macrophages in promoting the accumulation of transitional AECs, possibly via the secretion of TGF-β and IL-1β, and suggest that K8 may promote the accumulation of transitional AECs and fibrosis by inducing the recruitment of macrophages.

Macrophages and K8^hi^ AECs ultimately promote fibrosis via activation of fibroblasts (1, 2). Therefore, we next considered whether fibroblasts and the fibrotic milieu may contribute to the accumulation of transitional AECs. In the bleomycin model, activated fibroblasts typically undergo apoptosis, and fibrosis resolves. To induce fibroblast persistence, we generated fibroblast-specific *Fas*-KO mice. *Col1a1CreERT2* *Fas^fl/fl^* mice were treated with bleomycin. *Fas* deficiency prevented fibroblast apoptosis, leading to the persistence of myofibroblasts and fibrosis until at least 9 weeks after bleomycin treatment (Figure 5C and Supplemental Figure 5C). In contrast to WT mice, in which transitional cells resolve, *Col1a1CreERT2* *Fas^fl/fl^* mice exhibited a persistence of transitional AECs for at least 9 weeks (Figure 5C and Supplemental Figure 5C). To confirm that *Col1a1CreERT2*-mediated recombination was specific for fibroblasts, we first examined *Col1a1* expression in the bleomycin model by single-cell RNA-Seq (scRNA-Seq) and found that epithelial, endothelial, and immune cells expressed much lower levels of *Col1a1* than did fibroblasts (Supplemental Figure 5D). Moreover, in *Col1a1CreERT2* *TdTomato* mice treated with the same bleomycin and tamoxifen regimen as the *Col1a1CreERT2* *Fas^fl/fl^* mice shown in Figure 5C, the epithelial, endothelial, and immune cells were lineage negative (Supplemental Figure 5E). Taken together, these data suggest that the persistence of activated fibroblasts and fibrosis was sufficient for the accumulation of transitional AECs.

Given the ability of fibroblasts to deposit collagen and increase lung stiffness, we next explored the role of stiffness and collagen in accumulation of the transitional state. We compared the differentiation of AECs cultured on a soft substrate, composed mainly of the native extracellular matrix protein laminin (Matrigel), with cells grown on a stiff substrate (plastic) coated with collagen. The collagen-coated stiff substrate, but not the soft laminin-based substrate, promoted the transitional state (Figure 5D). To determine whether collagen and/or a stiff substrate may be sufficient to maintain *Krt8^–/–^* AECs in the transitional state even though *Krt8^–/–^* AECs differentiated into AEC1s as stiffness and collagen (fibrosis) resolved in vivo (Figure 2G), we reexamined gene expression in *Krt8^+/+^* and *Krt8^–/–^* AECs, which were cultured in 2D on a stiff substrate coated with laminin (Figure 3A). As mentioned, the 2D culture system is widely used as an assay of AEC1 differentiation, although AEC2s differentiate into “AEC1-like” cells, which are similar but not transcriptionally identical to AEC1s (50, 75–79). We noted that most transitional state markers were not downregulated as AEC1 markers were upregulated, suggesting that bulk RNA-Seq may reflect a mixture of AEC1s and transitional cells. Indeed, immunostaining of AECs on day 7 of 2D culturing revealed some AEC1s with many cells persisting in the transitional state (Figure 5E and Supplemental Figure 5F). In this context, the failure of *Krt8^–/–^* AECs to downregulate transitional state markers and exhibit enhanced upregulation of AEC1 markers suggests that a stiff substrate without collagen was sufficient to maintain the transitional state in the absence of K8 (Figure 3B and Supplemental Figure 4B), i.e., to prevent the AEC1 differentiation observed in *Krt8^–/–^* cells in vivo.

Since fibrosis maintained the transitional state (Figure 5C), we next asked whether the enigmatic, nongradual AEC2-to-AEC1 differentiation through the transitional state observed in “nonfibrotic” mouse models may be driven by occult fibrosis. Close examination revealed that, even in the presumed nonfibrotic LPS and PNX models, there were small, peripheral foci of fibrosis (Figure 5F). Importantly, transitional cells were mainly detected in these areas of fibrosis and were rare in nonfibrotic areas of lung despite active regeneration (Figure 5G and Supplemental Figure 5, G and H). The apparent nongradual nature of AEC2-to-AEC1 differentiation, pausing in the discrete transitional state, that were observed in scRNA-Seq studies in the LPS and PNX models may be attributable to focal areas of fibrosis (50, 56). In contrast, in areas of regeneration without transitional cells or fibrosis, AEC2s may either pass through the transitional state too quickly to be captured on fixed tissue or may bypass the transitional state entirely. Our prior pseudotime analysis of scRNA-Seq of the LPS model of nonfibrotic regeneration suggested, by multiple methods, a trajectory that bypasses the transitional state (Supplemental Figure 5I) (50).

### Meta-analysis of murine scRNA-Seq data sets.

**Figure 6:**
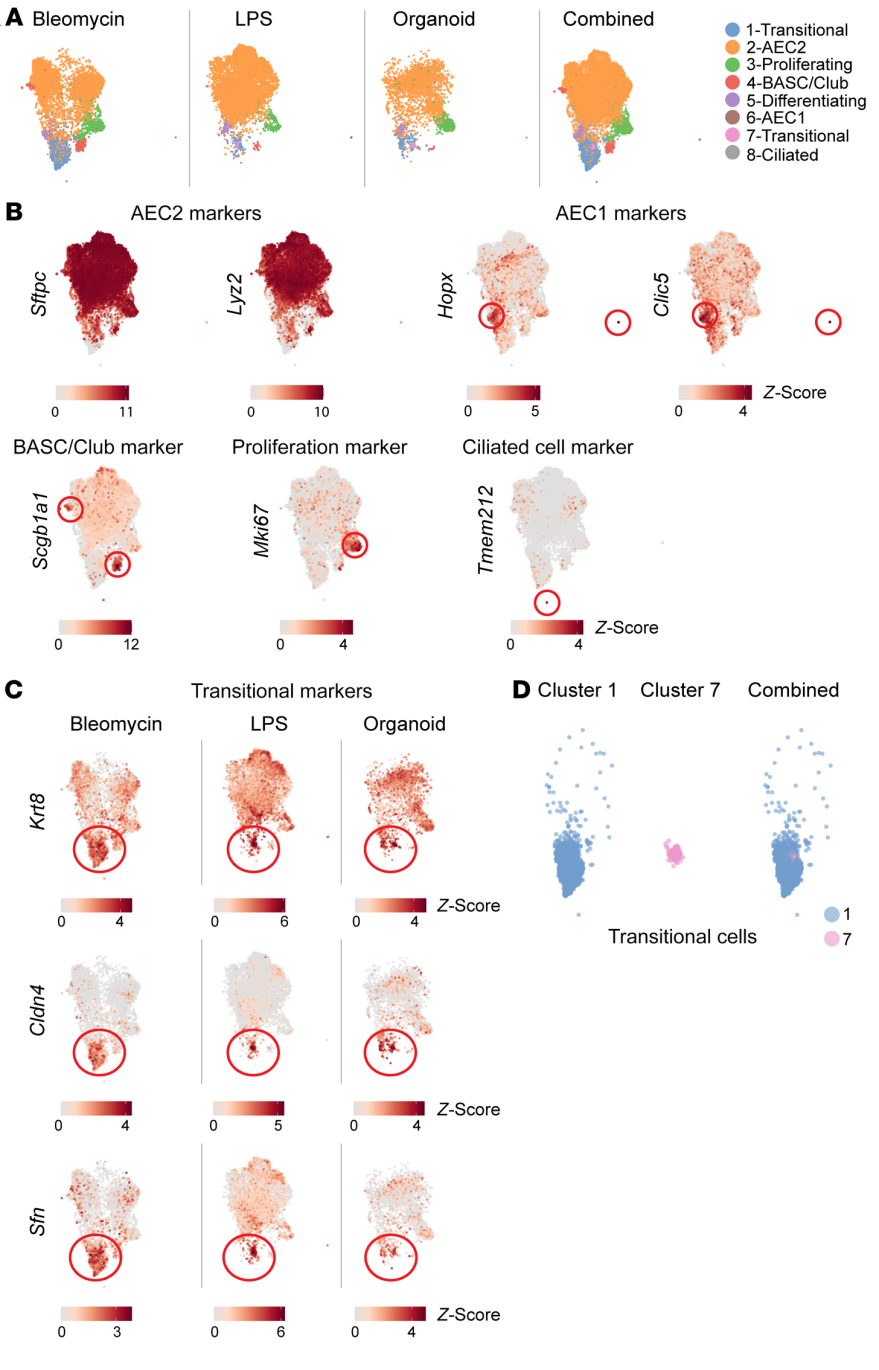
Meta-analysis of scRNA-Seq data sets from the bleomycin, LPS, and organoid models of alveolar regeneration. scRNA-Seq data sets from the bleomycin (54), LPS (50), and organoid (53) models were integrated and subjected to (**A**) unsupervised clustering. (**B** and **C**) Clusters were annotated on the basis of expression of canonical markers of AEC2s, AEC1s, BASCs, proliferating AEC2s, and transitional cells. Transitional cells from all 3 models coclustered. (**A** and **D**) Unsupervised clustering revealed 2 clusters of transitional cells.

The above data implicate a positive feedback loop between K8, transitional AECs, macrophage recruitment, and fibroblasts that drives and maintains fibrosis. Since the transcriptomes of transitional cells in mouse models and human IPF were similar (Supplemental Figure 1, A and B), a perplexing and clinically relevant question is why transitional cells differentiate into AEC1s in mouse models, with the resolution of fibrosis, whereas AEC1 differentiation is impaired, with progressive fibrosis, in human IPF. Although lineage-tracing studies demonstrated that some transitional cells ultimately differentiate into AEC1s in mice (52, 53, 62), pseudotime analysis of our LPS scRNA-Seq data set suggested that others may not (Supplemental Figure 5I) (50). Moreover, there are some differences in the transcriptomes of murine and human transitional cells (Supplemental Figure 1A,B). Finally, an emerging paradigm distinguishes a transiently senescent state with a beneficial role in physiologic regeneration from permanent senescence, which drives diseases of aging (26, 29, 31–33, 82). Therefore, we hypothesized that the transitional cell state identified in mouse models of regeneration may include 2 subsets: cells that exit the cell cycle and transiently assume a senescence-like phenotype in anticipation of AEC1 differentiation and cells that are permanently senescent and have lost the capacity for an AEC1 fate, the latter of which may more closely recapitulate the nonresolving transitional cells present in the IPF lung. To explore this, we integrated scRNA-Seq data sets from 3 mouse models: LPS, organoids, and bleomycin (50, 53, 54) (webtool available at https://github.com/kriemo/lung-regeneration-meta). We performed unbiased clustering. Clusters were annotated on the basis of expression of canonical markers (Figure 6, A–C) and were consistent with the identification in the original studies (Supplemental Figure 6A). In the LPS scRNA-Seq study, mature AEC1s were isolated from naive mice using an enzymatic digestion and centrifugation protocol designed to preserve fragile AEC1s (83) and an antibody cocktail specific for FACS of AEC1s. The yield was low. In both the original study (50) and in the integrated data set (Figure 6, A and B, and Supplemental Figure 6, A and B), these naive AEC1s clustered separately from most “differentiating” cells, which arose from lineage-labeled AEC2s and expressed lower levels of transitional markers and higher levels of AEC1 markers than did transitional cells (50). Interestingly, we identified minimal mature AEC1s in the bleomycin and organoid data set (Supplemental Figure 6B), with the cells identified as AEC1s clustering with the lineage-labeled differentiating cells rather than mature AEC1s. As expected, based on their conserved transcriptomes, transitional cells from all 3 models coclustered (Figure 6, A and C, and Supplemental Figure 6A).

### A highly senescent subset of mouse transitional AECs without the AEC1 fate.

**Figure 7:**
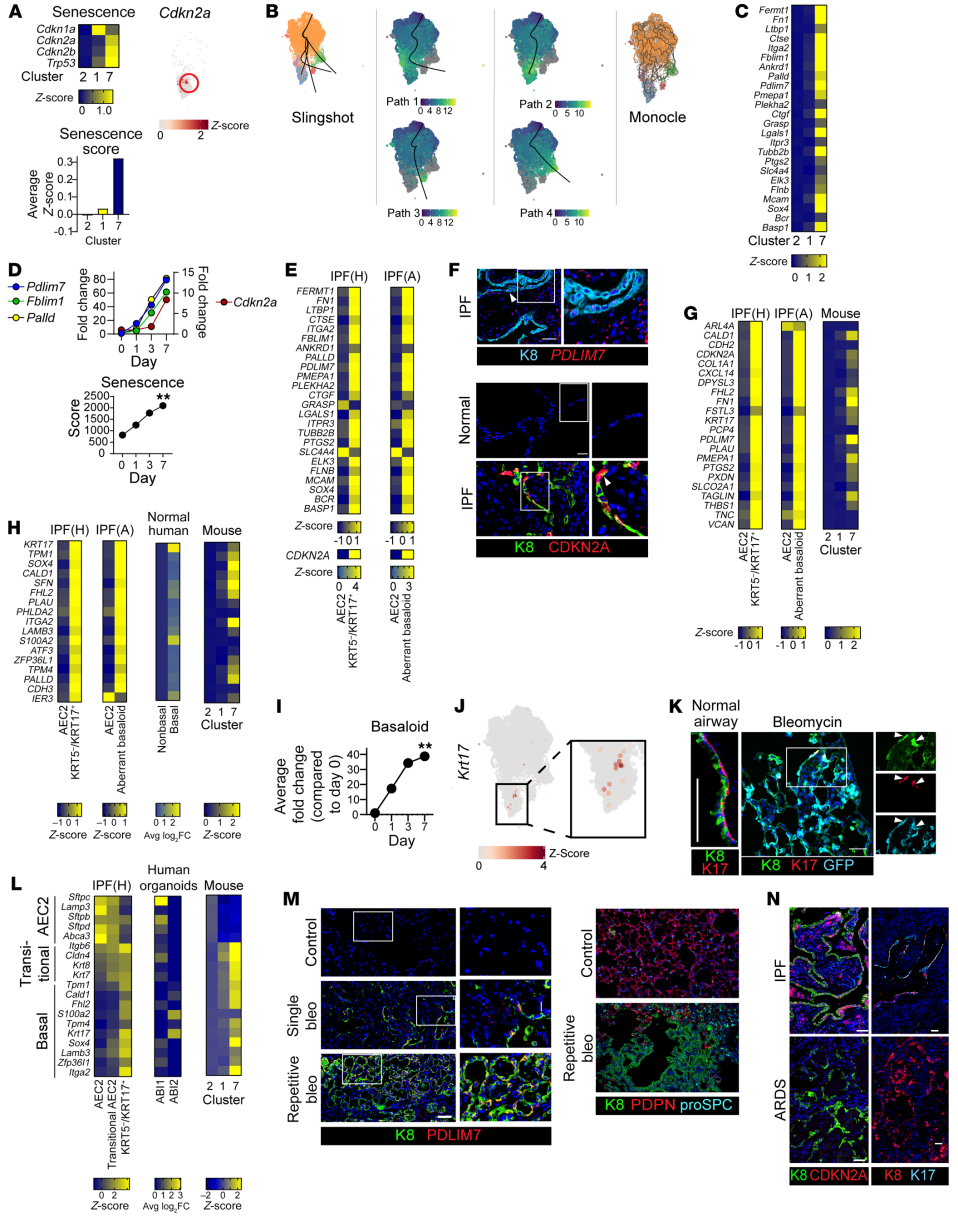
Murine transitional cells include a highly senescent, basaloid subset. Bleomycin, LPS, and organoid scRNA-Seq data sets were integrated. (**A**) Expression of senescence markers. p16 (*Cdkn2a*) was exclusively expressed in cluster 7. (**B**) Pseudotime analysis suggested cells in cluster 7 may not have an AEC1 fate. (**C**) Top DEGs in cluster 7. (**D**) Cultured primary murine AEC2s upregulated cluster 7 markers and p16 (*Cdkn2a*) and were highly senescent. Human IPF transitional cells expressed high levels of cluster 7 markers, as shown by scRNA-Seq (**E**) and immunostaining or FISH (**F**). (**G**) The top DEGs in the human IPF transitional (*KRT5^–^KRT17^+^)* state (58) were differentially expressed in murine cluster 7. (**H**) scRNA-Seq data sets from IPF (57, 58), normal human lung (86), or human organoids (60) were interrogated. Mature basal cell genes found among the top 100 DEGs of the human IPF transitional state were upregulated in cluster 7 of the murine AECs (**H** and **J**) and in cultured murine AECs (**I**). (**K**) K17 was occasionally expressed in lineage-labeled cells in bleomycin-treated *SftpcCreERT2* *mTmG* mice. (**L**) Compared with AEC2s, human “transitional AEC2s” from IPF (58), ABI1s from human organoids (60), and murine transitional cells in cluster 1 downregulated AEC2 markers and upregulated classic transitional state markers. *KRT5*^–^*KRT17^+^* AECs from IPF (58), ABI2s from human organoids (60), and murine transitional cells in cluster 7 upregulated basaloid genes. (**J** and **M**) Transitional cells expressing cluster 7 markers were rare in the single bleomycin model but common in the repetitive bleomycin model. (**N**) Transitional cells in human IPF but not ARDS expressed basaloid markers and p16/CDKN2A. (**D** and **I**) Data represent the mean (*n* = 3). **P < 0.01 compared with day 0, by 1-way ANOVA with post hoc Bonferroni’s test. (**D**) *P* < 0.05 for all genes at day 7 compared with day 0. Scale bars: 50 μm. Original magnification, ×20 (enlarged insets in **F**, **K**, **M**, and **N**). Arrowheads indicate transitional cells expressing a marker of interest. For immunostaining, *n* = 3 mice/group.

Transitional cells were found in 2 clusters, clusters 1 and 7 (Figure 6, A, C, and D). Both clusters contained cells from all 3 data sets (Supplemental Figure 7A). General markers of senescence, p15/*Cdkn2b*, p21/*Cdkn1a*, and p53, were expressed in both clusters. However, the cells in cluster 7 were highly senescent, as shown by a high composite senescence gene score and the exclusive expression of p16 (also known as *Cdkn2a*) (Figure 7A, Supplemental Figure 7B), a highly specific marker of permanent senescence (26, 27, 82). Cells in cluster 7 also exhibited highly activated TGF-β signaling (Supplemental Figure 7B). Cluster 7 arose later in the time course of the bleomycin model than did cluster 1 (Supplemental Figure 7C). Of note, the genes used to lineage trace the transitional cells in studies demonstrating that they have an AEC1 cell fate (52, 53, 62) were not specific to cluster 7 (Supplemental Figure 7D). Moreover, pseudotime analysis predicted that cluster 7 may represent an alternative terminal cell fate for transitional cells other than AEC1s (Path 2 in Slingshot, Endpoint 4 in Monocle) (Figure 7B, Supplemental Figure 5I, and Supplemental Figure 7E). These results are consistent with the existence of a murine transitional cell subset that is highly senescent and does not differentiate into AEC1s (cluster 7). Of note, pseudotime analysis also suggested a lineage trajectory through which AEC2s may bypass the transitional state, assuming the differentiating state, and ultimately the mature AEC1 state (Path 1 in Slingshot, Endpoint 1 in Monocle in Figure 7B), as previously suggested in the LPS study (Supplemental Figure 5I) (50).

To define a cluster 7 gene signature, we identified the top DEGs in cluster 7 (Figure 7C, Supplemental Figure 7F, and Supplemental Table 4) and focused on the genes that had the lowest levels of expression in AEC2s and AEC1s: *Fblim1*, *Palld*, and *Pdlim7*. Interestingly, by day 7 of 2D culturing, AECs had upregulated cluster 7 genes and were highly senescent (Figure 7D), suggesting that culturing AEC2s in 2D recapitulated not only the transitional state but a subset of murine transitional cells that may not have an AEC1 fate. Conversely, that many cultured AECs in the cluster 7 state did not differentiate into AEC1s (Figure 5E and Figure 7D) corroborates the pseudotime data (Figure 7B) suggesting that the cluster 7 state and the AEC1 state were divergent cell fates. Taken together with the lineage-tracing studies (52, 53, 62), these data suggest that there were 2 discrete populations of transitional cells, a population of cells in a transient senescence-like state that may ultimately differentiate into AEC1s, and another cell population marked by *Fblim1*, *Palld*, and *Pdlim7* that was highly senescent and persisted in the transitional state.

### A highly senescent, basaloid subset of mouse transitional AECs recapitulates IPF aberrant basaloid cells.

Since both murine cluster 7 cells and human IPF transitional cells appear not to differentiate into AEC1s and are highly senescent (p16^+^) (57, 58), we asked whether these cell types are analogous. Indeed, the cluster 7 signature was also exhibited by transitional AECs in IPF (Figure 7, E and F, and Supplemental Figure 7G), suggesting that they were analogous. Conversely, the most highly DEGs in the human transitional state were more highly expressed in murine cluster 7 than cluster 1 (Figure 7G). Human IPF transitional cells, unlike murine IPF transitional cells, have been referred to as “aberrant basaloid” or *KRT17*^+^*KRT5*^–^ because they express basal cell markers such as *KRT17* (57, 58). Moreover, human but not murine AEC2s have been shown to give rise to basal cells (60). This discrepancy has led to a concern that mice are not suitable models of the human AEC2 lineage and IPF. However, we found that *Krt17* was upregulated in murine transitional cells (Figure 1A). Moreover, many of the cluster 7 markers shown in Figure 7C — *LGALS1*, *ITGA2*, *PALLD*, *BASP1*, and *CTGF* — were basal cell genes (84). To confirm whether the murine cluster 7 cells had a basaloid phenotype, we first identified the genes that define the basaloid phenotype of the human aberrant basaloid or *KRT17*^+^*KRT5*^–^ cells. Of the top 100 DEGs of the human IPF transitional state, 17 were basal cell markers (Figure 7H). Of the 17 markers that comprise this basaloid signature, 16 were also upregulated in the murine cluster 7 state in vivo (Figure 7H and Supplemental Figure 7H) and in vitro (Figure 7I and Supplemental Figure 7I). Although cluster 7 was enriched for most of these markers, some of them, such as *Krt17*, were expressed only in a subset of the cells in cluster 7 (Figure 7J and Supplemental Figure 7F). However, rare K17^+^ cells were confirmed by immunostaining (Figure 7K and Supplemental Figure 7J). The basaloid cells in culture (Figure 7I and Supplemental Figure 7I) and in the LPS, organoid, and bleomycin models (Figure 7, H and K) can arise from AEC2s (or bronchoalveolar stem cells [BASCs]) based on *Sftpc* lineage tracing. We conclude that the murine cluster 7 state, or a subset thereof, recapitulated the basaloid phenotype of the human IPF aberrant basaloid cells. Of note, human but not murine aberrant basaloid cells express *TP63*, and neither murine cells nor human aberrant basaloid cells express *NGFR*, *KRT5*, *KRT14*, or *KRT15* (data not shown and refs. 57, 58, 60).

Deeper interrogation of the human IPF transitional cell state revealed that it was composed of 2 discrete cell states, referred to as alveolar basal intermediate 1 (ABI1) and ABI2 (60), or “transitional AEC2” and KRT17^+^KRT5^–^ (58), respectively. Here, we found that, similar to the human transitional AEC2s or ABI1s, the cluster 1 transitional cells from each of the mouse models downregulated AEC2 markers and upregulated classic transitional state markers such as *Krt8*, *Cldn4*, and *Itgb6*, whereas similar to the *KRT17*^+^*KRT5*^–^ AEC2s or ABI2s, the murine cluster 7 cells and the human IPF *KRT17*^+^*KRT5*^–^ cells upregulated basal cell genes (Figure 7L). Likewise, murine AEC2s cultured in 2D assumed the transitional (ABI1) state on day 1 of culturing (Figure 3A) and assumed the KRT5^–^KRT17^+^ ABI2 state by day 7 of culturing (Figure 7I and Supplemental Figure 7I). Thus, the murine cluster 7 state recapitulated the human aberrant basaloid or *KRT17*^+^*KRT5*^–^ or ABI2 state, and the murine cluster 1 transitional state recapitulated the human transitional AEC2 or ABI1 state. Moreover, pseudotime suggested that the human ABI1s or transitional AEC2s may be able to differentiate into AEC1s or assume the ABI2 or aberrant basaloid state (60). Taken together, these data suggest that murine and human transitional cells consisted of 2 subsets, one that was transient and may differentiate into AEC1s (henceforth referred to as “transitional” in both mice and humans), and another that was permanently senescent, basaloid, and failed to differentiate into AEC1s (henceforth referred to as “aberrant basaloid” in both mice and humans).

Since murine aberrant basaloid cells recapitulated the human aberrant basaloid/*KRT17*^+^*KRT5*^–^ state, which was associated with loss of an AEC1 fate and nonresolving fibrosis, we next asked whether a murine model of nonresolving fibrosis would be characterized by prevalent aberrant basaloid cells with a paucity of AEC1s. We treated mice with multiple doses of bleomycin and stained lung sections for the cluster 7 markers PDLIM7 and K17. Aberrant basaloid cells were more abundant and may have existed in a histologic pattern of “bronchiolization” (Figure 7M), recapitulating the architecture of human IPF. In contrast, aberrant basaloid cells were rare in the single bleomycin model, a model of resolving fibrosis (Figure 7, K and M, and Supplemental Figure 7C). Although IPF is characterized by nonresolving fibrosis, most patients with acute lung injury recover normal lung structure and function (64), similar to what is observed in mice. Therefore, we wondered whether regeneration after human acute lung injury is characterized by transitional but not aberrant basaloid cells. We stained lung tissue from patients during the first 14 days after acute lung injury. We observed no fibrosis (71), and there were transitional (K8^hi^) but not aberrant basaloid (p16^+^/CDKN2A, K17^+^) cells (Figure 7N and Supplemental Figure 7K). We speculate that, similar to mice, these cells resolve as patients recover normal lung structure and function. We conclude that in the single bleomycin model and human acute lung injury, AECs assumed the transitional state, a transient senescence-like state, and could retain an AEC1 fate, whereas in human IPF or after multiple doses of bleomycin in mice, cells evolved into the aberrant basaloid state of permanent senescence, failed to differentiate into AEC1s, and drove progressive fibrosis.

## Discussion

**Figure 8:**
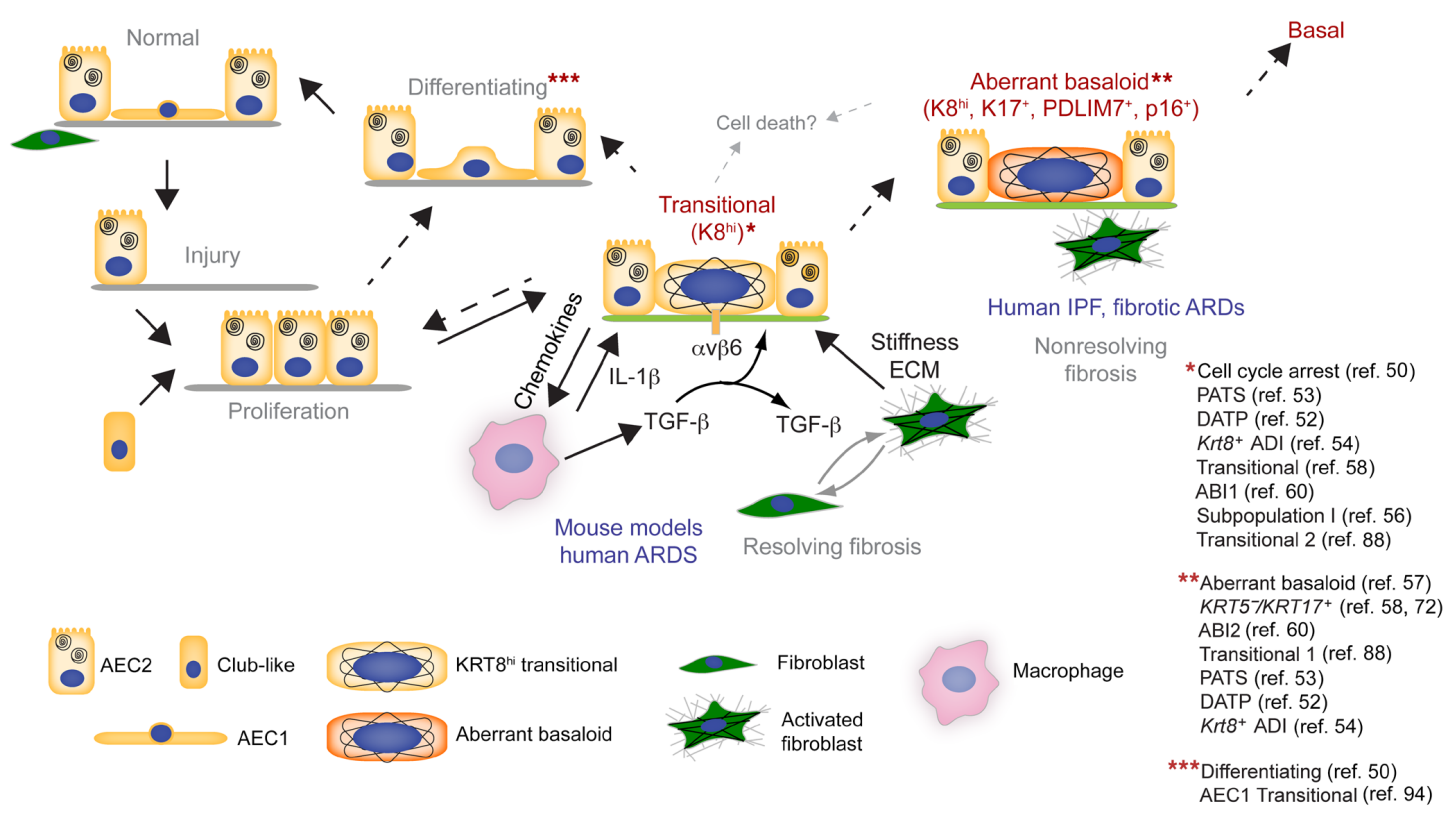
Regulation of epithelial transitional states in murine and human pulmonary fibrosis. Our current working construct is that after injury, alveolar progenitors assume the K8^hi^ transitional state characterized by the activation of multiple profibrotic processes: senescence, impaired proteostasis, DNA damage, cell death, integrin β6-dependent TGF-β activation, and macrophage chemokine expression. K8 promotes fibrosis by regulating expression of macrophage chemokines, which recruit profibrotic macrophages that further drive fibrosis. Fibroblasts are activated to contract and deposit matrix, stiffening the lung. Stiffness, as well as TGF-β, largely synthesized by macrophages and activated by transitional AEC integrin β6, and IL-1β, synthesized by macrophages, promote accumulation of the AEC transitional state at the expense of AEC1 differentiation. Taken together, our data suggest that crosstalk between K8^hi^ transitional AECs, profibrotic macrophages, and activated fibroblasts maintain each other in an activated state in the lung, establishing a positive feedback loop that drives fibrosis. In the absence of fibrosis, AEC2s may bypass the transitional state and differentiate into AEC1s (dotted line). In mouse models and in humans who recover from acute lung injury, this positive feedback loop is eventually broken, and transitional cells differentiate into AEC1s or perhaps die with resolution of macrophages and activated fibroblasts; in human IPF, the transitional cells further evolve into a permanently senescent, aberrant basaloid state instead of into AEC1s, driving a self-amplifying feedback loop that underlies the progressive and ultimately fatal clinical disease. Adapted from ref. 71 with permission.

Here, we confirmed that the keratin^hi^ transitional cells uniquely activated multiple profibrotic processes, thus unifying diverse, independent lines of investigation. Genetic variants in *KRT8* were associated with IPF (Figure 2A). *Krt8^–/–^* mice were protected from fibrosis and from accumulation of transitional cells at the expense of AEC1 differentiation (Figure 2). However, *Krt8^–/–^* AECs were not protected from transitional cell accumulation in vitro (Figure 3B), suggesting a role for cell-cell crosstalk. K8 was necessary for chemokine expression and macrophage recruitment (Figure 3E and Figure 4). In turn, macrophage recruitment promoted the accumulation of transitional AECs (Figure 5A), perhaps via IL-1β and TGF-β (Figure 5B). Fibroblast persistence was sufficient for transitional AEC accumulation (Figure 5C), as was substrate stiffness (Figure 3A and Figure 5, D and E). Based on these findings, our working construct is that K8 in AECs induced chemokine expression and the recruitment of profibrotic macrophages, which, together with the profibrotic transitional AECs, activated fibroblasts and promoted fibrosis. Macrophages and fibroblasts in turn promoted the accumulation of transitional AECs (Figure 8). However, in mouse models, but not human IPF, this positive feedback loop between K8^hi^ transitional AECs, macrophages, and fibroblasts resolved. Insight into the divergent outcomes may be provided by our discovery that murine and human transitional cells consist of 2 subsets: cells in a “transitional” state that is transient and, based on lineage tracing (52, 53, 62), can differentiate into AEC1s, and cells in an “aberrant basaloid” state that is senescent, basaloid, and may not have an AEC1 fate. In mouse models, most AECs assumed the transitional state, which transiently induced and was maintained by inflammation and fibrosis but ultimately resolved, in part by AEC1 differentiation, with resolution of fibrosis; in human IPF, many transitional cells evolved into the aberrant basaloid state instead the AEC1 fate, generating a self-amplifying feedback loop that drove progressive fibrosis (Figure 8).

Remarkably, a keratin^hi^, partially spread AEC state, thought to be in the process of AEC2 to AEC1 differentiation, was described in cultured cells and in the bleomycin model nearly 40 years ago (85). Since the characterization of the transitional AEC state by scRNA-Seq studies in mice and human IPF, keratins have been used as marker genes, since they are highly upregulated in the transitional state (51, 54, 58, 62) (although we found that *Krt8* was also expressed in mature AEC2s and AEC1s; Figure 6C and Supplemental Figure 1C). However, we show here for the first time to our knowledge that keratins play a functional role in fibrosis. K8 regulated the SASP, specifically chemokine expression, in transitional cells (Figure 3E and Figure 4A) and the recruitment of macrophages (Figure 4, B and C), which are strongly implicated in fibrosis (2, 19–21, 24, 34–42, 55). Although we focused on CCL2, the effect of K8 on macrophage recruitment and fibrosis is likely mediated by multiple chemokines (Figure 3E). Other cell types, including recruited macrophages, also produce chemokines, amplifying recruitment (25, 54). This role for K8 in chemokine expression in the context of pulmonary fibrosis contributes to a nascent literature on the nonmechanical functions of keratins, including in the regulation of inflammation (66, 69). Furthermore, that the SASP mediates the profibrotic effect of K8 is consistent with growing recognition of a pivotal role for the SASP in fibrogenesis (2, 11–15, 30).

The role of K8 in the accumulation of transitional AECs appeared to be non-cell-autonomous. K8 promoted the accumulation of transitional cells at the expense of AEC1 differentiation in vivo but not in vitro (Figure 2G, Figure 3B, and Supplemental Figure 4B), suggesting that K8 did not directly regulate cell differentiation. Instead, K8 promoted macrophage recruitment, and macrophages and fibroblasts in turn promoted the accumulation of transitional AECs. Macrophages produced IL-1β and TGF-β (Figure 5B) (40, 52, 81), which promoted the transitional state (50, 52). TGF-β was activated by transitional AEC integrin β6 (2, 17, 18, 50) (Figure 1E). That fibroblasts promoted the transitional state in vivo (Figure 5C) is consistent with a recent report that transitional AECs arise from inducible pluripotent stem cell–derived (iPSC-derived) human AEC2s only in the presence of mesenchymal cells (86). The effect of fibroblasts on transitional AEC accumulation may have been mediated by stiffness (Figure 5D), also consistent with older literature (87). Indeed, a stiff substrate was sufficient to rescue accumulation of the transitional state in *Krt8^–/–^* cells in the absence of macrophages and fibroblasts (Figure 3B, Figure 5E, and Supplemental Figure 4B). The role of macrophages and fibroblasts in transitional AEC accumulation explains the localization of transitional cells in peripheral areas of inflammation and fibrosis (Figure 5, F and G) and the nongradual cell fate trajectory observed with scRNA-Seq (50, 53, 54, 56). Our working construct is that K8 promotes the recruitment of profibrotic macrophages, which produce IL-1β and TGF-β. TGF-β, activated by transitional AEC integrin β6, activates fibroblasts, which generates stiffness. IL-1β, activated TGF-β, and stiffness in turn promote the accumulation of transitional AECs at the expense of AEC1 differentiation, establishing a positive feedback loop that drives and maintains fibrosis and transitional AECs (Figure 8).

The development of drugs to reverse fibrosis might be facilitated by understanding why fibrosis and transitional AECs resolve in mouse models but not in human IPF. Here, we provide insight into this question by distinguishing 2 subsets of transitional cells in both mice and humans: murine transitional cells, which are analogous to human ABI1 (60) or AEC2 transitional (58) cells, and a rare subset of murine transitional cells that transcriptionally recapitulate the human ABI2 (60), *KRT5^–^KRT17^+^* (58), or aberrant basaloid (57) phenotype. Marker genes that distinguish this subset from other murine transitional cells (*Fblim1*, *Palld*, and *Pdlim7*) were conserved in the human aberrant basaloid state (Figure 7, E and F, and Supplemental Figure 7G), and, conversely, marker genes that distinguish the human aberrant basaloid state, including basal cell genes, were conserved in this murine subset (Figure 7, G, H, K, and M). Consistent with these findings, *Krt8*^hi^*Krt17^–^* and *Krt8*^hi^*Krt17^+^* subsets of murine transitional cells were recently shown to arise from epithelial progenitors transplanted into bleomycin-treated mice (88).

Similar to the human cells, murine aberrant basaloid cells may not have an AEC1 fate, as suggested by pseudotime analysis (Figure 7B and Supplemental Figure 7E) and by their failure to differentiate into AEC1s in 2D culture and in the repetitive bleomycin model (Figure 5E and 7, D, I, and M). Human aberrant basaloid cells exist on a trajectory toward mature basal cells (60); whether murine aberrant basaloid cells have this potential is unknown. In contrast to aberrant basaloid cells, “transitional” AEC2s may differentiate into AEC1s or aberrant basaloid cells, as suggested by lineage tracing (52, 53, 62) and pseudotime analysis (Figure 7B, Supplemental Figure 5I, and Supplemental Figure 7E) (58, 60, 88). They may also revert to AEC2s (52). Finally, given their cell death gene signature (Figure 1D), apparent resolution in some but not all studies (Supplemental Figures 7, C and J), and the sheer alveolar surface area that would be required for all the abundant transitional cells to spread and differentiate into AEC1s, it is likely that some cells die. However, since pseudotime analysis is merely predictive and subject to caveats (89), it is imperative that lineage trajectories be clarified with rigorous experimentation.

Murine and human aberrant basaloid cells appeared to be senescent, as demonstrated by the expression of p16 (*Cdkn2a/CDKN2A*) (Figure 7, A and E, and Supplemental Figure 7B). Similarly, the murine aberrant basaloid cells that emerged in 2D culture were highly senescent (Figure 7D), consistent with their inability to be passaged. We speculate that, whereas the transitional cells may exit the cell cycle and assume a transient senescence-like state in anticipation of AEC1 differentiation, aberrant basaloid cells may be in a permanent state of senescence, driving unremitting and progressive fibrosis in human IPF. This notion is consistent with an emerging paradigm that transient senescence is a stress response that ultimately gives way to the regeneration of normal tissue, whereas permanent senescence underlies age-related diseases, (26, 29, 31–33, 82). Accordingly, during early regeneration after acute lung injury in humans, transitional cells existed in the transitional (K8^hi^), but not permanently senescent (p16^+^), basaloid (K17^+^) state (Figure 7N and Supplemental Figure 7K). Presumably, these patients, like most patients with acute respiratory distress syndrome (ARDS) (64), retain the capacity for normal lung regeneration (71).

Ultimately, the impact of this work will be realized by future mechanistic and clinical studies. Whether mechanisms other than chemokine expression mediate the role of K8 in fibrosis and whether keratins other than K8 regulate alveolar regeneration and fibrogenesis should be studied. Conditional-KO mice will be needed to confirm that K8 expressed by transitional cells arising from AEC2s and/or club-like cells (48, 54) regulates fibrosis. Whether the accumulation of transitional cells in fibrosis and the role of K8, macrophages, and fibroblasts in transitional cell accumulation are due to enhanced acquisition of the transitional state from progenitors versus impaired AEC1 differentiation from the transitional state will require rigorous experimentation, including with live imaging and stereology (43). In addition, whereas inhibition of the extrinsic pathway of apoptosis demonstrated that fibroblast persistence drove persistence of transitional AECs (Figure 5C), the intrinsic pathway of apoptosis can induce fibroblast apoptosis and fibrosis resolution (90). In fact, in so-called “type 2 cells,” which include fibroblasts (91, 92) (our unpublished observations), the intrinsic pathway played a role in Fas-mediated apoptosis. We speculate that activation of the intrinsic pathway would likely accelerate the resolution of transitional AECs. Minor transcriptomic differences between transitional states in diverse mouse models (Figures 6 and 7, Supplemental Figure 1, A and B, and Supplemental Figures 6 and 7) may be due to technical aspects but may reflect bonafide differences in the models that could be informative. We must uncover the mechanisms by which AECs that assume the transitional state transiently in anticipation of AEC1 differentiation acquire an aberrant basaloid rather than AEC1 fate, driving progressive fibrosis and, if possible, the mechanisms by which aberrant basaloid cells may be reverted to transitional cells with reversal of established fibrosis. The repetitive bleomycin and 2D AEC culture models are well suited for such investigation: aberrant basaloid cells were prevalent in the repetitive bleomycin model (Figure 7M), consistent with its nonresolving nature, and we report here that the enigmatic AEC1-like cells that arose in 2D culture were a mix of mature AEC1s and transitional/aberrant basaloid cells (Figure 3A, Figure 5E, and Figure 7, D and I). Aged mice, which also develop nonresolving fibrosis (93), may be useful. It also remains to be determined whether the transitional state is an obligate intermediate of AEC2-to-AEC1 differentiation. Pseudotime analysis (Figure 7B, Supplemental Figure 5I, and Supplemental Figure 7E) (50, 94) suggests that AECs may bypass the transitional state. Moreover, the localization of transitional cells to small, peripheral foci of fibrosis or inflammation in what are considered models of physiologic regeneration suggests that they may arise only in those settings (Figure 5, F and G, and Supplemental Figure 5G), although transitional AECs arise during development and appear to have an AEC1 fate (95). Improved methods to isolate AEC1s are needed, since we discovered that apparent AEC1s are probably progenitors in the process of differentiation, with loss of mature AEC1s during tissue digestion (Supplemental Figure 6B).

In summary, we demonstrate here that a keratin, K8, promoted fibrosis and accumulation of transitional cells at the expense of AEC1 differentiation. K8 regulated chemokine expression and the recruitment of macrophages, which, together with transitional epithelial cells, activated fibroblasts. Macrophages and activated fibroblasts in turn promoted the accumulation of transitional AECs. This positive feedback loop ultimately resolved in mice, with AEC1 differentiation and the resolution of fibrosis. However, in human IPF, transitional AECs evolved into a bonafide senescent, aberrant basaloid state that existed in a self-amplifying feedback loop of epithelium-macrophage-fibroblast crosstalk, in which fibrosis begat further fibrogenesis, driving the progressive and ultimately fatal clinical disease. Modulation of K8 or progression from the transitional to the aberrant basaloid state may be effective therapeutic strategies for pulmonary fibrosis.

## Methods

Details on the methods are provided in the Supplemental Methods.

### Data availability.

RNA-Seq data were deposited in the NCBI’s Gene Expression Omnibus (GEO) database (GEO GSE223302). Values for all data points in graphs can be found in the Supplemental Supporting Data Values file.

## Author contributions

FW and RLZ designed research studies. FW, CT, MD, KF, NP, NK, JN, BMV, JAW, EC, EFR, and XZ conducted experiments. FW, CT, MD, KAR, AL, EC, DWHR, EFR, DMT, XZ, BBM, PAC, MBO, and RLZ analyzed data. DMT and MBO provided reagents. RLZ wrote the manuscript. CT, KAR, JN, BMV, EC, DWHR, XZ, DMT, BBM, PAC, and MBO revised the manuscript.

## Supplementary Material

Supplemental data

Supplemental table 1

Supplemental table 2

Supplemental table 3

Supplemental table 4

Supporting data values

## Version 1 09/28/2023

In-Press Preview

## Version 2 11/15/2023

Electronic publication
