# Supplementary material for: Regulation of epithelial transitional states in murine and human pulmonary fibrosis
Source: J Clin Invest. 2023 Nov 15;133(22):e165612. doi: 10.1172/JCI165612 (PMC10645382; doi:10.1172/JCI165612)
Supplement: Supplemental data [file jci-133-165612-s123.pdf]

Methods

Human IPF and ARDS Tissue

Normal lungs from deceased individuals rejected for lung transplantation were obtained from Gift of Life Michigan. Lung tissue was also obtained from the autopsies of patients who died of acute respiratory distress syndrome (ARDS) within two weeks of symptom onset at the University of Michigan Hospital. Idiopathic Pulmonary Fibrosis (IPF) lung was explanted tissue from IPF patients who underwent lung transplantation at the University of Michigan Hospital.

Mouse Models

Krt8$^{-/-}$ mice (1) were obtained from Dr. Diana Toivola (Åbo Akademi University, Turku, Finland). *SftpcCreERT2* mice (2) were obtained from Dr. Harold Chapman. *C57BL/6* (000664), *Rosa26-mTmG* (abbreviated *mTmG,* 007576), *Rosa26-tdTomato (*abbreviated *tdTomato*, 007909), *Col1a1CreERT2* (016241), *Ccr2*$^{-/-}$ (004999) and *Fas*$^{f/f}$ (007895) mice were obtained from Jackson Laboratories. Alternatively, *B6129F2-Cmkbr2*^{tm1Kuz} (*Ccr2*$^{-/-}$) mice were obtained from Dr. William Kuziel (3) and backcrossed to *C57BL/6* for more than 10 generations at the University of Michigan. *SftpcCreERT2;mTmG* mice were administered 0.25 mg/g body weight tamoxifen (Toronto Research Chemicals, T0060000) in corn oil (Sigma C8267) i.p. every other day for 3 doses or fed tamoxifen chow (Envigo TD.130859) for 2 weeks starting at age 6 weeks. Mice were treated via intratracheal instillation or oropharyngeal aspiration with 40 µg LPS (List Biological Laboratories *E. coli* 0111:B4) once or 0.75-1.5 U/kg bleomycin (Meitheal Pharmaceuticals NDC 71288-106-10 or Fresenius Kabi C103610) once and euthanized at the indicated time points or 1 U/kg bleomycin every other week for 3 doses and euthanized at 24 w after the last dose of bleomycin (4). Littermate *Krt8*$^{+/+}$ and *Krt8*$^{-/-}$males were used. *Col1a1CreERT2;Fas*$^{f/f}$ or *Col1a1CreERT2;tdTomato* mice were administered 6 doses of 0.25 mg/g body weight tamoxifen or corn oil i.p. between days 4 and 21 after bleomycin. *Col1a1CreERT2;Fas*$^{f/f}$ mice were euthanized at 9 w after bleomycin (5). *Col1a1CreERT2;tdTomato* mice were euthanized at the indicated time points. Alternatively, mice were subjected to pneumonectomy as previously described (6) and accessory lobes were harvested. Bronchoalveolar lavage (BAL) was performed as previously described (7). BAL cytospins were stained with Kwik-Diff (Epredia 9990700). IgM (Bethyl Laboratories E99-101) and CCL2 (R&D Systems DY479-05) ELISAs were performed on BAL. For CCL2 ELISA on the *Krt8*$^{-/-}$ mice, BAL concentrations were normalized to the *Krt8*$^{+/+}$ mouse in each litter. Lungs were either homogenized in 1 ml cold PBS or fixed with 4% paraformaldehyde in 0.5% low melting temperature agar, processed, and embedded in paraffin or frozen in OCT. Hydroxyproline assay was performed on the lung homogenate from the right upper lobe for the time course and from all lung tissue for the *Krt8*$^{+/+}$ vs. *Krt8*$^{-/-}$ experiment. 500 µl of homogenate was transferred to glass vials and incubated with 500 µl of 12N hydrochloric acid (Sigma H1758) in an oven at 120˚C for 8 hours. Serial dilutions of

cis-4 hydroxy-L-proline standard (Sigma H1637) were utilized to generate the standard curve. In a flat-bottom 96 well plate, 5 µl of each sample was incubated with 5 µl of citrate/acetate buffer (5 g citric acid, Sigma C1909; 1.2 ml glacial acetic acid, Sigma A6283; 7.24 g sodium acetate, 3.4 g sodium hydroxide dissolved in 100 ml sterile water) and 100 µl of chloramine T solution (0.282 g chloramine T, Sigma C9887; 2 ml N-propanol, Sigma 402893; 16 ml citrate/acetate buffer, 2 ml sterile water) for 20 min at RT. 100 µl of Ehrlich's solution (2.5 g 4-DMAB, Sigma D2004; 9.3 µl N-propanol, Sigma 402893; 3.9 ml 70% perchloric acid, Sigma 244252) was added and incubated at 65˚C for 20 min. Plate was read at 550nm on a Synergy H1 plate reader (BioTek).

Tissue Staining

Fixed lung sections were immunostained as previously described (8, 9). Frozen sections were fixed in 1:1 ethanol:acetone at -20°C for 10 m. Primary antibodies used were: K8 (University of Iowa Developmental Studies Hybridoma Bank (DSHB) TROMA-I), αSMA (Sigma-Aldrich, C6198), CLDN4 (Invitrogen 364800), GFP (Abcam ab13970), PDPN (University of Iowa DSHB 8.1.1), SFN (Sigma PLA0201), K7 (Abcam ab181598), 4668 anti-K18 peptide antibody that recognizes the peptide RPVSSAASVYAGA [gift of M. Bishr Omary, Robert Wood Johnson Medical School, New Brunswick, NJ, USA (10, 11)], proSPC (Millipore, AB3786), tomato lectin (Vector Laboratories FL-1171), CD45 (Novus Biologicals NB100-77417), acetylated tubulin (Sigma 6-11B-1), CCSP (Santa Cruz Biotechnology sc-365992 or Seven Hills WRAB-3950), CDKN1A (Millipore, ZRB1141), K17 (Abcam ab53707), ϒH2AX (Novus Biologicals NB100-74435), CD68 (Abcam ab125212), CD206 (Bio-Rad MCA2235GA), CDKN2A (Abcam ab108349), PDLIM7 (Novus Biologicals NBP1-84841), HSPB1/HSP27 (Abcam ab5579), PDGFRa (Cell Signaling 3174), EMCN (R&D Systems AF4666, thrombomodulin (R&D Systems AF3894), E-cadherin BD Biosciences 610181), and Ki67 (Cell Signaling 9129). Secondary antibodies used were: Alexa Fluor 488–conjugated anti-rabbit (Invitrogen A11034 or Jackson ImmunoResearch Laboratories 711-545-152), Cy3-conjugated anti-chicken (Jackson ImmunoResearch Laboratories 703-165-155), Alexa Fluor 647–conjugated anti-hamster (Jackson ImmunoResearch Laboratories 107-605-142), FITC-conjugated anti-rat (Jackson ImmunoResearch Laboratories 712-095-153), Cy3-conjugated anti-rat (Jackson ImmunoResearch Laboratories 712-165-153), Cy5-conjugated anti-rat (Jackson ImmunoResearch Laboratories 712-175-153), FITC-conjugated anti-rabbit (Jackson ImmunoResearch Laboratories 711-095-152), Cy3-conjugated anti-rabbit (Jackson ImmunoResearch Laboratories 711-165-152), Cy5-conjugated anti-rabbit (Jackson ImmunoResearch Laboratories 711-175-152), FITC-conjugated anti-chicken (Jackson ImmunoResearch Laboratories 703-096-155), FITC-conjugated anti-Syrian hamster (Jackson ImmunoResearch Laboratories 107-095-142), Cy5-conjugated anti-mouse (Jackson ImmunoResearch Laboratories 715-175-151), Cy3-conjugated anti-mouse (Jackson ImmunoResearch Laboratories 715-165-151).

In situ hybridization (ISH) was performed using the RNAScope manual fluorescent multiplex kit V2 (ACDBio 323100) with the *Itgb6* probe (ACDbio 312501) according to the manufacturer's instructions, followed by immunostaining as described (8, 9). For H&E staining, slides were stained with Harris hematoxylin (ThermoFisher Scientific 842), differentiated with Clarifier (ThermoFisher Scientific 7401), blued with bluing reagent (ThermoFisher Scientific 7301), stained with eosin Y, alcoholic (ThermoFisher Scientific 832), then dehydrated and cleared through graded alcohols and xylene. For picrosirius red staining, slides were treated with 0.2% Phosphomolybdic Acid (Rowley Biochemical Inc. F-357-1) for 3 min, transferred to 0.1% Sirius Red saturated in picric acid (Rowley Biochemical Inc. F-357-2) for 90 min, then transferred to 0.01N Hydrochloric Acid for 3 min. For Masson's Trichrome staining, slides were mordanted in Bouin's fixative (Rowley Biochemical Inc. F-367-1) for 1 hour at 56°C. After washing, slides were placed in Biebrich scarlet-acid fuchsin (Rowley Biochemical Inc. F-367-3) for 15 min at RT, phosphotungstic-phosphomolybdic acid (Rowley Biochemical Inc. F-367-4) for 15 min at RT, and aniline blue stain (Rowley Biochemical Inc. F-367-5) for 8 min at RT. Images were acquired using a BX53 microscope (Olympus), Airyscan LSM 880 (Zeiss), Stellaris 5 (Leica), A1 confocal (Nikon), or Aperio AT2 DX (Leica) microscope. The number of transitional cells was determined by manually counting of CLDN4+ cells for the *Krt8*$^{-/-}$ experiment. For the wildtype time course and *Col1a1CreERT2;Fas*$^{f/f}$ and *Ccr2*$^{-/-}$ experiments, QuPath software (https://qupath.github.io/) was used to quantify cell number (which may be an overestimate due to spread cells being counted as multiple cells) and cell area. The number of *Itgb6*+ cells was determined by manually counting sections from bleomycin-treated lung immunostained for K8 and CLDN4 followed by FISH for *Itgb6* where indicated.

scRNAseq

UMI count matrices from the scRNAseq datasets from the bleomycin (12), organoid (13), and pneumonectomy (14, 15) mouse models and human IPF (16, 17) were downloaded from the NCBI Gene Expression Omnibus. The LPS scRNAseq dataset was obtained as previously described (8). For the heatmaps shown in Figure 1 and Supplemental Figure 1, the "cell cycle arrest" (18), "*Krt8*+ ADI" (12), PATS1 (13), Day 7 "subpopulation I" from wildtype mice (14) "*KRT5- KRT17+*" (17), and "aberrant basaloid" cells (16) were considered to be transitional cells. For the pneumonectomy dataset, naïve AEC1s from a similar study (15) were used. Z scores were calculated relative to all AECs cells in each study. For the bleomycin study, the epithelial only dataset was used, except for the *Tgfb1* and *Il1b* heatmaps, for which the whole lung dataset was used (12). In all figures, the IPF dataset published by Adams and colleagues (16) is labeled as "IPF(A)", and the IPF dataset published by Habermann and colleagues (17) is labeled as "IPF(H)". Pathway activation scores for senescence, TGFβ, impaired proteostasis, DNA damage, and apoptosis were calculated as the average Z-score of the canonical genes for each pathway listed in Supplemental Table 2, as has been done previously (19, 20). Heatmaps of orthologous genes were generated using GraphPad Prism software (v9). The top 400 differentially expressed genes

(DEGs) in the bleomycin transitional (*Krt8*+ ADI) cell state compared to other epithelial cells ranked by average log [Fold Change] (12) and the top 400 genes in the LPS transitional (cell cycle arrest) cell state compared to other epithelial cells ranked by average log [Fold Change] (8) were subjected to KEGG pathway analysis (https://maayanlab.cloud/Enrichr/) (21-23). The LPS (8), bleomycin AECs from epithelial only time course (12), and organoids (13) scRNAseq datasets were integrated with scVI (v 0.19.0), using 3000 highly variable genes, 2 hidden layers and 20 latent layers (24). Data processing for mouse datasets was performed using Bioconductor scRNA-seq analysis packages, scater (1.26.1), scran (1.26.2) and scuttle (1.8.4) (25, 26). Seurat (4.3.0) was used for the analysis of human datasets (27). Trajectory analysis was performed using Monocle3 1.3.1 (28) and Slingshot 2.6.0 (29). To identify clusters a SNN graph was built using 20 scVI dimensions and 10 nearest neighbors, and clustered with the igraph implementation of the Walktrap community finding algorithm (30). Differentially expressed genes were identified using findMarkers from scran, with pval.type = “some”, requiring a min.prop of 1/3. To identify a panel of marker genes specific for Cluster 7, the top 100 DEGs by fold change (Cohen) in Cluster 7 compared to Cluster 1 were ranked in ascending order of the fold change in the percent of cells in which the gene was detected, with genes expressed in <20% of Cluster 7 cells excluded. To identify a panel of marker genes specific for the human transitional state, the top 100 DEGs by fold change in the *KRT5-/KRT17+* state (17) were ranked in ascending order of the fold change in the percent of cells in which the gene was detected. To determine which of the human transitional state markers are basal cell genes, i.e., the markers that define the “basaloid” phenotype of the human aberrant basaloid or *KRT5-/KRT17+* cells, we cross-referenced the top 100 DEGs in the human transitional state (17) with the DEGs of basal cells using a scRNAseq database of normal human lung (31). Genes that were more highly expressed in AEC1s or AEC2s than in basal cells were excluded (16). For all comparisons between murine and human studies, genes that are not expressed in both species or without matching gene symbol identifiers were excluded.

GWAS

Using summary statistics of the genome-wide association meta-analysis on IPF from the International IPF Genetics Consortium (32), we performed a nested candidate gene study on the keratins differentially expressed in the transitional state in multiple mouse and human studies (5 genes) (8, 12-14, 16, 17). For each gene in these lists, we extracted their genomic positions using the transcripts of curated and predicted annotations provided by RefSeq adding +/- 5 kb buffer (https://www.ncbi.nlm.nih.gov/books/NBK21091/table/ch18.T.refseq_status_codes/?report=objectonly). Next, the SNPs of the IPF GWAS summary statistics that overlapped with these regions were extracted. Multiple-testing (Bonferroni) correction was applied, and the association of a candidate gene with the disease was considered statistically significant if a SNP within its genomic region had a p < (0.05 / number of SNPs overlapping with genes in candidate gene list). A candidate gene was considered nominally significant if a SNP within its genomic region had a p < (0.05 / number of SNPs

per gene). The full gene lists and the summary statistics of all overlapping SNPs are provided in Supplemental Table 1. Regional association plots were generated using Locuszoom (https://my.locuszoom.org/) (33). The combined annotation dependent depletion (CADD) (34) and Regulatory Mendelian Mutation (REMM) (35) scores were calculated.

Murine AEC2 Isolation and Culture

Mouse lung was harvested as previously described (36). Briefly, mice were anesthetized with inhaled isoflurane, exsanguinated, and perfused with PBS via the right atrium. 3 ml of dispase (Corning #354235) diluted 1:10 in HBSS-- was instilled into the trachea immediately followed by 0.5 ml of low-melting agarose. The lungs and heart were removed and incubated in 3 ml of dispase solution for 30 min at RT. Lung tissue was minced with a razor blade, then passed through 100 µm and 40 µm cell strainers. Cells were resuspended in PBS with 2% fetal bovine serum and 1 mM EDTA at a concentration of 10^8 cells/ml. Cells were incubated with rat serum (50 µl per 1 ml sample) and Fc block (BD Pharmingen 553142, 30 µg/ml) for 5 min, followed by incubation with biotin anti-mouse CD45 (BD Pharmingen 553078, 0.5 mg/ml), biotin anti-mouse CD31 (Biolegend 102404, 0.5 mg/ml), and biotin anti-mouse Ter119 (Biolegend 116203, 0.5 mg/ml) antibodies for 10 min at RT. Samples were then incubated with EasySep Mouse Streptavidin RapidSpheres (Stemcell Technologies 50001, 125µL per 1 ml of sample) for 2.5 min at RT and separated using an EasySep magnet (Stemcell Technologies 18000), according to the manufacturer's instructions. The flow-through solution was incubated with PE anti-mouse CD326/EpCAM antibody (Biolegend 118205, 0.2mg/ml) for 5 min at RT, followed by EasySep PE selection cocktail (Stemcell Technologies 18151, 100 µl per 1 ml of sample) for 3 min at RT. Cells were incubated again with EasySep Releasable Rapidspheres (Stemcell Technologies 50201, 50µl per 1 ml of sample) and an EasySep magnet, followed by the addition of Release buffer (Stemcell Technologies 20165) to separate the beads from cells. Cytospins were fixed with 4% paraformaldehyde. For culture, cells were resuspended in media [500 ml DMEM/F12, 1.25 g bovine serum albumin (BD Biosciences 354331), 5 ml 1M HEPES buffer (Corning MT25060CIS), 1% MEM non-essential amino acids (Sigma M7145), penicillin/streptomycin (Gibco 15140122), 250 µl 10mg/ml insulin-transferrin-sodium selenite solution (Roche 11074547001)], plated at a density of 0.5×10^6 cells per well on 1.1 cm^2 transwell inserts (Corning 3401) that had been preincubated with rat laminin-5 (Millipore CC145) for 1-3 hours. Cells were incubated at 37ºC with 5% CO_2. For RNAseq, AEC2 isolations were performed on 3 separate dates and cultured (3 biological replicates). RNA was isolated from cells using the RNeasy Micro kit (Qiagen 74004) according to the manufacturer's instructions. In other experiments, cells were fixed in 4% paraformaldehyde. Fixed cells and cytospins were permeabilized with 0.1% Triton-X in PBS for 5 min, blocked with 10% donkey serum in PBS-T for 30 min, and stained with antibodies against proSPC (Millipore AB3786), PDPN (University of Iowa DSHB 8.1.1), and K8 (University of Iowa DSHB TROMA-I) overnight

followed by secondary antibodies listed above for 1 hour and counterstained with DAPI (Thermo Scientific 62248) for 10 min. Slides were mounted with Prolong Gold (Invitrogen P36970). Purity of AEC2 isolation was ~90%.

Bulk RNAseq

Bulk RNAseq was performed by the University of Michigan Advanced Genomics Core. RNA quality was assessed using the Agilent Tapestation method, and all samples had an RNA integrity number (RIN) > 8.8. Poly-A enrichment was used to select mRNA for library preparation and the samples were sequenced on the NovaSeq S4 300 cycle. The reads were trimmed using Cutadapt v2.3. FastQC v0.11.8 was used to ensure the quality of data and Fastq Screen v was used to screen for various types of contamination. Reads were mapped to the reference genome GRCm38 (ENSEMBL) using STAR v2.7.8a and assigned count estimates to genes with RSEM v1.3.3. Alignment options followed ENCODE standards for RNAseq. Analysis was performed by the Bioinformatics Core of the University of Michigan Medical School. Briefly, data were pre-filtered to remove genes with 0 counts in all samples. Differential gene expression analysis was performed using DESeq2 with a negative binomial generalized linear model (thresholds: linear fold change >1.5 or <-1.5, Benjamini-Hochberg FDR (Padj) <0.05). Raw counts were log2 transformed and normalized by library size using DEseq2. Average $\log_2$FoldChange (FC) between groups were calculated by selecting desired comparisons in R. Individual $\log_2$FC was calculated manually by subtracting the $\log_2$ transformed and normalized count value of the reference group from the value of group of interest. For example, Log_2FC [Sample1/Sample2] = $\log_2$[Sample 1]-$\log_2$[Sample 2], where Sample 2 is the reference sample and Sample 1 is the sample of interest. Then, the fold change of Sample 2 compared to Sample 1 = $2^{\log_2 FC}$. AEC2, transitional, and AEC1 marker composites were calculated as the average fold change and pathway activation scores for senescence, TGFβ, impaired proteostasis, DNA damage, and apoptosis were calculated as the average normalized expression of the canonical marker genes for each cell type listed in Supplemental Table 2, as has been done previously (19, 20). PCA plot was generated using variations of DESeq2 plotting functions and other packages with R version 4.1.1. Whole genome analysis of AEC2s cultured on Matrigel or collagen-coated plastic was interrogated (37).

MLE-12

Lentivirus containing one of two distinct *Krt8* shRNA constructs, #1: TRCN0000325589 or #2: TRCN0000091874, or a scrambled shRNA construct cloned into the pLKO.1 vector (Sigma) was prepared by the University of Michigan Vector Core. MLE-12 cells (ATCC) were transduced with lentivirus (MOI 2.5) using 10μg/m polybrene (Sigma TR-1003-G). Cells

were cultured with 4 µg/ml puromycin (Sigma P8833) for 3 days after transduction. Cells were stimulated with 100 ng/ml tunicamycin (Cell Signaling 12819S) for 24 hours. RNA was isolated using the RNeasy Micro Kit (Qiagen 74004).

qPCR

RNA was reverse transcribed into cDNA using the Quantitect Reverse Transcription Kit (Qiagen 205311) according to the manufacturer's instructions. qPCR was performed using primers for m*Il1b* (Mm.PT.58.41616450) using iQ SYBR Green Supermix (Bio-Rad 1708880) on the StepOnePlus Real-Time PCR System (Applied Biosystems). Relative mRNA expression was calculated using the $2^{-\Delta\Delta Ct}$ method.

Statistics

Statistical analysis was performed by unpaired or paired 2-tailed t test or one-way or two-way ANOVA. For RNAseq, p-values were corrected for Benjamini-Hochberg FDR. In other experiments with multiple t testing, p-values were corrected by Bonferroni's or Tukey's or Sidak's post hoc analysis. For human scRNAseq datasets, differential gene expression was performed using Wilcoxon Rank-Sum Tests with Bonferroni corrections in Seurat. For mouse scRNAseq datasets, differential gene expression was performed with the findMarkers method from scran, using Welch T-tests, blocking based on the dataset each cell derived from, and adjusted using Benjamini-Hochberg method. Data are represented as mean or mean ± SEM or SD. $p < 0.05$ was considered statistically significant.

Study Approval

The use of human tissue was approved by the University of Michigan Institutional Review Board, Ann Arbor, Michigan, USA. For lungs explanted from IPF patients, written informed consent was obtained. For autopsy specimens from ARDS patients and normal cadaveric lungs rejected for transplantation, written informed consent was not obtained because subjects were deceased and deidentified. Animal protocols were approved by the University of Michigan Institutional Animal Care Use Committee, Ann Arbor, Michigan, USA and the State Provincial Office of South Finland, Helsinki, Finland (protocol 3956/04.10.07/2016).

Data Availability

RNAseq data has been deposited at the NCBI Gene Expression Omnibus (GEO) database, record # GSE223302. Other data is available in the supporting data values excel file or from the corresponding author upon request.

References

1. Baribault H, Penner J, Iozzo RV, and Wilson-Heiner M. Colorectal hyperplasia and inflammation in keratin 8-deficient FVB/N mice. *Genes Dev.* 1994;8(24):2964-73.
2. Chapman HA, Li X, Alexander JP, Brumwell A, Lorizio W, Tan K, et al. Integrin alpha6beta4 identifies an adult distal lung epithelial population with regenerative potential in mice. *J Clin Invest.* 2011;121(7):2855-62.
3. Kuziel WA, Morgan SJ, Dawson TC, Griffin S, Smithies O, Ley K, et al. Severe reduction in leukocyte adhesion and monocyte extravasation in mice deficient in CC chemokine receptor 2. *Proc Natl Acad Sci U S A.* 1997;94(22):12053-8.
4. Redente EF, Black BP, Backos DS, Bahadur AN, Humphries SM, Lynch DA, et al. Persistent, Progressive Pulmonary Fibrosis and Epithelial Remodeling in Mice. *Am J Respir Cell Mol Biol.* 2021.
5. Redente EF, Chakraborty S, Sajuthi S, Black BP, Edelman BL, Seibold MA, et al. Loss of Fas signaling in fibroblasts impairs homeostatic fibrosis resolution and promotes persistent pulmonary fibrosis. *JCI Insight.* 2020;6(1).
6. Liu S, Cimprich J, and Varisco BM. Mouse pneumonectomy model of compensatory lung growth. *J Vis Exp.* 2014(94).
7. Zemans RL, Briones N, Campbell M, McClendon J, Young SK, Suzuki T, et al. Neutrophil transmigration triggers repair of the lung epithelium via beta-catenin signaling. *Proc Natl Acad Sci U S A.* 2011;108(38):15990-5.
8. Riemondy KA, Jansing NL, Jiang P, Redente EF, Gillen AE, Fu R, et al. Single cell RNA sequencing identifies TGFβ as a key regenerative cue following LPS-induced lung injury. *JCI Insight.* 2019;5.
9. Jansing NL, McClendon J, Henson PM, Tuder RM, Hyde DM, and Zemans RL. Unbiased Quantitation of Alveolar Type II to Alveolar Type I Cell Transdifferentiation during Repair after Lung Injury in Mice. *Am J Respir Cell Mol Biol.* 2017;57(5):519-26.
10. Strnad P, Tao GZ, Zhou Q, Harada M, Toivola DM, Brunt EM, et al. Keratin mutation predisposes to mouse liver fibrosis and unmasks differential effects of the carbon tetrachloride and thioacetamide models. *Gastroenterology.* 2008;134(4):1169-79.
11. Ku NO, Liao J, and Omary MB. Phosphorylation of human keratin 18 serine 33 regulates binding to 14-3-3 proteins. *EMBO J.* 1998;17(7):1892-906.

12. Strunz M, Simon LM, Ansari M, Kathiriya JJ, Angelidis I, Mayr CH, et al. Alveolar regeneration through a Krt8+ transitional stem cell state that persists in human lung fibrosis. *Nat Commun.* 2020;11(1):3559.

13. Kobayashi Y, Tata A, Konkimalla A, Katsura H, Lee RF, Ou J, et al. Persistence of a regeneration-associated, transitional alveolar epithelial cell state in pulmonary fibrosis. *Nature cell biology.* 2020;22(8):934-46.

14. Wu H, Yu Y, Huang H, Hu Y, Fu S, Wang Z, et al. Progressive Pulmonary Fibrosis Is Caused by Elevated Mechanical Tension on Alveolar Stem Cells. *Cell.* 2020;180(1):107-21.e17.

15. Wang Y, Tang Z, Huang H, Li J, Wang Z, Yu Y, et al. Pulmonary alveolar type I cell population consists of two distinct subtypes that differ in cell fate. *Proc Natl Acad Sci U S A.* 2018;115(10):2407-12.

16. Adams TS, Schupp JC, Poli S, Ayaub EA, Neumark N, Ahangari F, et al. Single-cell RNA-seq reveals ectopic and aberrant lung-resident cell populations in idiopathic pulmonary fibrosis. *Science Advances.* 2020;6(28):eaba1983.

17. Habermann AC, Gutierrez AJ, Bui LT, Yahn SL, Winters NI, Calvi CL, et al. Single-cell RNA sequencing reveals profibrotic roles of distinct epithelial and mesenchymal lineages in pulmonary fibrosis. *Science Advances.* 2020;6(28):eaba1972.

18. Jiang P, Gil de Rubio R, Hrycaj SM, Gurczynski SJ, Riemondy KA, Moore BB, et al. Ineffectual Type 2-to-Type 1 Alveolar Epithelial Cell Differentiation in Idiopathic Pulmonary Fibrosis: Persistence of the KRT8(hi) Transitional State. *Am J Respir Crit Care Med.* 2020;201(11):1443-7.

19. Yao C, Guan X, Carraro G, Parimon T, Liu X, Huang G, et al. Senescence of Alveolar Type 2 Cells Drives Progressive Pulmonary Fibrosis. *Am J Respir Crit Care Med.* 2021;203(6):707-17.

20. Lee S, Islam MN, Boostanpour K, Aran D, Jin G, Christenson S, et al. Molecular programs of fibrotic change in aging human lung. *Nat Commun.* 2021;12(1):6309.

21. Chen EY, Tan CM, Kou Y, Duan Q, Wang Z, Meirelles GV, et al. Enrichr: interactive and collaborative HTML5 gene list enrichment analysis tool. *BMC Bioinformatics.* 2013;14:128.

22. Kuleshov MV, Jones MR, Rouillard AD, Fernandez NF, Duan Q, Wang Z, et al. Enrichr: a comprehensive gene set enrichment analysis web server 2016 update. *Nucleic acids research.* 2016;44(W1):W90-7.

23. Xie Z, Bailey A, Kuleshov MV, Clarke DJB, Evangelista JE, Jenkins SL, et al. Gene Set Knowledge Discovery with Enrichr. *Current Protocols.* 2021;1(3):e90.

24. Gayoso A, Lopez R, Xing G, Boyeau P, Valiollah Pour Amiri V, Hong J, et al. A Python library for probabilistic analysis of single-cell omics data. *Nat Biotechnol.* 2022;40(2):163-6.

25. McCarthy DJ, Campbell KR, Lun AT, and Wills QF. Scater: pre-processing, quality control, normalization and visualization of single-cell RNA-seq data in R. *Bioinformatics.* 2017;33(8):1179-86.

26. Lun AT, McCarthy DJ, and Marioni JC. A step-by-step workflow for low-level analysis of single-cell RNA-seq data with Bioconductor. *F1000Res.* 2016;5:2122.

27. Satija R, Farrell JA, Gennert D, Schier AF, and Regev A. Spatial reconstruction of single-cell gene expression data. *Nat Biotechnol.* 2015;33(5):495-502.

28. Trapnell C, Cacchiarelli D, Grimsby J, Pokharel P, Li S, Morse M, et al. The dynamics and regulators of cell fate decisions are revealed by pseudotemporal ordering of single cells. *Nat Biotechnol.* 2014;32(4):381-6.

29. Street K, Risso D, Fletcher RB, Das D, Ngai J, Yosef N, et al. Slingshot: cell lineage and pseudotime inference for single-cell transcriptomics. *BMC Genomics.* 2018;19(1):477.

30. Pons P, Latapy M. arXiv [physics.soc-ph]. https://doi.org/10.48550/arXiv.physics/0512106.

31. Travaglini KJ, Nabhan AN, Penland L, Sinha R, Gillich A, Sit RV, et al. A molecular cell atlas of the human lung from single-cell RNA sequencing. *Nature.* 2020;587(7835):619-25.

32. Allen RJ, Stockwell A, Oldham JM, Guillen-Guio B, Schwartz DA, Maher TM, et al. Genome-wide association study across five cohorts identifies five novel loci associated with idiopathic pulmonary fibrosis. *Thorax.* 2022.

33. Boughton AP, Welch RP, Flickinger M, VandeHaar P, Taliun D, Abecasis GR, et al. LocusZoom.js: Interactive and embeddable visualization of genetic association study results. *Bioinformatics.* 2021;37(18):3017-8.

34. Rentzsch P, Schubach M, Shendure J, and Kircher M. CADD-Splice—improving genome-wide variant effect prediction using deep learning-derived splice scores. *Genome Medicine.* 2021;13(1):31.

35. Smedley D, Schubach M, Jacobsen JOB, Köhler S, Zemojtel T, Spielmann M, et al. A Whole-Genome Analysis Framework for Effective Identification of Pathogenic Regulatory Variants in Mendelian Disease. *Am J Hum Genet.* 2016;99(3):595-606.

36. Jansing NL, McClendon J, Kage H, Sunohara M, Alvarez JR, Borok Z, et al. Isolation of Rat and Mouse Alveolar Type II Epithelial Cells. *Methods Mol Biol.* 2018;1809:69-82.

37. Morales Johansson H, Newman DR, and Sannes PL. Whole-genome analysis of temporal gene expression during early transdifferentiation of human lung alveolar epithelial type 2 cells in vitro. *PLoS One.* 2014;9(4):e93413.

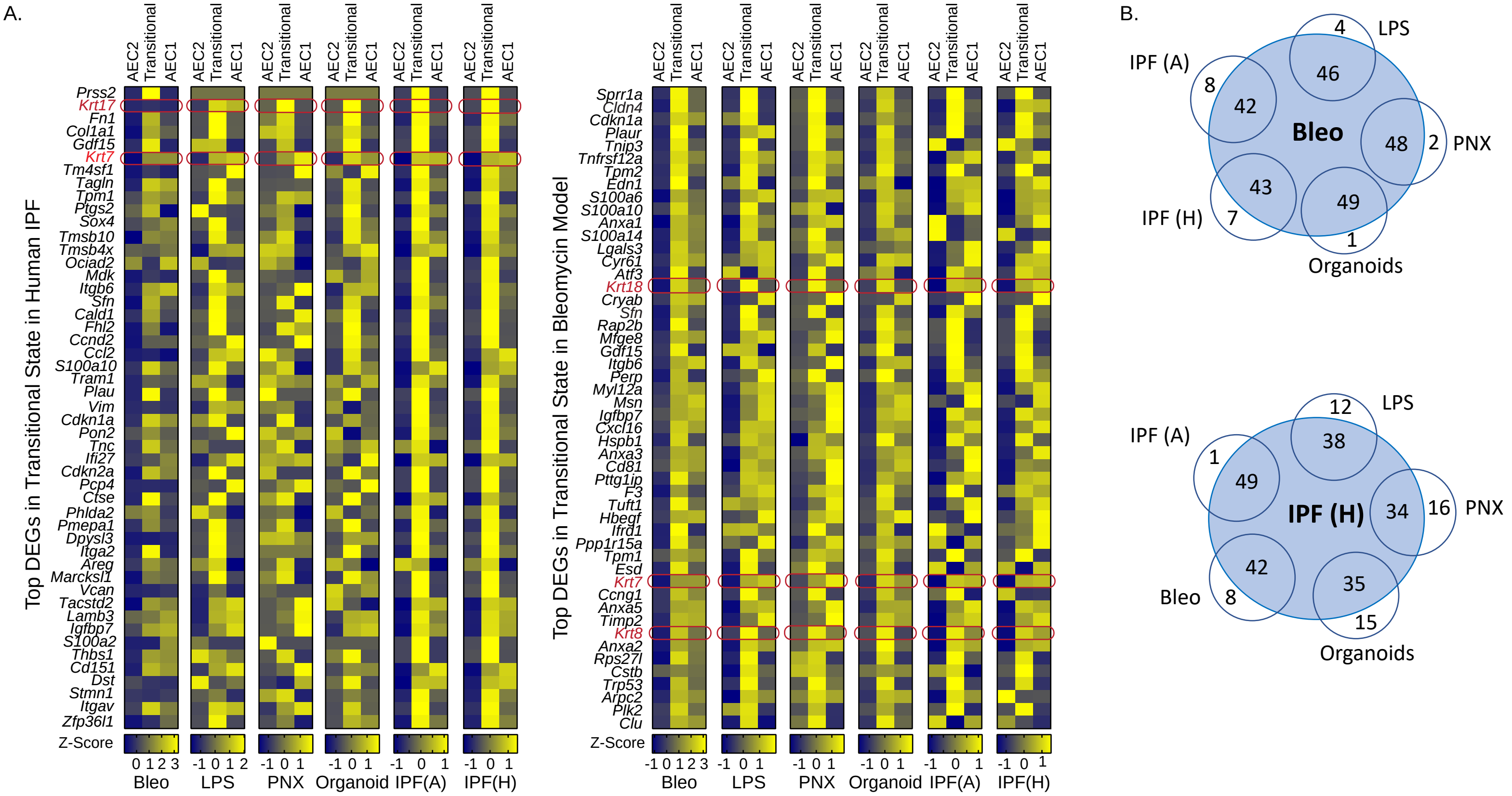

Supplemental Figure 1. Keratin^{hi} Transitional State is Conserved Across Multiple Mouse Models of Lung Regeneration and Human IPF. A,B,F,G) scRNAseq datasets from the bleomycin (54), LPS (50), pneumonectomy (56), and organoid (53) mouse models and human IPF (57, 48) were analyzed. **A)** Expression levels of the top differentially expressed genes (DEGs) in the transitional (*KRT5-*/*KRT17+*) state in human IPF (58) and the top DEGs in the transitional (*Krt8+* ADI) state in the bleomycin mouse model (57) are shown. **B)** Venn diagrams of expression of the top 50 DEGs in the transitional state in the bleomycin model (left) or IPF (right). Overlap indicates the number of genes that are also upregulated in the transitional state in the other datasets. The transcriptome of the transitional state is conserved across multiple mouse models and human IPF. **C-E,H)** Mice were treated with bleomycin or LPS or subjected to pneumonectomy and stained with indicated antibodies **(C,E)**, FISH probe **(H)**, or trichrome or picrosirius red **(D)**. **C)** Arrowheads indicate transitional cells. Image from Figure 1B shown here as separate channels. Bottom panel reveals K8 expression in naïve AEC2s with higher exposure time and gain. Transitional cells were quantitated. The PNX and LPS models have **D)** minimal fibrosis and **E)** rare transitional cells in peripheral lung (insets 1 and 2) with largely normal appearing lung (insets 3 and 4 and the majority of the tissue), whereas the bleomycin model is characterized by extensive fibrosis and abundant transitional cells (inset 5 and the majority of the tissue). **E)** Scale bar: 1 mm. **F)** The top 400 DEGs expressed by transitional cells compared to other epithelial cells in the bleomycin (54) and LPS (50) mouse models were subjected to KEGG pathway analysis. Adjusted p<0.05 for all pathways shown except mitophagy in the LPS model and chemokine signaling in the LPS and bleomycin models. Multiple pathways by which epithelial cells are known to promote fibrosis, including cell death (apoptosis), proteostasis (protein processing in endoplasmic reticulum, autophagy), senescence (cellular senescence, p53 signaling), and chemokine signaling, are highly differentially activated in the transitional state as compared to other epithelial cell states. **G)** Transitional cells in mouse models and human IPF upregulate SASP genes. **H)** Quantitation demonstrates that most transitional (K8^{hi}) cells express *Itgb6,* consistent with TGFβ activation, though this may be an underestimate if *Itgb6* RNA molecules are present in a part of highly spread cells that is not in the plane of the tissue section. Scale bars: 100 µm unless otherwise indicated. For staining, n=3 mice/group.

C.

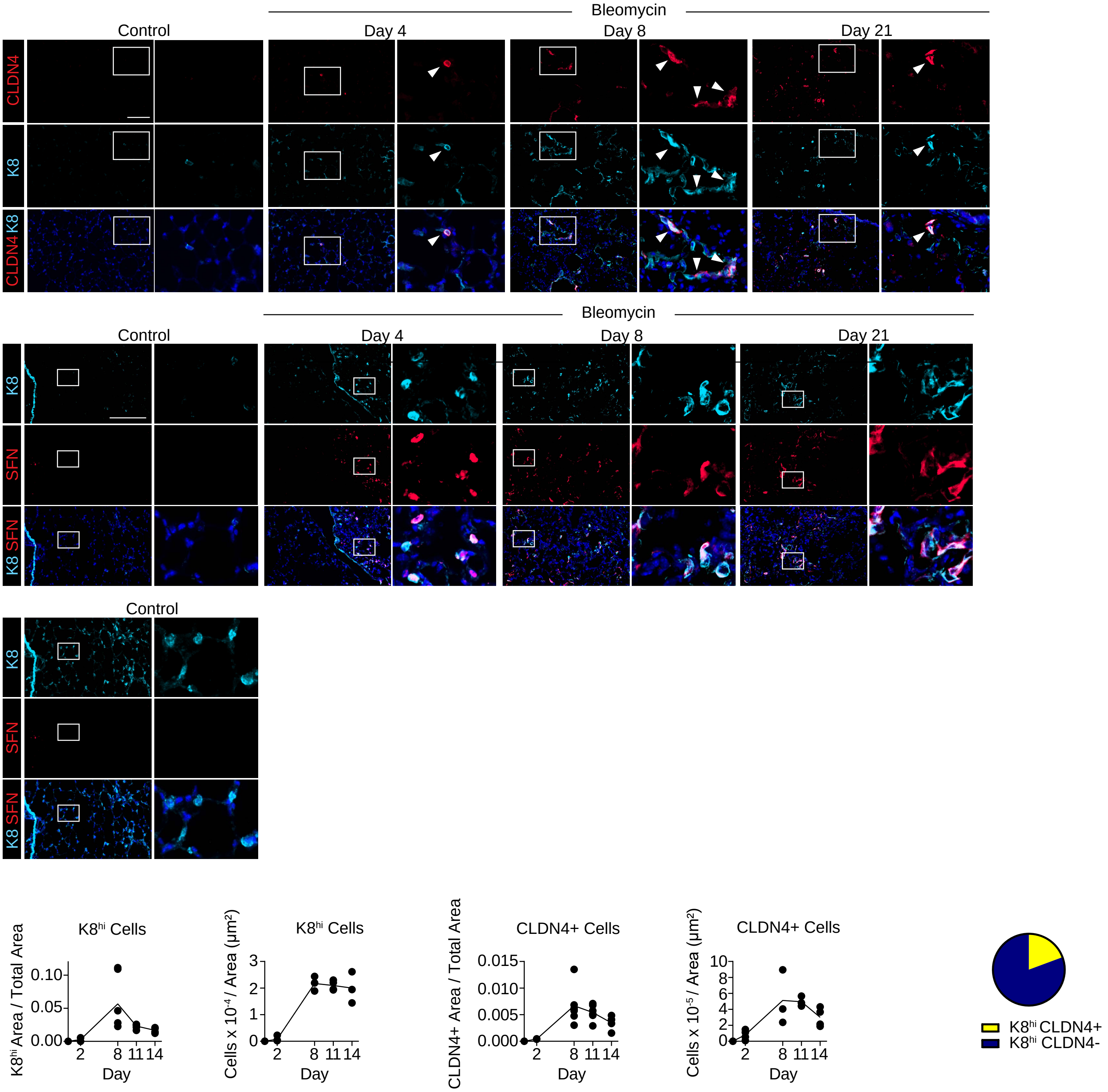
Bleomycin
Control
Day 4
Day 8
Day 21
CLDN4
K8
CLDN4 K8
Bleomycin
Control
Day 4
Day 8
Day 21
K8
SFN
K8 SFN
Control
K8
SFN
K8 SFN
K8hi Cells
K8hi Area / Total Area
0.10
0.05
0.00
2
8
11
14
Day
K8hi Cells
Cells x 10-4 / Area (µm2)
3
2
1
0
2
8
11
14
Day
CLDN4+ Cells
CLDN4+ Area / Total Area
0.015
0.010
0.005
0.000
2
8
11
14
Day
CLDN4+ Cells
Cells x 10-5 / Area (µm2)
10
8
6
4
2
0
2
8
11
14
Day
K8hi CLDN4+
K8hi CLDN4-

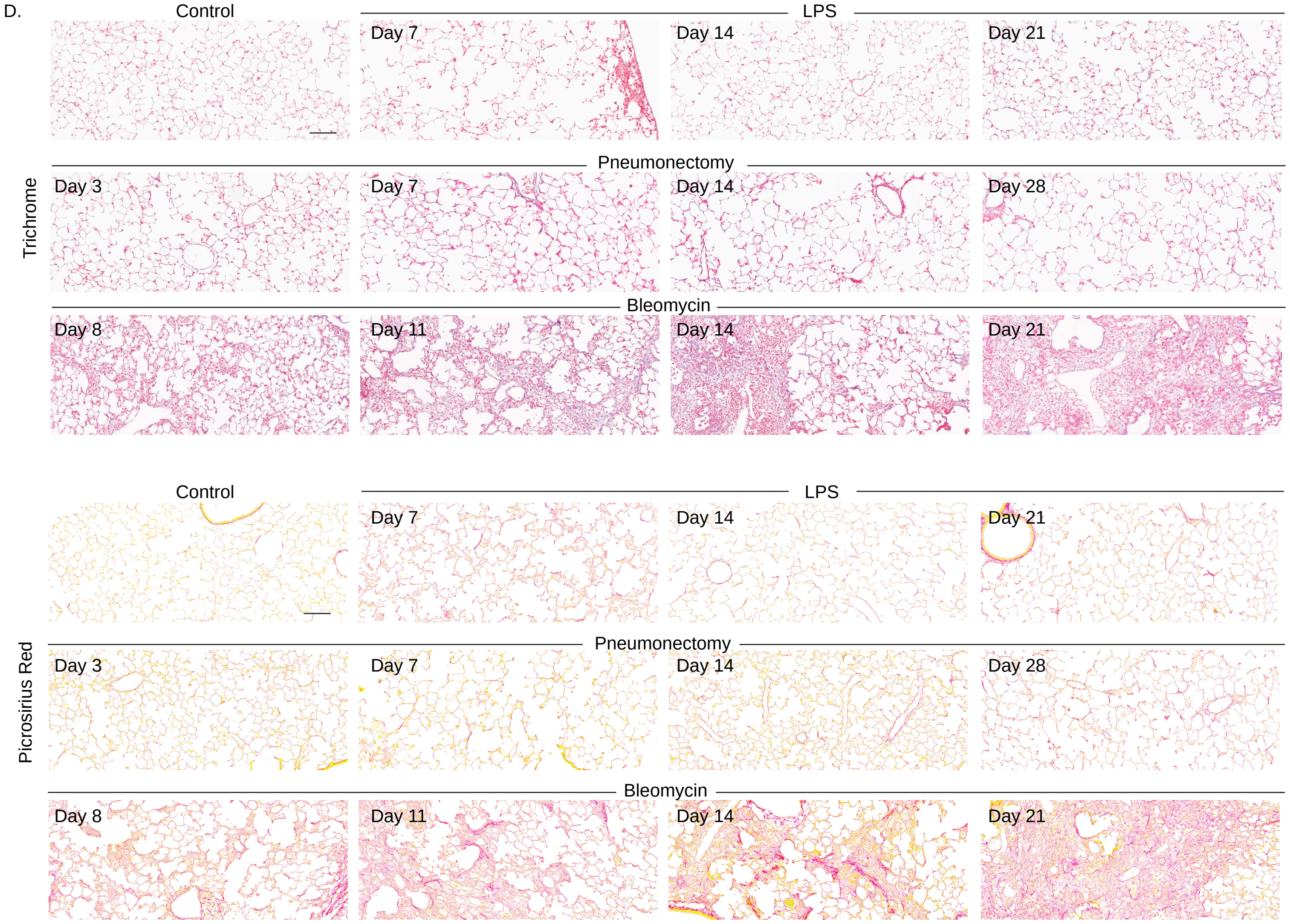
D.
Control
LPS
Day 7
Day 14
Day 21
Trichrome
Pneumonectomy
Day 3
Day 7
Day 14
Day 28
Bleomycin
Day 8
Day 11
Day 14
Day 21
Control
LPS
Day 7
Day 14
Day 21
Picrosirius Red
Pneumonectomy
Day 3
Day 7
Day 14
Day 28
Bleomycin
Day 8
Day 11
Day 14
Day 21

E.

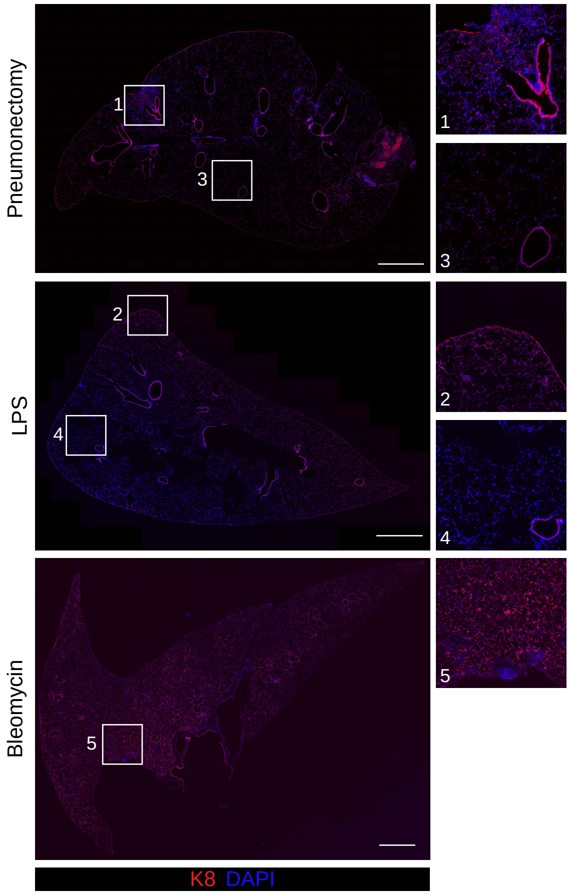

Pneumonectomy
LPS
Bleomycin
1
2
3
4
5
K8 DAPI

F.

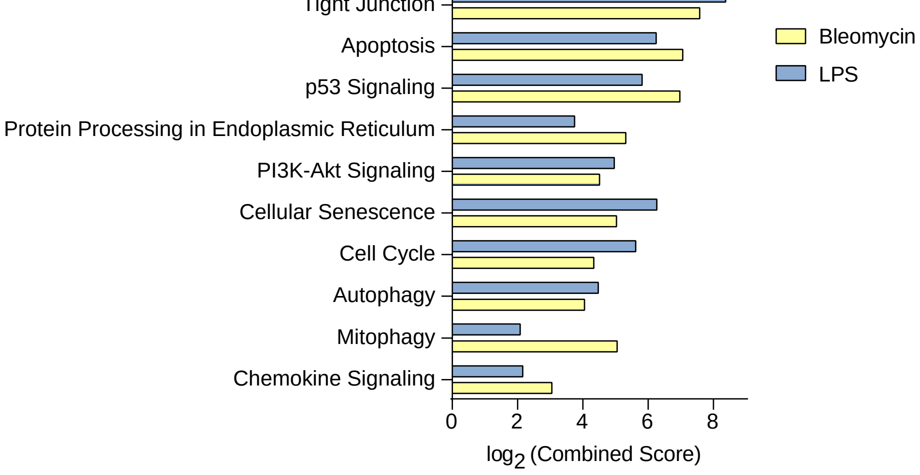

Tight Junction
Apoptosis
p53 Signaling
Protein Processing in Endoplasmic Reticulum
PI3K-Akt Signaling
Cellular Senescence
Cell Cycle
Autophagy
Mitophagy
Chemokine Signaling
Bleomycin
LPS
0
2
4
6
8
log2 (Combined Score)

G.

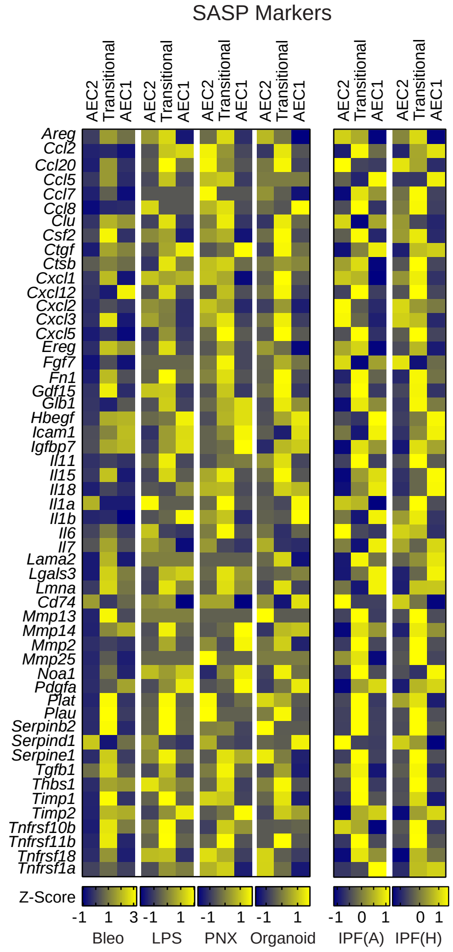

SASP Markers
AEC2
Transitional
AEC1
Areg
Ccl2
Ccl20
Ccl5
Ccl7
Ccl8
Clu
Csf2
Ctgf
Ctsb
Cxcl1
Cxcl12
Cxcl2
Cxcl3
Cxcl5
Ereg
Fgf7
Fn1
Gdf15
Glb1
Hbegf
Icam1
Igfbp7
Il11
Il15
Il18
Il1a
Il1b
Il6
Il7
Lama2
Lgals3
Lmna
Cd74
Mmp13
Mmp14
Mmp2
Mmp25
Noa1
Pdgfa
Plat
Plau
Serpinb2
Serpind1
Serpine1
Tgfb1
Thbs1
Timp1
Timp2
Tnfrsf10b
Tnfrsf11b
Tnfrsf18
Tnfrsf1a
Z-Score
-1 1 3
-1 1
-1 1
-1 1
-1 0 1
0 1
Bleo
LPS
PNX
Organoid
IPF(A)
IPF(H)

H.

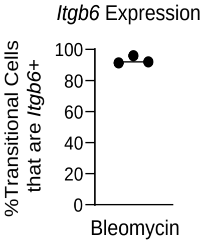

Itgb6 Expression
%Transitional Cells that are Itgb6+
100
80
60
40
20
0
Bleomycin

A.

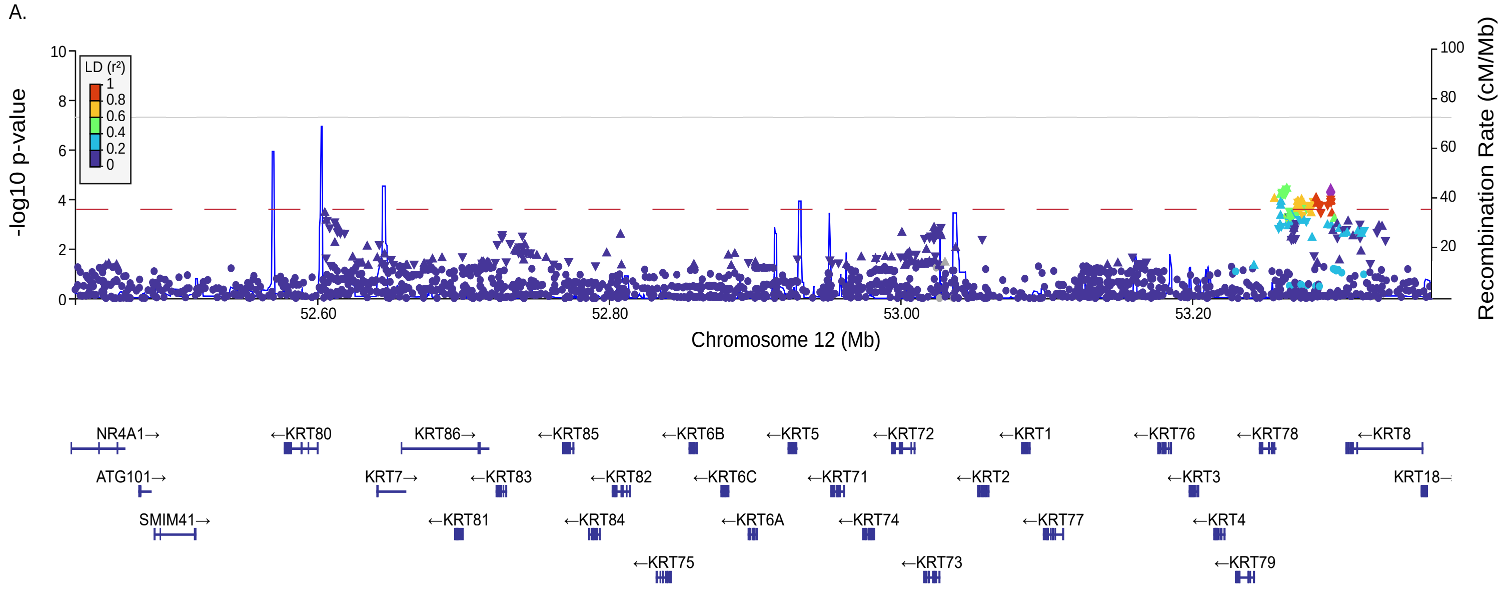

B.

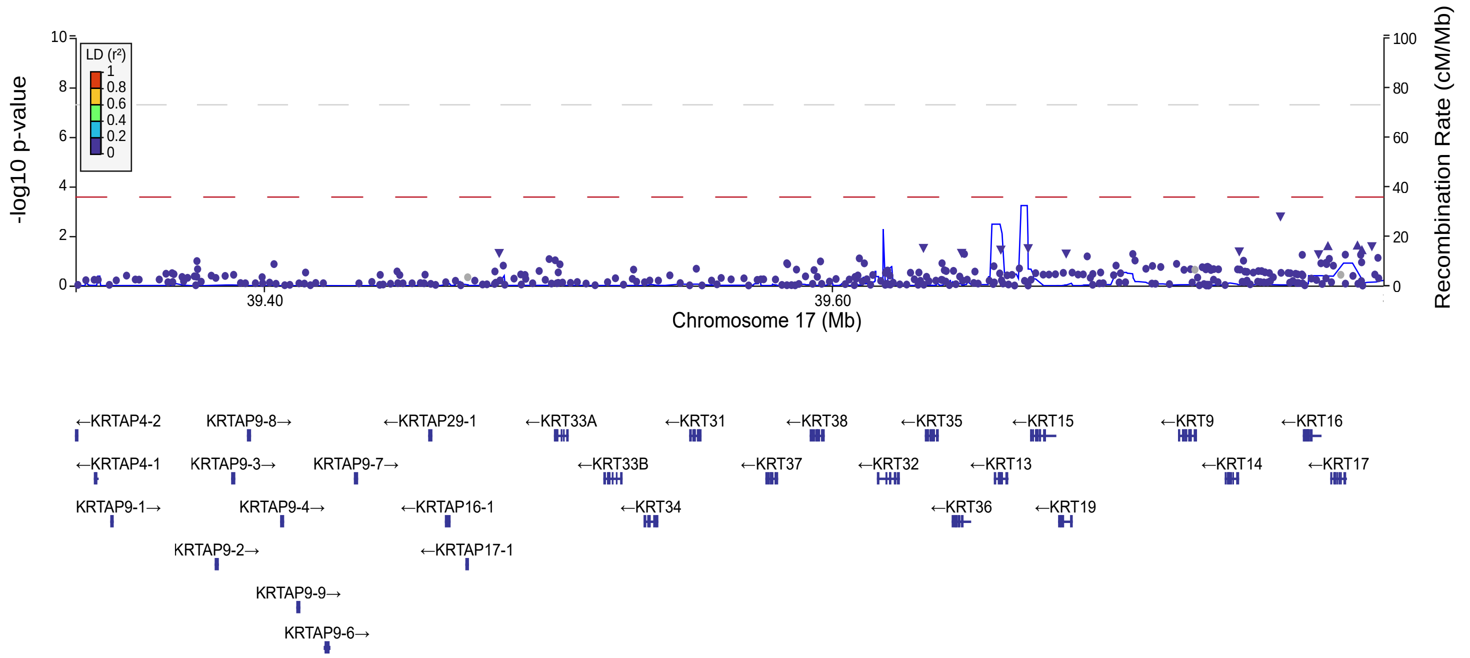

Supplemental Figure 2. Human *KRT8* Genetic Variants are Associated with IPF. Using a genome-wide association meta-analysis of IPF (74), a nested candidate gene study for the keratins expressed in the transitional state was performed. A regional association plot showing all SNPs that overlap with keratin genes is shown. Grey dotted line indicates genome-wide significance. Red dotted line indicates statistical significance of the nested candidate gene study for the keratin genes. Blue curve indicates the estimated recombination rate. Seven *KRT8* SNPs were associated with IPF ($p < 1.4 \times 10^{-4}$). No SNPs in other keratin genes were associated with IPF.

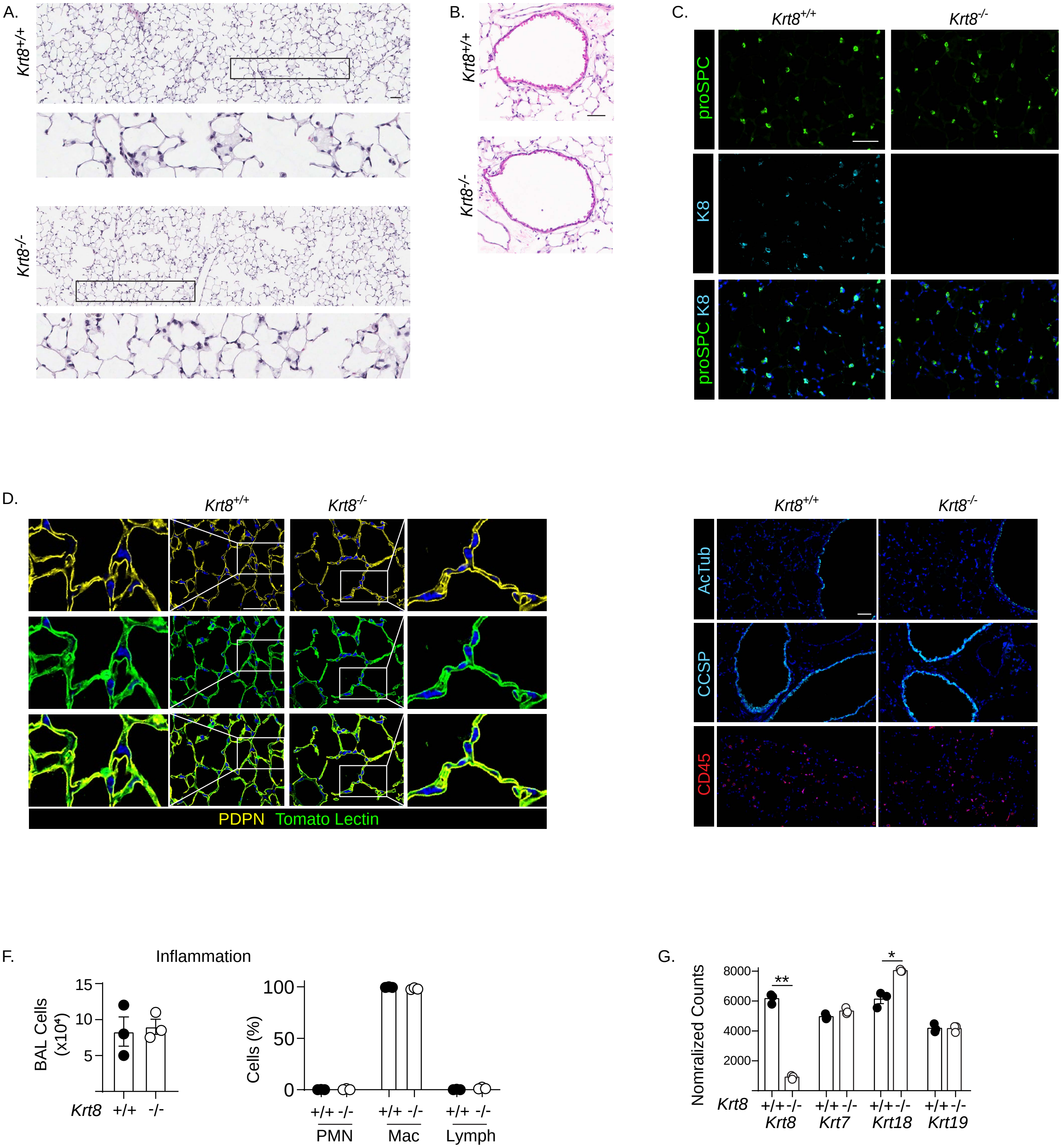

Supplemental Figure 3. *Krt8*$^{-/-}$ Mice Have Grossly Normal Lungs and are Protected from Fibrosis. Naïve **(A-I)** or bleomycin-treated **(J)** adult *Krt8*$^{+/+}$ and *Krt8*$^{-/-}$ mice were euthanized. Lungs were fixed and stained for H&E **(A,B,J)** or with indicated antibodies **(C-E,H)**. **F)** BAL cell counts and differentials were determined. **G)** AEC2s were isolated, and RNA was purified and subjected to bulk RNAseq. No abnormalities were detected in lung structure **(A,B)**, epithelial cell makeup **(C-E)**, BAL cellularity **(F)**, or expression of other simple keratins in *Krt8*$^{-/-}$ mice **(G,H)**. **I)** Adult male *Krt8*$^{-/-}$ mice weighed less than *Krt8*$^{+/+}$ mice. Data represented as mean ± SEM.*$p<0.05$ by t test **(G)** or multiple t test discovery determined using the two-stage linear step-up procedure of Benjamini, Krieger, and Yekutieli with Q=1% **(I)**. **J)** Focal airspace dilation adjacent to areas of fibrosis noted in bleomycin-treated *Krt8*$^{+/+}$ and *Krt8*$^{-/-}$ lungs. Scale bars: 50 µm **(A-E,H),** 2 mm **(J)**. For immunostaining, n=3 mice/group.

H.

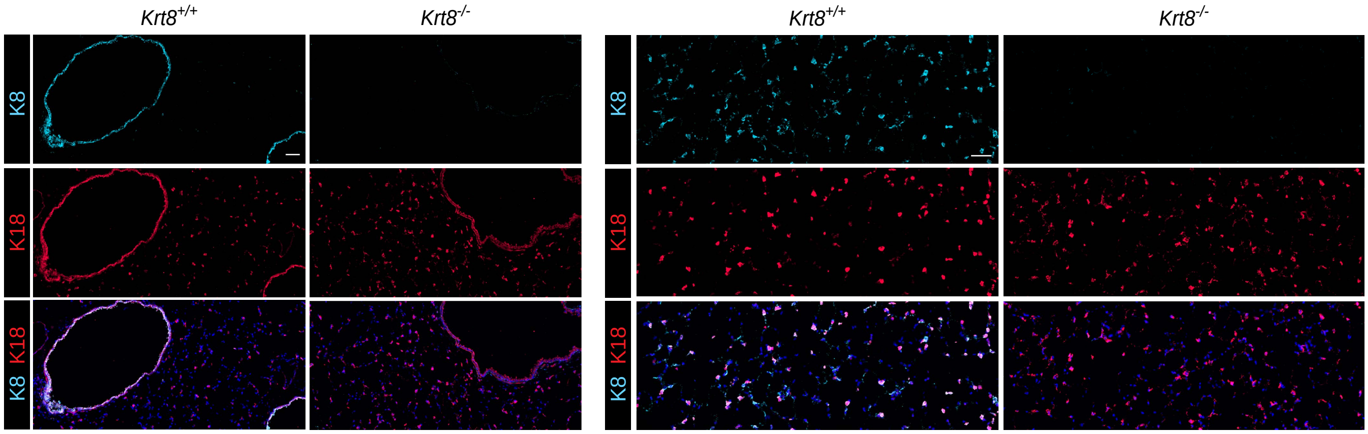

Krt8+/+
Krt8-/-
Krt8+/+
Krt8-/-
K8
K18
K8 K18
K8
K18
K8 K18

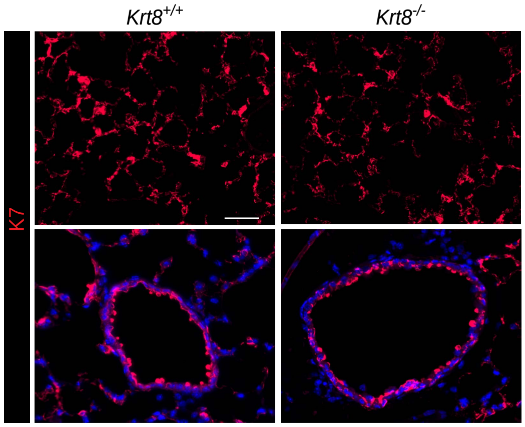

Krt8+/+
Krt8-/-
K7

I.

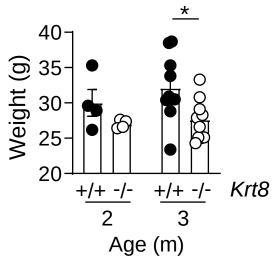

*
40
35
30
25
20
Weight (g)
+/+ -/-
+/+ -/-
Krt8
2
3
Age (m)

J.

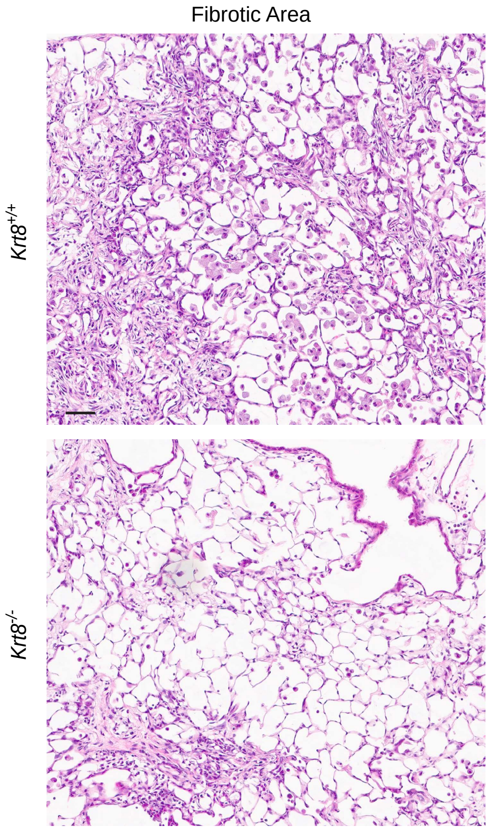

Fibrotic Area
Krt8+/+
Krt8-/-

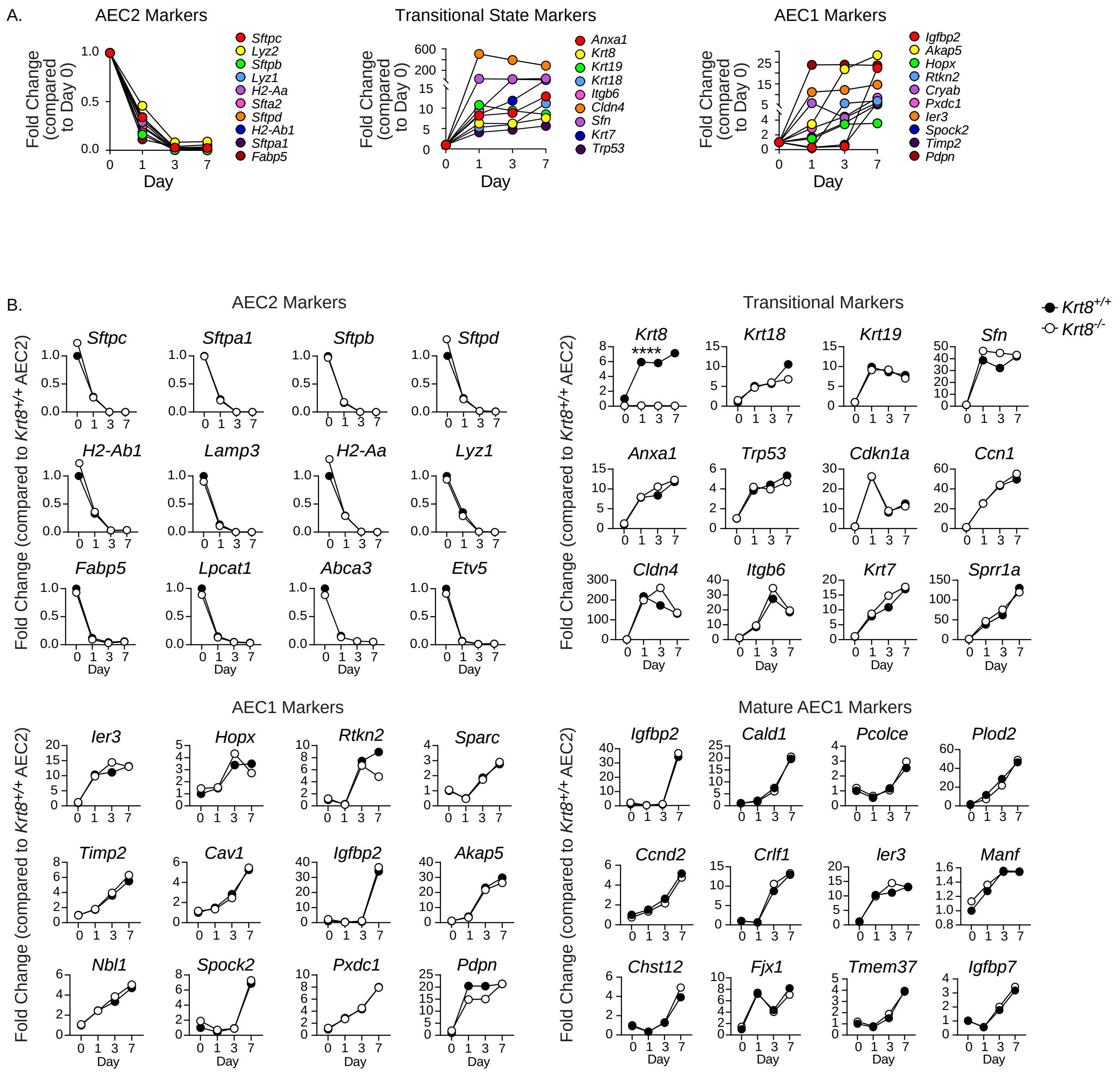

Supplemental Figure 4. Keratin 8 Promotes Expression of Chemokines but not Accumulation of Transitional Cells at the Expense of AEC1 Differentiation. AEC2s were isolated from *Krt8*$^{+/+}$ **(A-D,F)** and *Krt8*$^{-/-}$ **(B-D,F)** mice, cultured in 2D, and harvested at the indicated time points. RNA sequencing **(A,B,D,F)** or fixation and immunostaining **(C)** was performed. **A)** AEC2 markers are downregulated, whereas transitional state and AEC1 markers are upregulated in culture. q-value<0.05 for all markers at Days 1, 3, and 7 compared to Day 0 except *Cav1*, *Hopx*, *Sftpc*, and *Lyz2* for which q-value<0.05 for Days 3, 7 compared to Day 0. **B)** *Krt8* deficiency did not alter expression of AEC2, transitional, or AEC1 markers as AEC2s differentiate in culture. ****p<0.0001 by unpaired t test on area under the curve (AUC) from Day 1-7 for *Krt8*$^{+/+}$ vs. *Krt8*$^{-/-}$ cells. n=3. **C)** There was no gross difference in cell spreading as AECs differentiate in culture. Scale bar: 50 µm. **D)** PCA plot using the top 500 variable genes revealed that transcriptomes varied by time point but not by genotype. n=3, with circles, triangles, and squares each representing an individual experiment. **E)** MLE-12 cells were transduced with lentivirus containing one of two distinct *Krt8* shRNA constructs or a scrambled (SCR) shRNA and stimulated with tunicamycin. K8 was not necessary for ER stress. n=2. **F)** K8 promotes expression of multiple chemokines. *p<0.05 by t test of AUC. n=3. **G)** Mice were treated with bleomycin and euthanized at the indicated time points. CCL2 ELISA was performed on BAL fluid. **A,B,E,F,G)** Line at mean.

C.

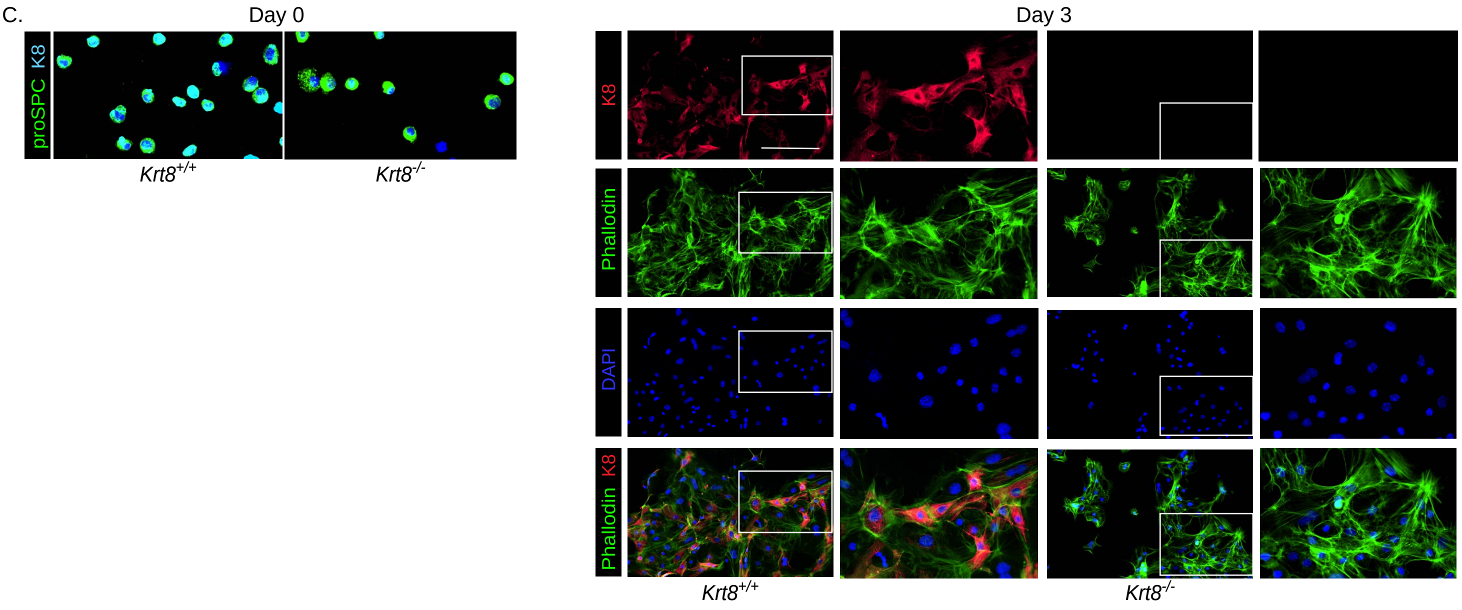

Day 0
Day 3
proSPC K8
Krt8+/+
Krt8-/-
K8
Phallodin
DAPI
Phallodin K8
Krt8+/+
Krt8-/-

D.

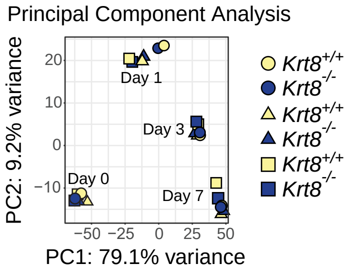

Principal Component Analysis
PC2: 9.2% variance
PC1: 79.1% variance
Day 0
Day 1
Day 3
Day 7
Krt8+/+
Krt8-/-
Krt8+/+
Krt8-/-
Krt8+/+
Krt8-/-

E.

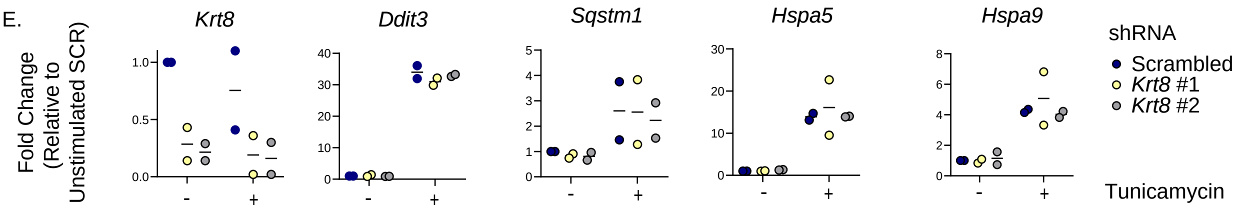

Fold Change (Relative to Unstimulated SCR)
Krt8
Ddit3
Sqstm1
Hspa5
Hspa9
shRNA
Scrambled
Krt8 #1
Krt8 #2
Tunicamycin

F.

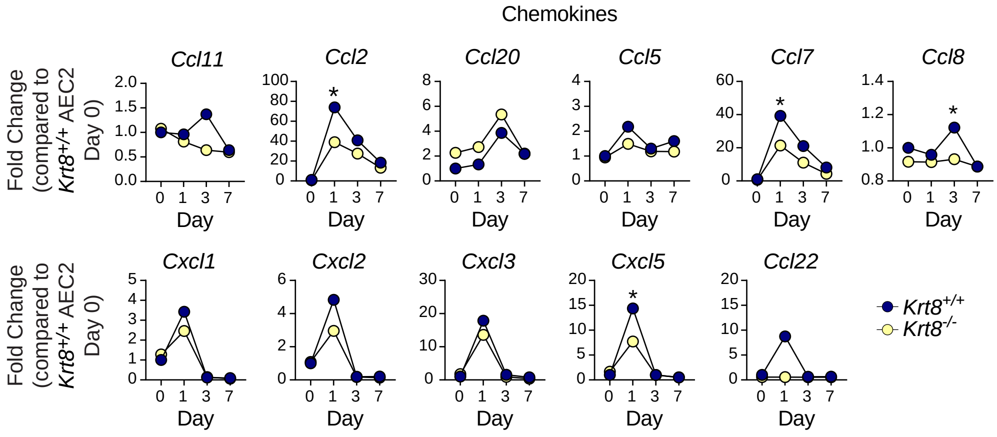

Chemokines
Fold Change (compared to Krt8+/+ AEC2 Day 0)
Ccl11
Ccl2
Ccl20
Ccl5
Ccl7
Ccl8
Cxcl1
Cxcl2
Cxcl3
Cxcl5
Ccl22
Day
Krt8+/+
Krt8-/-

G.

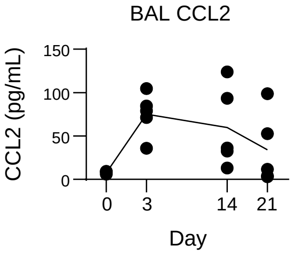

BAL CCL2
CCL2 (pg/mL)
Day

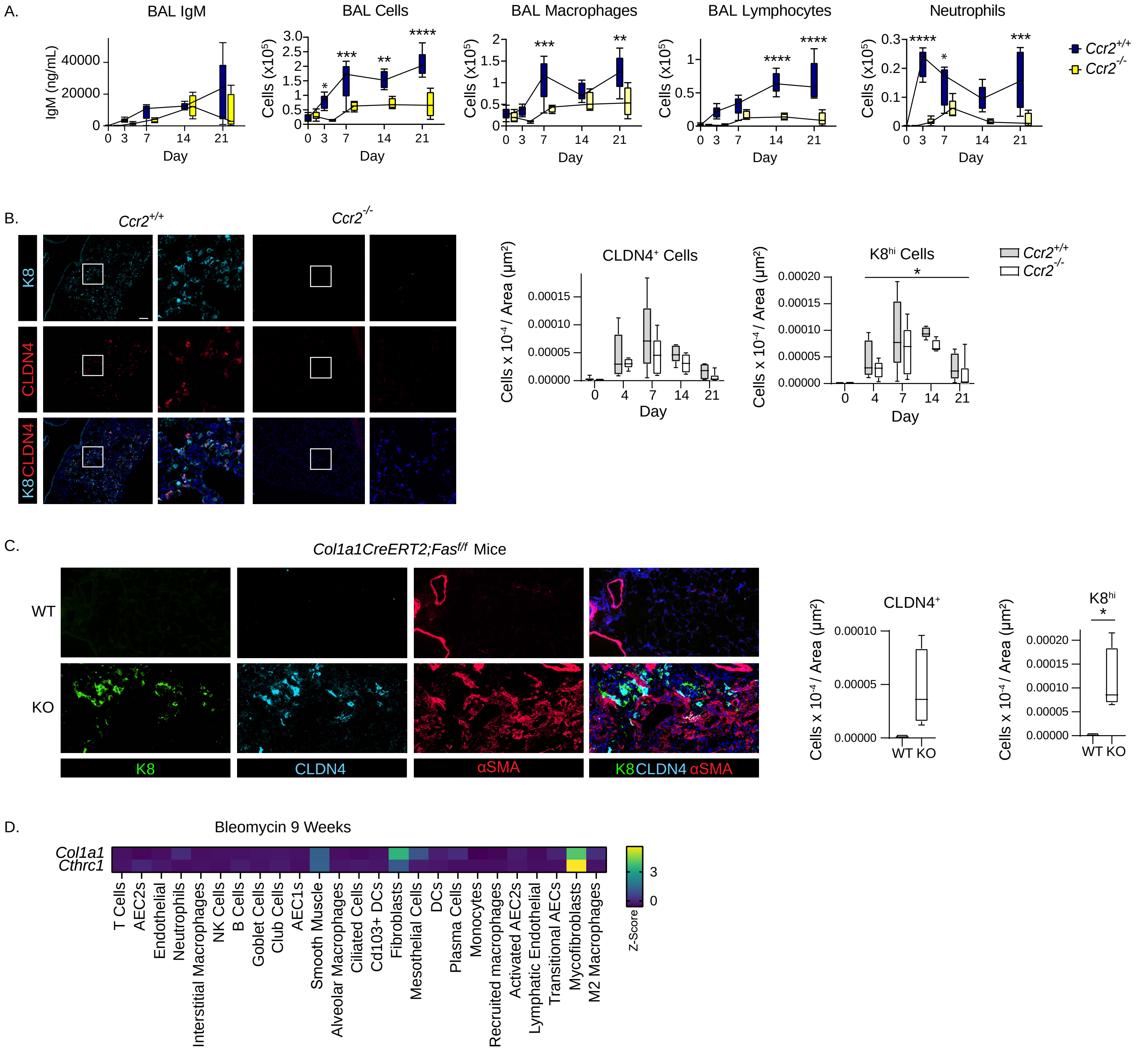

Supplemental Figure 5. Macrophages and Myofibroblasts Promote Accumulation of Transitional AECs. A,B) *Ccr2*$^{+/+}$ or *Ccr2*$^{-/-}$ mice were treated with bleomycin and harvested at indicated time points. **A)** IgM ELISA and cell counts were performed on BAL. *p<0.05, **p<0.01, ***p<0.001, ****p<0.0001 for *Ccr2*$^{+/+}$ or *Ccr2*$^{-/-}$ by two-way ANOVA with Sidak's posthoc multiple comparisons test. **B)** Lung sections were immunostained, and transitional cells were quantified. Scale bar: 100 µm. Images are the same as those shown in Figure 5B, with channels separated here. Macrophage recruitment promoted transitional state accumulation. **C)** *Col1a1CreERT2;Fas*$^{f/f}$ mice were treated with bleomycin, administered tamoxifen (KO) or corn oil (WT), and euthanized at 9 wk. Scale bar: 100 µm. Fibroblast persistence was sufficient for transitional AEC persistence. **D)** Heatmap showing *Col1a1* and *Cthrc1* expression in various cell types in the bleomycin mouse model, as determined by scRNAseq (54). **E)** *Col1a1CreERT2;tdTomato* mice were treated with bleomycin, administered tamoxifen, and euthanized at Day 21. Lung sections were immunostained. Scale bars: 50 µm. Cre-mediated recombination resulting in tdTomato expression was not induced in epithelial, endothelial, or immune cells. The PDGFRA staining had high background but images suggest PDGFRA+ tdTomato+ cells were detected. **F)** AEC2s were isolated from wildtype mice, cultured for 7 d, fixed, and immunostained. Scale bars: 50 µm. **G)** Mice were subjected to PNX or treated with LPS or bleomycin. Immunostaining was performed. Image from Figure 5G is shown here as separate channels. Transitional cells were found in small foci of fibrosis in peripheral lung of in the PNX and LPS mouse models (*), whereas most of the lung was devoid of fibrosis and transitional cells (**). In the bleomycin model, large regions of lung were characterized by fibrosis accompanied by transitional cells (*), whereas some regions were devoid of fibrosis and transitional cells (**). Scale bar: 100 µm. **H)** *SftpcCreERT2;mTmG* mice were subjected to pneumonectomy and euthanized at Day 7. AEC2 proliferation was prevalent. Scale bar: 100 µm. **I)** Pseudotime analysis of the LPS scRNAseq dataset performed by PAGA, Slingshot, and RNA velocity suggest a lineage trajectory through which AEC2s bypass the transitional state and differentiate into AEC1s. PAGA and velocity images are reproduced from (50). For immunostaining, n=3 mice/group.

E. *Col1a1CreERT2;tdTomato* Mice

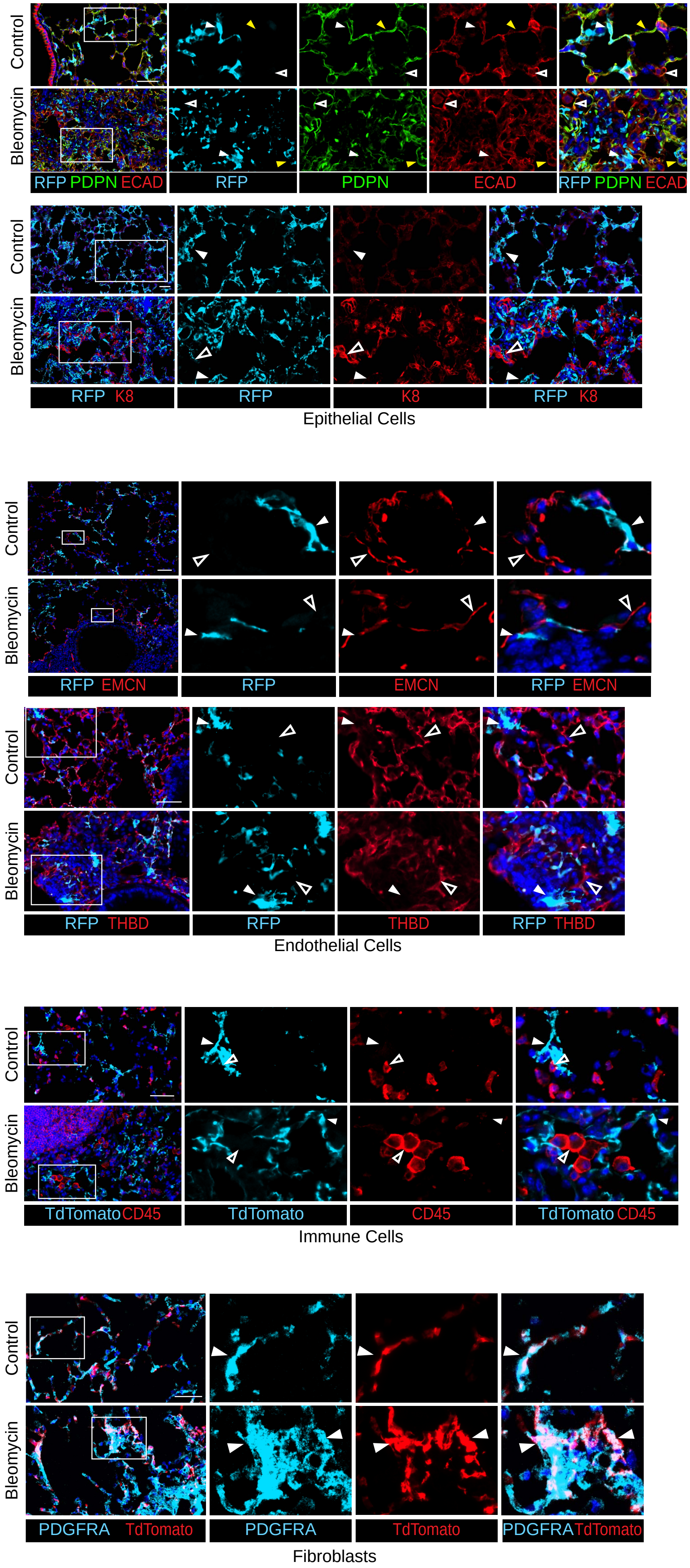

F.

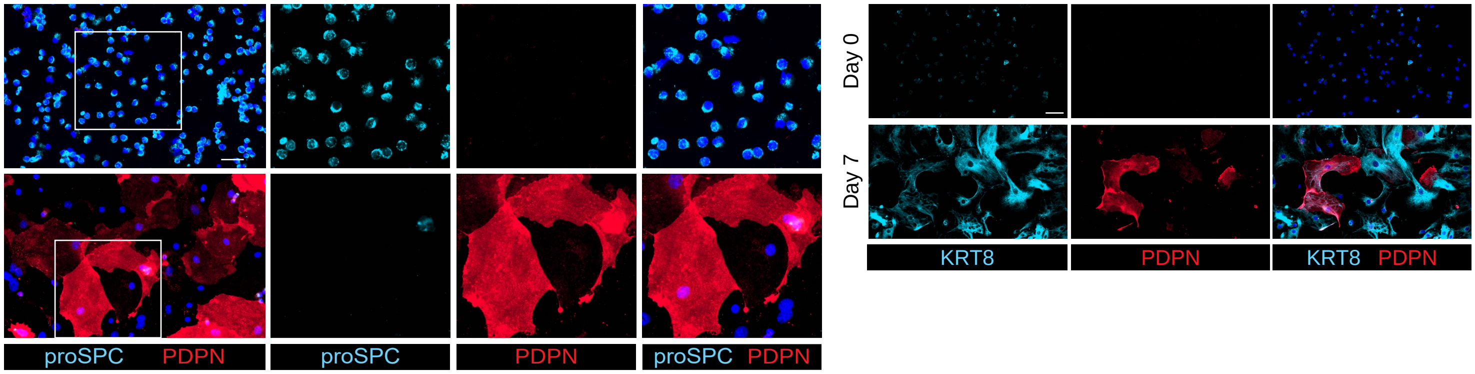
Day 0
Day 7
proSPC PDPN
proSPC
PDPN
proSPC PDPN
Day 0
Day 7
KRT8
PDPN
KRT8 PDPN

G.

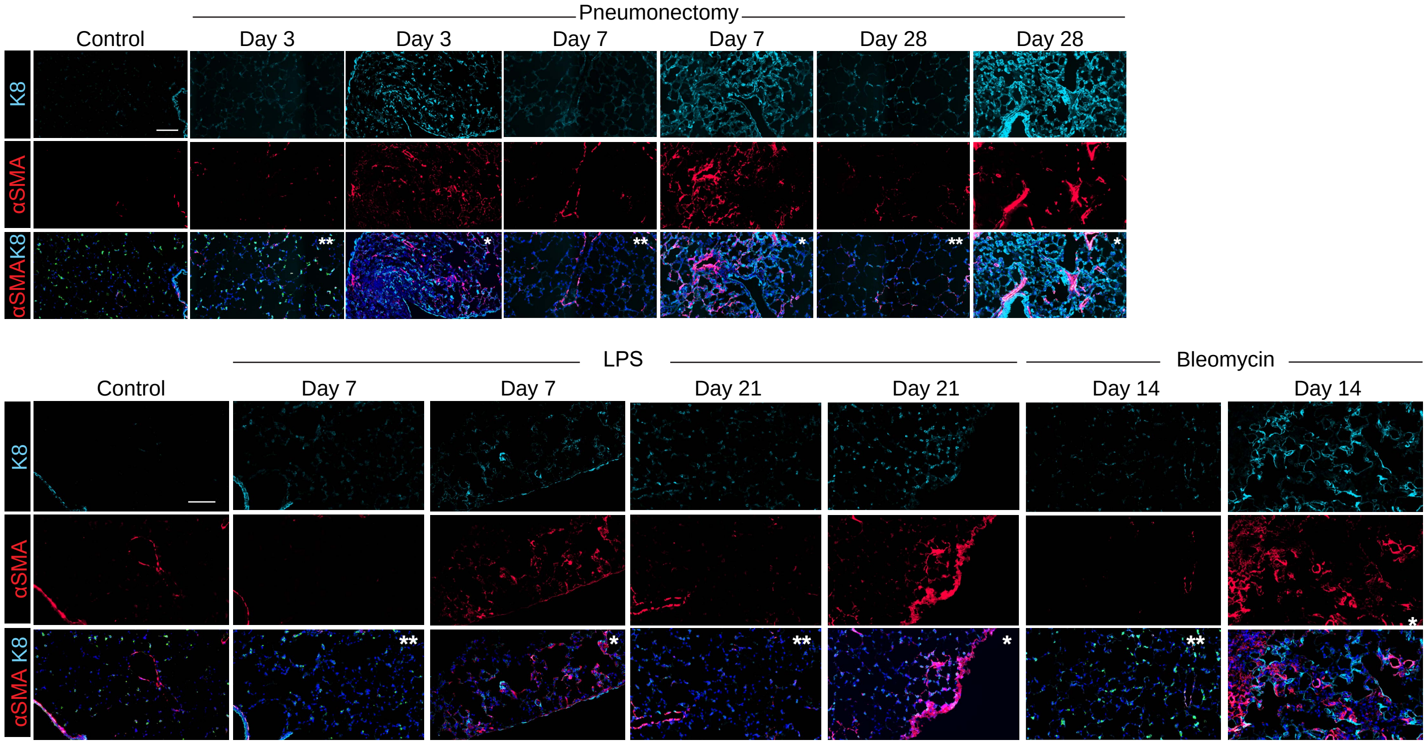
Pneumonectomy
Control
Day 3
Day 3
Day 7
Day 7
Day 28
Day 28
K8
αSMA
αSMA K8
**
*
**
*
**
*
LPS
Bleomycin
Control
Day 7
Day 7
Day 21
Day 21
Day 14
Day 14
K8
αSMA
αSMA K8
**
*
**
*
**
*

H.

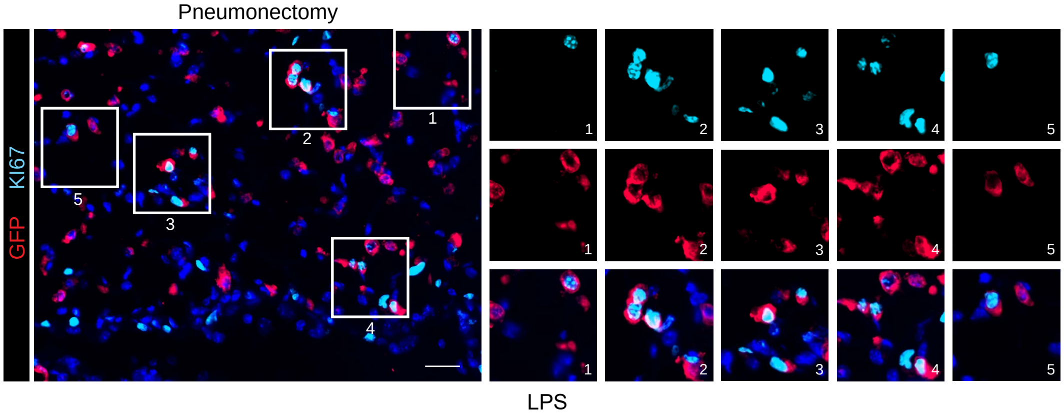
Pneumonectomy
GFP KI67
1
2
3
4
5
LPS

I.

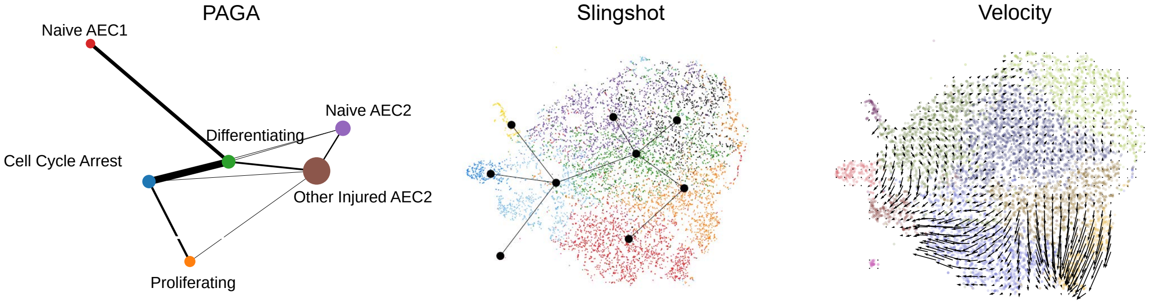
PAGA
Naive AEC1
Naive AEC2
Differentiating
Cell Cycle Arrest
Other Injured AEC2
Proliferating
Slingshot
Velocity

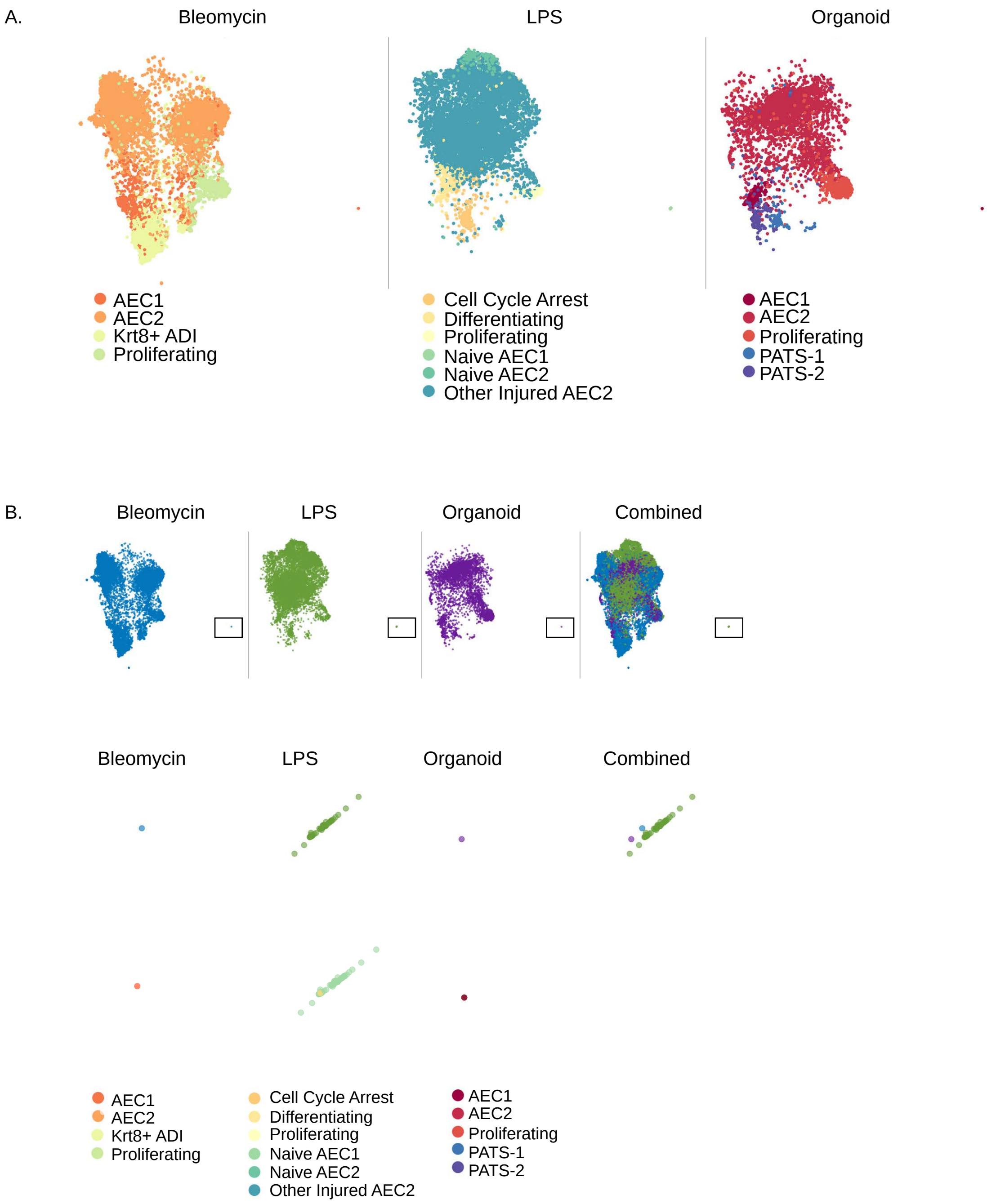

Supplemental Figure 6. Meta-analysis of scRNAseq Datasets from the Bleomycin, LPS, and Organoid Models of Alveolar Regeneration. scRNAseq datasets from the bleomycin (54), LPS (50), and organoid (53) models were integrated. **A)** Cell types and states identified in the original scRNAseq studies. **B)** A discrete cluster of mature AEC1s, contained mainly cells isolated from naïve mice using an enzymatic digestion and centrifugation protocol designed to preserve fragile AEC1s (83) and an antibody cocktail specific for flow assisted cell sorting (FACS) of AEC1s. Most cells with high expression of AEC1 markers appear to be progenitors in the process of AEC1 differentiation, with mature AEC1s lost in the tissue digest.

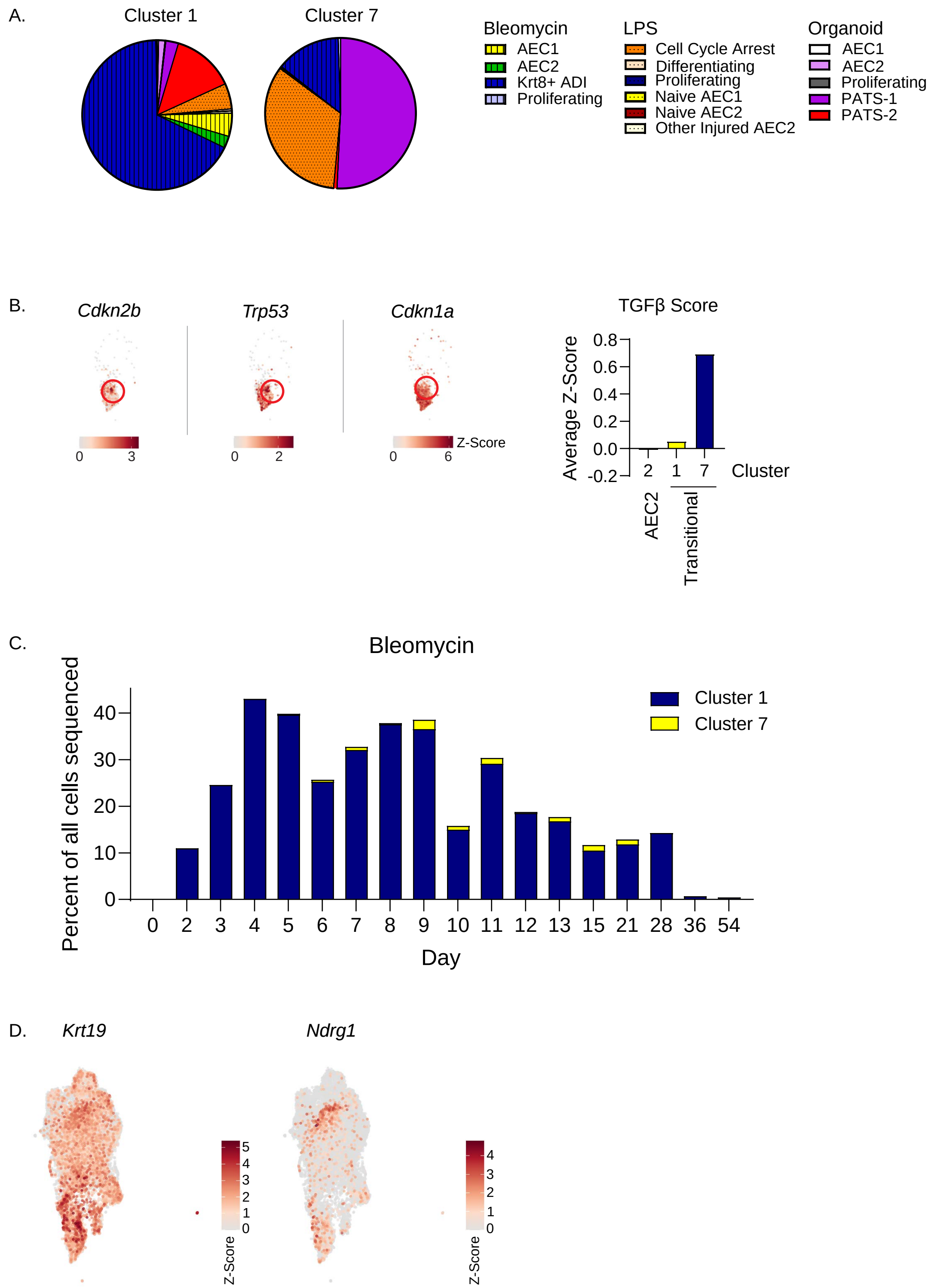

Supplemental Figure 7. Murine Transitional Cell Subset Recapitulates the Human IPF Senescent, Aberrant Basaloid State and Lack of AEC1 Fate. A-F,H,J) Integration of bleomycin, LPS, and organoid scRNAseq datasets. **A)** Both transitional state clusters (Clusters 1 and 7) exist in all 3 models. **B)** Compared to cells in Cluster 1, cells in Cluster 7 are characterized by high levels of senescence and TGFβ activation. **C)** Time course of emergence of cells in Clusters 1 and 7 in the bleomycin model. **D)** *Krt19* and *Ndrg1*, previously used as *Cre* drivers for lineage tracing of the transitional state, are highly expressed in the transitional state but are not specific to Cluster 7. **E)** Pseudotime analysis using Monocle. **F)** Top DEGs in Cluster 7 of the transitional state shown in all clusters and models. **G)** Expression of murine Cluster 7 markers in human IPF. **H)** Expression of the basal cell markers found in the top 100 DEGs of the human IPF transitional state, shown in each mouse model. Cells in Cluster 7 upregulated basal cell genes. **I)** Expression of basaloid genes in cultured murine AECs. q-value<0.05 for all genes except *Sox4*, *S100a2*, *Phlda2, Lamb3,* and *Zfp36l1* at Day 1, *Sox4*, *S100a2*, and *Zfp36l1* at Day 3, and *S100a2* and *Atf3* at Day 7. **J)** Rare K17+ transitional cells arise in the single bleomycin model. Scale bars: 200 μm. **K)** Many transitional (K8^{hi}) cells in human IPF but not in human ARDS are in the K17+ aberrant basaloid state. Merged inset for IPF is the same image shown in Figure 7N, shown here in separate channels. Scale bars: 500 μm. **J,K)** n=3 mice or patients/group.

E.

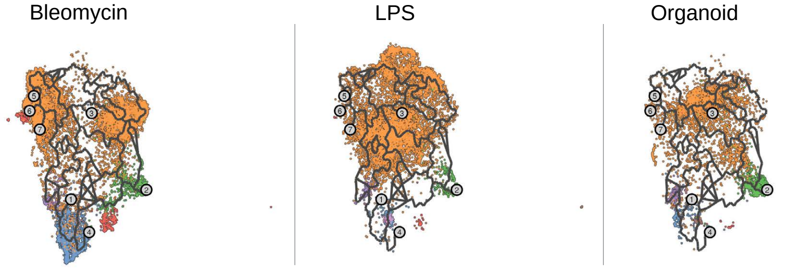
Bleomycin
LPS
Organoid

F.

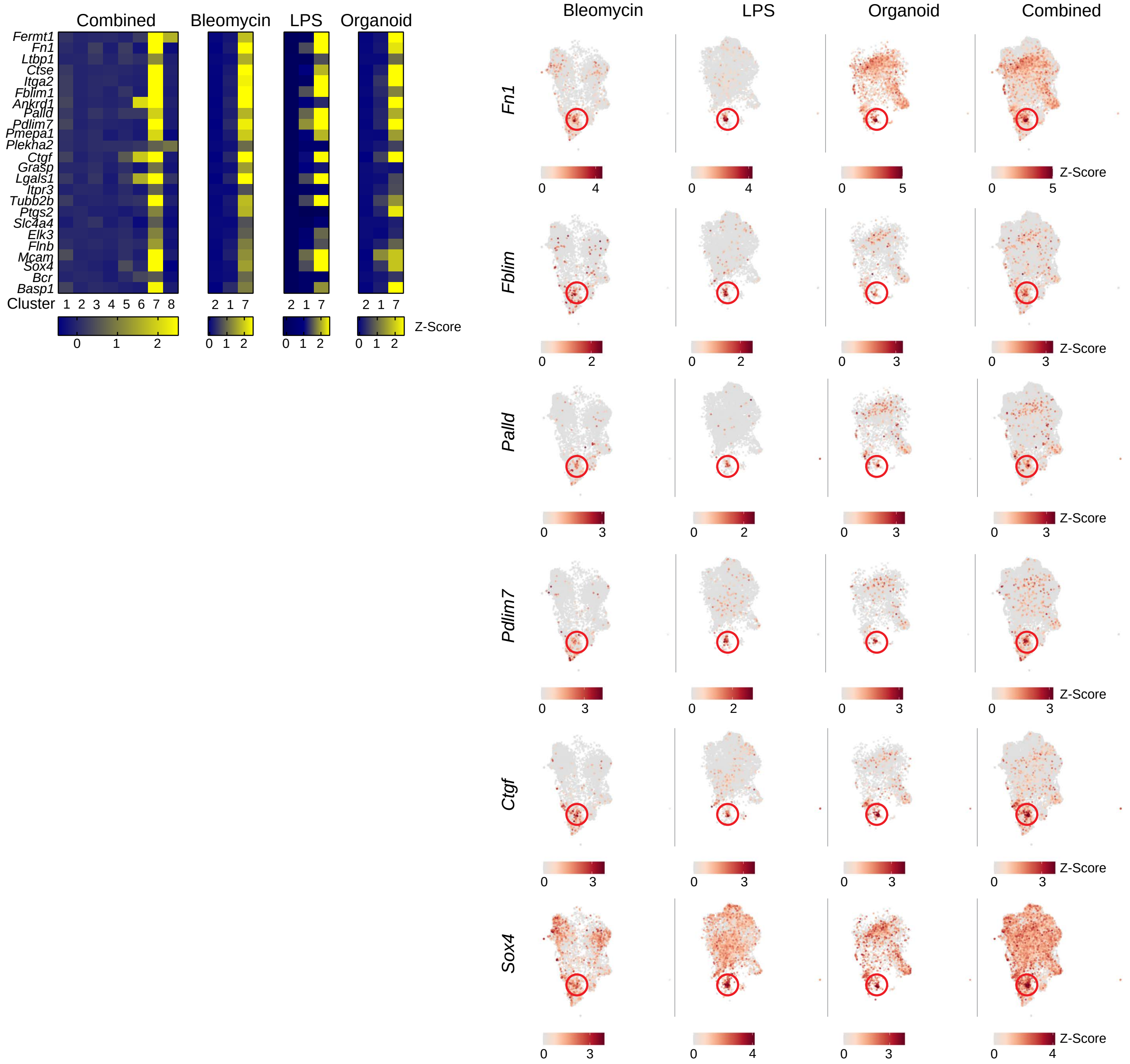
Combined
Bleomycin
LPS
Organoid
Fermt1
Fn1
Ltbp1
Ctse
Itga2
Fblim1
Ankrd1
Palld
Pdlim7
Pmepa1
Plekha2
Ctgf
Grasp
Lgals1
Itpr3
Tubb2b
Ptgs2
Slc4a4
Elk3
Flnb
Mcam
Sox4
Bcr
Basp1
Cluster 1 2 3 4 5 6 7 8
2 1 7
2 1 7
2 1 7
Z-Score
0 1 2
Bleomycin
LPS
Organoid
Combined
Fn1
Fblim
Palld
Pdlim7
Ctgf
Sox4
Z-Score

G.

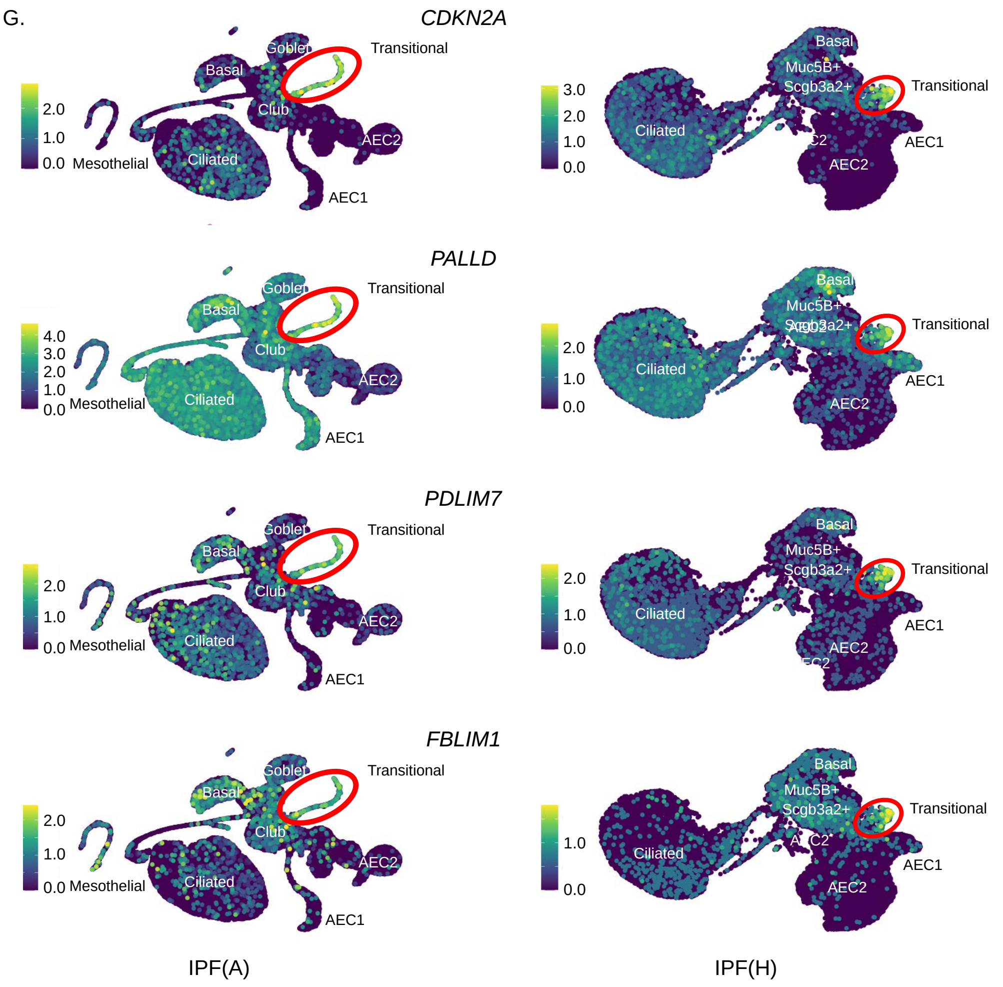
CDKN2A
PALLD
PDLIM7
FBLIM1
Transitional
Goblet
Basal
Club
Ciliated
Mesothelial
AEC2
AEC1
Muc5B+
Scgb3a2+
IPF(A)
IPF(H)

H.

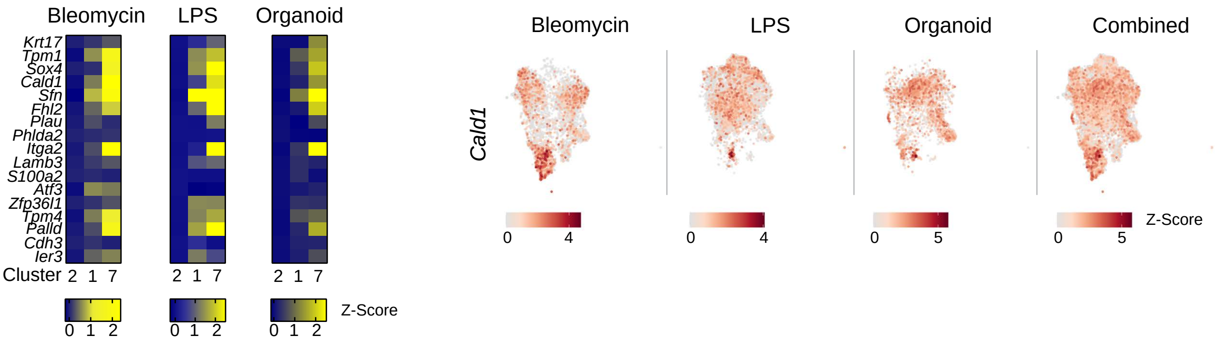
Bleomycin
LPS
Organoid
Combined
Krt17
Tpm1
Sox4
Cald1
Sfn
Fhl2
Plau
Phlda2
Itga2
Lamb3
S100a2
Atf3
Zfp36l1
Tpm4
Palld
Cdh3
Ier3
Cluster 2 1 7
Z-Score
Cald1

I.

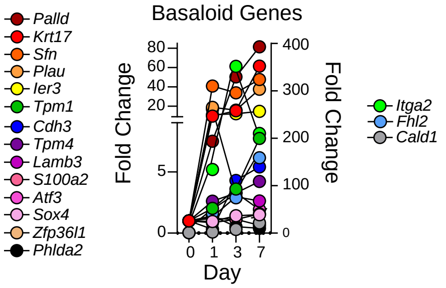
Basaloid Genes
Palld
Krt17
Sfn
Plau
Ier3
Tpm1
Cdh3
Tpm4
Lamb3
S100a2
Atf3
Sox4
Zfp36l1
Phlda2
Itga2
Fhl2
Cald1
Fold Change
Day

J.

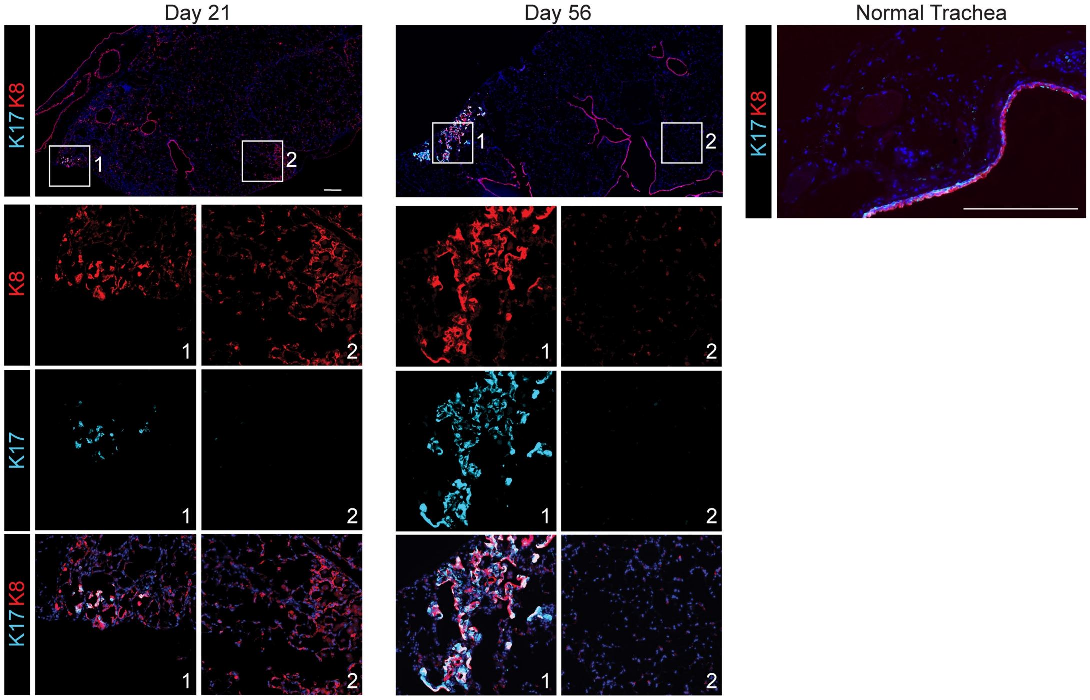

Day 21
Day 56
Normal Trachea
K17 K8
K8
K17
K17 K8
1
2

K.

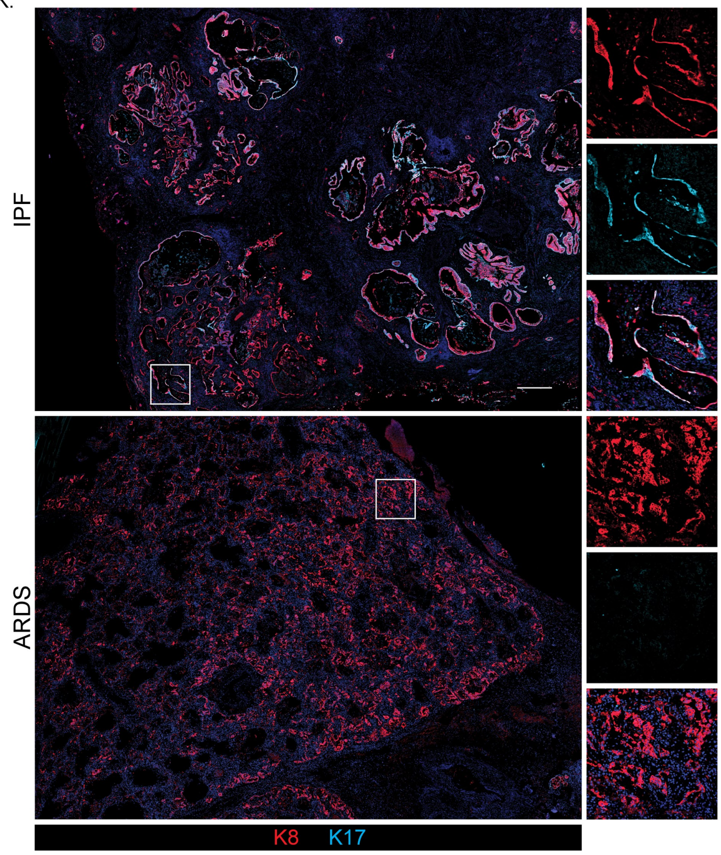

IPF
ARDS
K8 K17
